# Using Implementation Science to Improve Health Care Access and Quality for People With Traumatic Brain Injury–Related Morbidity (I-HEAL): Protocol for a Translational Multiproject Program Award

**DOI:** 10.2196/79738

**Published:** 2026-03-06

**Authors:** Risa Nakase-Richardson, Jolie N Haun, Megan Moore, Jennifer Bogner, Jeanne M Hoffman, Kristen Dams-O'Connor, Jill Coulter, Tracy Kretzmer, Aaron M Martin, Marc A Silva, I-HEAL Consortium, Sonia Arellano

**Affiliations:** 1 Department of Neurosurgery, Brain, and Spine, Morsani College of Medicine, University of South Florida, Tampa, FL, United States; 2 Tampa VA Research and Education Foundation, Tampa, FL, United States; 3 Research and Development Service, James A. Haley Veterans' Hospital, Tampa, FL, United States, 1 813-406-3664; 4 Division of Epidemiology, Department of Internal Medicine, Spencer Fox Eccles School of Medicine, University of Utah, Salt Lake City, UT, United States; 5 University of Washington School of Social Work, Seattle, WA, United States; 6 Department of Physical Medicine and Rehabilitation, College of Medicine, The Ohio State University, Columbus, OH, United States; 7 Department of Rehabilitation Medicine, University of Washington School of Medicine, University of Washington, Seattle, WA, United States; 8 Department of Rehabilitation and Human Performance, Icahn School of Medicine at Mount Sinai, New York, NY, United States; 9 Department of Neurology, Icahn School of Medicine at Mount Sinai, New York, NY, United States; 10 Department of Neuroscience, Icahn School of Medicine at Mount Sinai, New York, NY, United States; 11 Stakeholder with living experience of a caregiver for a person with TBI, Boston, MA, United States; 12 Mental Health & Behavior Sciences, James A. Haley Veterans' Hospital, Tampa, FL, United States; 13 Department of Psychiatry and Behavioral Neurosciences, Morsani College of Medicine, University of South Florida, Tampa, FL, United States; 14 See Acknowledgments

**Keywords:** brain injuries, traumatic, health care quality, health care access, health care evaluation, community-based participatory research, veterans, military personnel

## Abstract

**Background:**

People with traumatic brain injury (TBI) morbidity (impaired cognition and behavioral regulation) and polytrauma comorbidity (depression, posttraumatic stress disorder [PTSD], chronic pain, and sleep disorders) experience health care inequities. Among Veterans and Service Members (V/SMs), TBI morbidity or polytrauma comorbidity may impact access and meaningful engagement in the high-quality health care needed to reduce poor health care outcomes. The National Academy of Science, Engineering, and Medicine Report on Accelerating Progress in TBI highlights a dearth of implementation science research in TBI that may help overcome health care access challenges. Implementation science uses a mixed methods approach to understand, implement, and examine outcomes associated with using evidence-based care in practice.

**Objective:**

The I-HEAL (Improving Health Care Access and Engagement for Veterans and Service Members with TBI Morbidity) protocol includes 4 synergistic projects with the goal of addressing key knowledge gaps that will improve access and engagement in high-quality, evidence-based health care services for V/SMs with TBI morbidity. Collectively, the 4 projects propose to: (1) adapt existing interventions to promote access and engagement in health care; (2) engage stakeholder communities to maximize uptake and translation; (3) promote research translation that informs policy and practice through knowledge translation products and deliverables targeting key partners (clinicians, V/SMs, caregivers, policymakers, and researchers); (4) facilitate research and implementation to enhance access to high-quality health care for V/SMs with TBI-related morbidity; and (5) foster the development of early/mid-career researchers in advancing implementation science research on access to care for V/SMs with TBI.

**Methods:**

Project 1 will involve the development of a nudge intervention (electronic health care reminder) for providers to engage health care proxies when interacting with cognitive disability at risk for poor health care engagement. Project 2 will involve the development of a provider toolkit of adaptations of guideline-endorsed behavioral health interventions for common polytrauma comorbidities to meet the needs of cognitively impaired individuals. Project 3 will involve the adaptation and dissemination of evidence-based team interventions for managing maladaptive behaviors after TBI. Project 4 will involve evaluation and recommendations for policy for virtual health modalities among persons with TBI and polytrauma comorbidity.

**Results:**

I-HEAL has been funded as an implementation science Focused Program Award by Congressionally Directed Medical Research Programs, and start-up activities began in October 2023. All 4 projects are currently underway with funding through September 2027. Project 1 has enrolled 48 participants, and project 3 has enrolled 34 participants through September 2025.

**Conclusions:**

TBI is associated with increased health care utilization, comorbid health conditions, and premature mortality. This study has proposed to utilize strategies from the implementation science field to help overcome barriers to physical and psychological health care in order to reduce health care disparities associated with TBI disability.

## Introduction

### Focus Areas

The ability to access and engage in high-quality, evidence-based health care is critical to achieving positive health outcomes. Traumatic brain injury (TBI) morbidities, such as cognitive impairment and behavioral dysregulation, influence a person’s ability to access high-quality health care. This proposal uses innovation development to adapt systems of care to improve access and/or engagement in evidence-based care for persons with TBI morbidity in order to achieve improved health outcomes. The proposal focuses on a gap highlighted in the National Academies of Science, Engineering, and Medicine (NASEM) report on accelerating progress in TBI, specifically, promoting research translation using implementation science [1]. The individual studies in the proposal are a series of innovations to improve access to health care by overcoming barriers that have led to unmet care needs, delivery of non–evidence-based care, and subsequent health care disparity for Veterans and Service Members (V/SMs) with TBI-related morbidities (cognition, behavior, and psychological functioning). The individual studies uniquely target supply and demand dimensions that influence health care access to optimize outcomes by utilizing implementation science interventions paired with a community-based participatory action approach. Brief descriptions of the projects are as follows:

- For those with significant cognitive impairments, project 1 will develop a systems intervention to cue providers to include health care proxies in appointments to maximize compliance.
- For those with cognitive impairments, project 2 will develop provider resources to adapt evidence-based behavioral health therapies for common comorbid conditions (posttraumatic stress disorder [PTSD], depression, sleep disorders, and chronic pain) to maximize engagement.
- For those with maladaptive behaviors, project 3 will develop team-based resources to increase workforce capacity in delivering evidence-based behavior management, thus decreasing reliance on pharmacological restraints and denied access to rehabilitation services.
- For those with all types of TBI burdens, project 4 will generate data-driven policy recommendations to adapt universal virtual health care mandates to accommodate the needs of persons with TBI.

Individual projects will interact with 3 robust cores: Implementation Science Core (ISC), Website and Data Management Core (WDMC), and Community Engagement Council (CEC), which will include key partners representing the end users of each innovation proposed. As requested in the funding opportunity announcement, this proposal will address the implementation of evidence-based treatments and engage partners to promote the adoption of evidence-based care for optimal outcomes after military-related TBI. Multimedia Appendix 1 provides information on the impact and relevance of the proposed study for military health.

### Overarching Challenge

Our work in the National Institute on Disability, Independent Living, and Rehabilitation Research (NIDILRR) and Veterans Affairs (VA) TBI Model Systems (TBIMS) has highlighted that persons with TBI and greater neurologic morbidity are 6.9 times more likely to die than the general population, resulting in a 12.2-year reduction in life expectancy [2]. When examining service utilization, we found that those with greater neurologic burden experienced a 2-fold increase in rehospitalization rates in the first year after TBI compared to those with a lesser neurologic burden. V/SMs have higher rates of rehospitalization (44%) compared to civilians in the TBIMS [3]. Further, those with greater neurologic burden have higher rates of death and have distinct causes of early mortality (self-inflicted death and preventable injuries) across groups [4,5]. TBI morbidity (impaired cognition and behavioral regulation) coupled with polytrauma comorbidity (depression, PTSD, chronic pain, and sleep disorders) can impact access to and meaningful engagement in high-quality health care to reduce these poor health care outcomes [6]. This disparity is further evidenced by our work highlighting unmet health care needs in chronic stages [7] and comorbidities associated with worse outcomes [8]. The recent NASEM report on accelerating progress in TBI recognizes and calls for action to improve health care access for persons with TBI [1]. Poor access to health care is an inequity that can be addressed by using implementation science. The NASEM highlights a dearth of implementation science research in TBI that could help improve access and close the quality-of-care chasm experienced by persons with TBI disability [1].

### Overarching Solution

There is a growing recognition of significant delays in research translation across scientific fields. The emerging field of implementation science has been recognized as a critical field in the research translation process. Implementation science is the scientific study of methods and strategies that facilitate the uptake of evidence-based practice and research into regular use by practitioners and policymakers. Implementation science is complex and requires the use of mixed methods approaches to understand, implement, and examine outcomes associated with promoting the use of evidence-based care in practice. The field also recognizes that the use of evidence-based practice requires adaptations or innovations in how care is delivered (to retain technical quality and fidelity) across health care systems (eg, VA, Department of Defense [DOD], civilian, rural, and urban settings) and populations (eg, disabled vs healthy cohorts). Our work has shown that persons with TBI-related morbidity and comorbidity experience decreased access to health care in general, including known evidence-based treatments for physical and psychological health care. The purpose of this proposal is to address this health care inequity informed by the VA Quality Enhancement Research Initiative (QUERI) Implementation Roadmap, which defines 3 distinct stages to implementation science research (preimplementation, implementation, and sustainment) [9]. All projects fall in the preimplementation phase (adaptation and innovation to prepare for implementation), which often includes identifying partners, understanding needs, and developing knowledge products to facilitate the next stage (implementation). The measurable primary endpoints for this preimplementation phase are presented as milestones in the statement of work of I-HEAL (Improving Health Care Access and Engagement for Veterans and Service Members with TBI Morbidity) (Multimedia Appendix 2).

Knowledge products that synthesize scientific evidence from the literature or represent innovations in health care are critical to timely translation efforts. The VA QUERI Roadmap highlights phases of translation for bringing evidence-based health care to patients [9]. In the preimplementation phase (focus of this proposal), there is a need for knowledge products that clarify the problem being addressed, best treatment practices, and partners involved. The knowledge products generated across cores and projects (using community-based participatory research [CBPR]) will be informed by conceptual frameworks used in the field of implementation science, as described in the core descriptions. The second implementation phase includes preparing for and conducting implementation to promote the use of evidence-based or innovative care. The sustainment phase evaluates long-term impacts and assignment of implementation ownership. Given the scope of the funding opportunity announcement, the actual implementation (stage 2) and sustainment phases (stage 3) of the research will be addressed in future proposals and are described in the transition plan (Multimedia Appendix 3). Throughout this process, new questions arise, which require the generation of new data and reinitiation of the roadmap to address the evolution of health care delivery. The Focused Program Award (FPA) will generate products relevant for the preimplementation and implementation phases of this model. Knowledge products will be produced targeting key partners in the adoption of the study findings, which include (1) other scientists, (2) clinicians (physicians, behavioral health specialists, physical/occupational/speech therapists, etc), (3) policymakers, (4) hospital administrators, (5) trainees, and (6) persons with TBI and their families. As the products are finalized, they will be hosted on the study website, which will serve as a repository of tools (I-HEAL Toolshed) available to the public.

### Overall Program Synergy and Objectives

#### Developing Knowledge Products to Improve Health Care Access

The I-HEAL proposal includes 4 synergistic projects with the goal of addressing key knowledge gaps that will improve access and engagement in high-quality, evidence-based health care services for V/SMs with TBI morbidity.

#### Overarching Objectives of the FPA

Collectively, the 4 projects propose to accomplish the following overarching objectives of the FPA: (1) adapt existing interventions to promote access and engagement in health care; (2) engage stakeholder communities to maximize uptake and translation; (3) promote research translation that informs policy and practice through knowledge translation products and deliverables targeting key partners (clinicians, V/SMs, caregivers, policymakers, and other researchers); (4) facilitate research and implementation to enhance access to high-quality health care for V/SMs with TBI-related morbidity; and (5) foster development of early/mid-career researchers in advancing implementation science research on access to care for V/SMs with TBI to expand the implementation science workforce in this understudied, underutilized field.

#### Framework Informing the Mechanistic Targets and Impacts of Project Interventions

The proposed studies will be organized around a patient-centered conceptual framework of accessing evidence-based health care services to achieve optimal health outcomes [10]. The framework highlights how the health care environment (supply side, top) and the community that it supports (demand side, bottom) are multidimensional and interact to influence health care access [10]. The core elements of this model include having a health care need, the perception of the health care need, the desire for treatment, seeking and reaching health care, and utilizing health care to achieve an optimal outcome. The projects have unique interventional targets in the health care system (provider, team, system, and policy) to improve access and increase impact aligned with the best practices for addressing disparities [11]. The studies proposed in the FPA will target health care access dimensions on either the health care supply or consumer demand side that are known to be barriers arising from TBI-related morbidity, such as cognitive impairment and behavioral dysregulation. Each study will target a health care access dimension with primary and secondary targets. The interventions address TBI morbidity (impaired cognition and behavioral dysregulation) and high-frequency polytrauma comorbid conditions for military-related TBI, such as chronic pain, sleep disorders, and psychological health [12]. Project deliverables will be used to inform translational steps to promote dissemination and uptake across health care settings (ie, future implementation studies involving federal agencies such as VA, Agency for Healthcare Research and Quality, National Institutes of Health [NIH], and Patient-Centered Outcomes Research Institute [PCORI]). The framework and alignment with proposed projects are presented in Table 1.

**Table 1.** Access to the care conceptual framework and alignment with proposed projects.

Focused Program Award proposed projects	Health care supply dimensions of health care access					Consumer demand dimensions of health care access				
	Approachability	Acceptability	Availability and accommodation	Affordability	Appropriateness and technical (high) quality	Ability to perceive the need for services	Ability to seek services	Ability to reach services	Ability to pay for services	Ability to engage in high-quality care
Project 1^a^	Secondary impact	Secondary impact	Secondary impact	Secondary impact	Primary impact	Secondary impact	Secondary impact	Secondary impact	—^b^	Target
Project 2^c^	Secondary impact	Secondary impact	Secondary impact	Secondary impact	Primary impact	—	—	Secondary impact	—	Target
Project 3^d^	Secondary impact	Secondary impact	Target	Secondary impact	Secondary impact	—	—	Primary impact	—	Secondary impact
Project 4^e^	Secondary impact	Secondary impact	Target	Secondary impact	Secondary impact	Secondary impact	Secondary impact	Secondary impact	Secondary impact	Primary impact

a Cognitive flag to nudge providers to include family in health care delivery.

b Not applicable.

c Cognitive adaptation toolkit for behavioral health interventions.

d Adapting evidence-based behavioral treatment for managing challenging behavior.

e Understanding virtual health care accommodation needs for traumatic brain injury.

## Methods

### Ethical Considerations

The Institutional Review Board (IRB) approved a combined protocol for projects 1‐3 on August 12, 2024. Aim 4.1 is a secondary analysis from a larger existing IRB-approved study (December 2, 2019; “Characterization and treatment of chronic pain after moderate to severe traumatic brain injury: a qualitative study” [PR00039496]). All principal investigators (PIs) are currently on the existing IRB-approved study. Completion of aim 4.1 will require a modification to the parent study to acknowledge a new funding source for the secondary analysis. Informed consent will be obtained for all participants in projects 1-3, with the ability to opt out at any point. During consent, participants will be assured of their rights and privacy. Participants in focus groups and interviews will be compensated US $50. Participants in the online survey will be randomly selected to receive US $50. The consent of participants from the original research project covers project 4's secondary analysis of existing data. All reported data will be deidentified and in aggregate form.

### Oversight

This research project has been reviewed and found to align with the mission of the VA and be scientifically valid, and has been reviewed by all appropriate subcommittees to ensure the safety of the study participants and VA staff. Approval has been granted by a convened board review of the James A. Haley Veterans’ Hospital Research and Development Committee (IRBNet #1804543). I-HEAL will be reviewed at least annually or as otherwise required by the appropriate Research & Development Committee or subcommittees.

### Project Core Descriptions

#### ISC Details

The I-HEAL FPA will extend the successful collaboration among study investigators to advance implementation science within the field of TBI. The goals of the ISC are as follows:

1. Support individual projects from conceptualization and support methodological development, qualitative data collection/analysis, interpretation of the findings, and dissemination using integrated conceptual frameworks to inform methodological approaches and knowledge development.
2. Support implementation mentorship, training, and professional development for early career researchers and TBI-focused scientists to enhance workforce capacity in implementation science.
3. Support dissemination efforts of knowledge translation products and support the overall development of the I-HEAL Toolshed with partners in the CEC.

##### ISC Individual Project Support

###### Overview

The ISC will use broad-based efforts within I-HEAL and across projects to inform rigorous implementation science methodologies and outcomes. The ISC will work with the other I-HEAL cores to identify evidence-based strategies that shorten the time between evidence production and implementation into practice and policy, to promote access, and to ensure quality improvement (QI) in health care and outcomes by (1) collaborating with project teams to assist with qualitative data collection, data analysis, and dissemination throughout and after project completion; (2) iteratively assessing and responding to the needs of projects to ensure that the projects optimize implementation and translation opportunities; (3) defining and documenting the best practices in implementation science methods, deliverables, and outcomes; and (4) supporting product and Toolshed development for key stakeholder audiences to accelerate dissemination and implementation of evidence-based practices and maximize impact.

**Figure 1.**
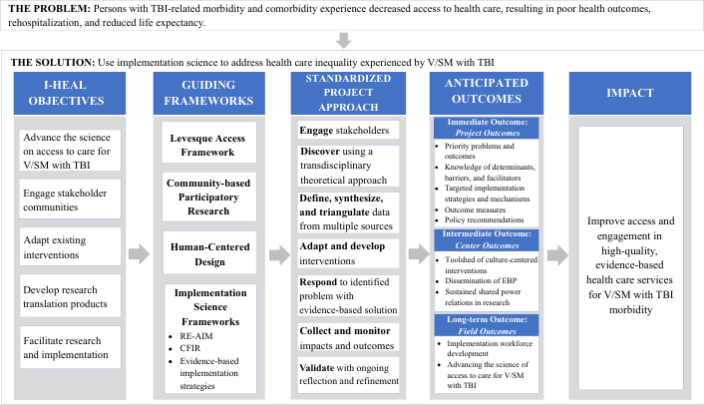
The multifactorial solution of I-HEAL (Improving Health Care Access and Engagement for Veterans and Service Members with TBI Morbidity) for addressing health care access inequality among Veterans and Service Members (V/SMs) with traumatic brain injury (TBI). CFIR: Consolidated Framework for Implementation Research; EBP: evidence-based practice; RE-AIM: Reach, Effectiveness, Adoption, Implementation, Maintenance.

In alignment with the VA QUERI Implementation Roadmap, the proposed I-HEAL objectives and projects are in the preimplementation phase, with a focus on access to care for persons with TBI, as guided by the Levesque Access Framework to support access to care for persons with TBI. As illustrated in Figure 1, the I-HEAL team will achieve the I-HEAL objectives by conducting the proposed projects to support adaptation and development of products and outcomes that increase access to evidence-based care for persons with TBI. To operationalize the I-HEAL projects, protocols, processes, and outcomes, each project will take a transtheoretical approach to integrate (1) CBPR, (2) human-centered design (HCD), and (3) an integrated implementation science framework (Reach, Effectiveness, Adoption, Implementation, Maintenance [RE-AIM]; Consolidated Framework for Implementation Research [CFIR]; and evidence-based implementation strategies).

###### HCD Approach

The proposed I-HEAL projects align with the preimplementation phase of the QUERI Roadmap through the development of knowledge products from published literature and other sources. An HCD process will guide the design and implementation of the proposed I-HEAL projects. HCD is an approach that “centers” end users (ie, the people who will ultimately use or benefit from a product or service) by integrating them into every aspect of the design process, thus strengthening the impact and utilization of the product or service [13-15]. HCD can combine mixed methods with novel efforts to engage end users in an iterative design process. Each of our projects’ aims aligns with the 4-phase Double Diamond HCD model [16]. This model directs researchers and designers to engage end users to first *discover* and *define* a problem and then take focused action to *develop* and *deliver* (ie, validate) a solution [17]. HCD supports the process of making high-quality evidence-based tools that are effective, feasible, and aligned with the needs and goals of users and partners.

###### Implementation Science Framework

I-HEAL and its proposed projects will leverage an integrated implementation science framework that includes RE-AIM, CFIR, and evidence-based implementation strategies. The RE-AIM framework focuses explicitly on issues and steps in the implementation process that may improve/impede the desired impact. Due to the preimplementation nature of the proposed projects, though not immediately relevant, RE-AIM provides an overarching framework for the lifetime of these projects after the FPA, down the implementation pipeline. Within the phases of RE-AIM, CFIR is a practical evidence-based implementation framework to examine and organize determinant factors that influence implementation outcomes across the following 5 domains: intervention/project characteristics, outer and inner settings, characteristics of individuals, and the process of implementation. At this point, evidence-based implementation strategies can be mapped based on relevance to determinant implementation factors.

Collectively, these theoretical frameworks inform the I-HEAL conceptual lens (see Figure 1) across projects to standardize our approach to implementation over time.

### ISC Mentorship, Training, and Professional Development

#### Overview

The ISC will support mentorship, training, and professional development for the next generation of rehabilitation scientists to support the development of a TBI implementation science workforce. Our objectives are to (1) provide mentorship and training for the next generation of TBI-focused implementation scientists to democratize implementation and knowledge translation expertise in the field of TBI research and (2) identify and facilitate implementation-focused training and professional activities for I-HEAL investigators, which are available within and beyond the VA/DOD network.

These 2 training objectives will be accomplished using the following strategies:

1. Project-based mentorship: Expedite and align the mentorship process by matching prospective mentees to mentors with research portfolios and content expertise, as evidenced by the teams of the 4 projects. These individualized practical experiences will support mentees in developing research programs and co-authoring with mentors.
2. Structured I-HEAL ISC training: Implement a planned structured curriculum through regular I-HEAL meetings that will provide (1) dedicated time to support and train with mentors, (2) dedicated research presentations, (3) training developed by RN-R and JNH [18] (ie, implementation science theoretical models, implementation research logic modeling, implementation planning, and playbook development), and (4) evidence-based resources available from the VA QUERI [19]. The *Using Implementation Facilitation to Improve Healthcare* Manual [20] is an evidence-based training manual designed to “support implementation of evidence-based practices and programs and other clinical innovations.” Collectively, the I-HEAL training program will address an array of topics, including but not limited to implementation research designs; conceptual frameworks and theories; mixed methods and evaluation methods; assessment of organizational, provider, and veteran readiness; and customization of strategies to overcome significant barriers.
3. Training opportunity registry: Develop and sustain an I-HEAL training registry to identify training and professional development opportunities (eg, workshops, seminars, webinars, etc) with relevant details (ie, training dates, trainee eligibility, costs associated with training, etc).
4. Professional presentation opportunities: ISC mentor and mentee attendance at professional conferences will be a priority benchmark each year of the funded FPA.
  - ISC training transition plan: Senior investigators will provide regular team training workshops for team members, where early career investigators will attend and learn methods and areas of expertise. Collectively, the I-HEAL group (senior and early career scientists) will work to enhance and tailor training. These trainings will be developed, marketed, and disseminated as workshops to professional venues. These workshops will be co-presented by senior and early career investigators and will eventually transition leadership to early career I-HEAL scientists. These efforts will advance the skills of I-HEAL team members as well as support workforce development in the TBI and rehabilitation professional communities.
5. Mentee research experience translation: I-HEAL will support early career scientists and TBI subject matter experts (SMEs) to successfully translate their research experience with I-HEAL into innovative proposals and manuscripts throughout and after the FPA, by fostering a dynamic, learning environment and providing opportunities to develop core competencies (eg, communication, professional and scholarly development, partnered research, and the application of dissemination and implementation science).
  - Training in partnered research: Mentees are full members of I-HEAL, and the proposed FPA will create a collaborative research environment with a variety of investigators and partners. While participating in partnered research with mentors, mentees will have opportunities to interact with local, FPA CEC partners as well as community, academic, and clinical partners. Investigators will actively assist trainees to develop their own partnerships through a variety of methods, such as modeling behaviors, making introductions, helping them to prepare for meetings with prospective partners, and debriefing after interactions with partners. RN-R, JNH, and JMH have strong backgrounds in professional mentorship and will use their developed mentorship strategies to support I-HEAL mentorship and professional advancement.

#### ISC Dissemination

Our goal is to spread I-HEAL’s impact, focusing on health systems and the delivery of TBI care within VA/DOD and beyond. I-HEAL’s dissemination efforts will be led by the ISC, with strategic planning that targets audiences and channels with consideration of the impact. Target audiences are (1) I-HEAL partners, leadership, CEC partners, and investigators; (2) DOD and VA funding agencies; (3) associated VA leadership; (4) university and educational affiliates; (5) policy groups such as professional organizations and federal agencies; (6) local communities; and (7) V/SM service organizations through the CEC. The channels of communication will minimally include (1) written communication (eg, peer-reviewed publications, issue briefs, formal reports, national magazines, and newsletters), (2) oral communication (eg, scheduled and ad hoc face-to-face and telephone meetings with operations partners and steering committee members, professional meetings, annual VA research day, cyber seminars, and grand rounds), (3) electronic communication (eg, social media and the I-HEAL Toolshed; I-HEAL will have a social media presence and explore implementation and dissemination strategies to amplify dissemination), and (4) product development (eg, toolkits, playbooks, and multimedia products). To optimize dissemination, the ISC has developed a publication pipeline protocol that strategizes and documents benchmarks, timelines, and roles to support accountability and productivity. Impacts of these broad-based efforts include but are not limited to increased awareness, trust, and veteran and employee engagement; distribution of knowledge; and relationship building and networking with stakeholder groups, study participants, mentees, and potential new investigators.

#### ISC Potential Impact

**Figure 2.**
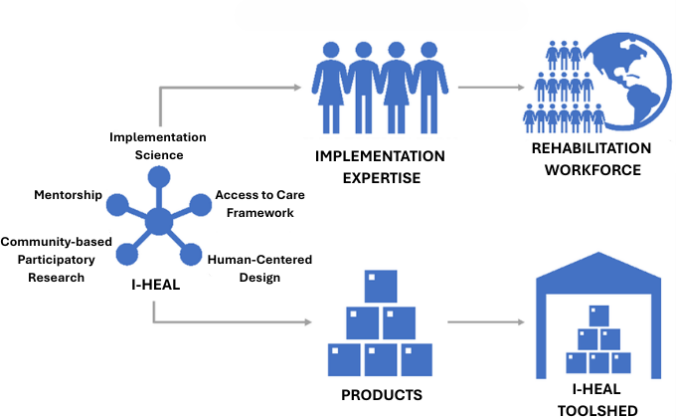
Overview of the impact of the Implementation Science Core. I-HEAL: Improving Health Care Access and Engagement for Veterans and Service Members with TBI Morbidity.

An overview of the potential impact is provided in Figure 2. Across cores and projects, this proposal has gathered a transdisciplinary team of scientists who have collectively demonstrated a history of rapid development and implementation of projects with the ability to rapidly pivot and assemble strong approaches to evaluate priorities in TBI and implementation. I-HEAL projects will contribute to (1) improving access and outcomes for persons with TBI and relevant partners; (2) tailoring policy recommendations, products, and outcomes for persons with TBI, hosted on the I-HEAL Toolshed; (3) advancing the science of implementation, translation, and dissemination; and (4) expanding the implementation and dissemination of science expertise for I-HEAL TBI scientific team members, which will ultimately support the implementation of professional development for workforces that specialize in TBI research.

### CEC Partnership Plan and Outreach Plan

#### Overview

This proposal was developed by our team of investigators, policy and professional partners (PPPs), and lived experience partners (LEPs). We have extensive experience engaging in CBPR together. The partnerships outlined here are built on long-term relationships and ongoing collaborations. Aligning with the principles of CBPR, we ground our work in the shared values of co-learning, mutual benefit, and community-driven solutions and practices for addressing the collaboratively identified problem of access to care for persons with TBI [21,22]. The current proposal grew out of years of working closely with professionals and LEPs who identified access to care as the most pressing problem facing V/SMs with TBI and their caregivers. Members of our team have collaborated on several successful projects over many years, including a large-scale randomized controlled trial of a patient-centered care management intervention for persons with TBI (PCORI R-1511‐33005), the BeHEALTHY model for chronic brain injury care (NIH-NINDS U24NS095871), Improved Understanding of Medical and Psychological Needs (I-MAP) in V/SMs with chronic TBI (DHA Contracting Office W91YTZ-13-C-0015, HT0014-19-C-0004, HT0014-21-C-0012, and HT0014-22-C-0016), and the BRITE Study (improving transition from inpatient rehabilitation following TBI; NCT03422276).

Building on these existing partnerships and the previously identified priority problem of access to care, we convened in 2022 in Tampa, FL, for a 2-day intensive retreat to develop the I-HEAL objectives, develop the proposed studies, solidify the partnership plan, develop the dissemination and outreach plan, and outline the functions of the CEC. See the CBPR statement for an overview of our engagement process, beginning with our existing partnerships and continuing throughout the stages of the proposal (Multimedia Appendix 4).

#### CEC Partnership Plan

The CEC partners, purpose, and processes for achieving deliverables, functions, and specific deliverables are outlined below. We have structured our partnership plan to reflect the importance of engagement with a broad range of individuals working to improve the lives of V/SMs with TBI. We have conceptualized the CEC to have 3 core partner groups with synergistic purposes. Important aspects of impactful science to address access to care for V/SMs with TBI are the voices of persons with lived experience of TBI, the system-level and disciplinary knowledge of successful policy and dissemination strategies, and the presence of individuals with expertise on the specific study issues. We have developed a CEC with expertise in each of these core areas. Each partner brings passion and commitment to improving the lives of V/SMs with TBI. They bring unique and complementary knowledge and will engage with all cores and individual projects to achieve the overall I-HEAL objectives.

#### CEC Partners and Purpose

**Figure 3.**
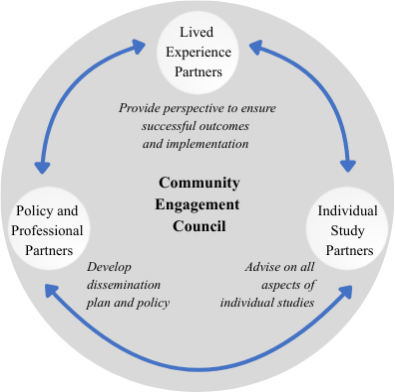
Community Engagement Council composition.

The composition of the CEC is presented in Figure 3. We have established a CEC comprised of persons from influential organizations representing professionals who will engage with I-HEAL products (PPPs) and individuals who can speak about their lived experience of TBI or caregiving for a loved one with TBI (LEPs). We have representation from members of key professional groups involved in health care policy development for VA and the DOD, and providers representing professional organizations (physiatry, social work, psychology, rehabilitation, and nursing) involved in the care of persons with TBI. We have engaged with a diverse group of LEPs with military service backgrounds and with experience navigating health care in military and civilian environments. Members include persons with TBI (V/SMs and civilians) with greater neurologic morbidity and their spouses. Each member has been selected because of their lived experience of the challenges being addressed in this proposal (individuals with brain injury and cognitive morbidity, legal guardians of V/SMs without decision-making capacity, caregivers of veterans with severe maladaptive behaviors living in the community, and individuals experiencing challenges in navigating the health care system). Many have served as engagement partners on other TBI research projects or as members of the Tampa VA Veteran Engagement Council. The PPPs will focus on dissemination and policy development, and the LEPs will focus on the impact to those with lived experience and the identification of potential facilitators and barriers to successful implementation and outcomes. In addition, individual project engagement partners (PEPs) have been identified by each proposed project team to support the specific needs of the projects.

#### CEC Processes

The CEC will be involved in all aspects of the proposed research implementing the I-HEAL objectives. Drs John Corrigan, MM, and Chaady Radwan will co-chair the council. Dr John Corrigan will be responsible for chairing the PPP quarterly meetings. The LEPs will elect a chair who will co-lead the monthly LEP meetings with Drs MM and Chaady Radwan. This group will meet more frequently to ensure that each member feels prepared to participate in the co-learning process and has adequate time to contribute their expertise to the research process using a CBPR approach (Multimedia Appendix 4). In years 1 and 4, all members of the council will meet in person with all other participants in center activities to allow for interaction as the studies begin (year 1) and allow for collaboration as results emerge and dissemination and policy development plans are solidified (year 4).

#### CEC Meeting Schedule

Information on the meeting schedule is provided in Multimedia Appendix 5. First, Dr MM (multiple principal investigator [MPI]) will oversee ongoing, bidirectional communication between the cores, the study teams, and the CEC by attending the all-hands calls, executive committee, steering committee, and core meetings. The co-chair of the LEP group will attend these meetings monthly. Second, Dr John Corrigan, MM, or Chaady Radwan will attend quarterly meetings with the project teams to monitor engagement needs. In addition, the study teams will rotate attending the meetings of the PPPs and LEPs based on their engagement needs as described in their project methodology below. The proposed engagement from study teams will be incorporated into regularly scheduled CEC meetings. It is expected that communication via email will occur between regularly scheduled meetings and that partners will be available on an as-needed basis between regular meetings. These meetings will be in addition to the in-person meetings in years 1 and 4.

#### CEC Functions

The CEC will be involved in all aspects of the proposal, including the planning, conduct, and dissemination of results. The planning of this proposal is based on over 25 years of patient-centered research conducted by our team members, an extensive literature review, discussions with our CEC partners, and a 2-day intensive retreat used to solidify the plan and partnership roles. Priority problems and outcomes have been identified through these processes. Moving forward and prior to the initiation of each study, the CEC will advise on consent forms, recruitment processes, study rollout, advertisements, and other materials provided to study participants. The CEC will participate in problem-solving for each study as issues arise. In particular, the LEPs will assist in the identification of barriers and facilitators to successful outcomes via engagement with the study design, progress, and data interpretation. They will provide their views on the results and data interpretation. The PPPs will provide insights into the policies and priorities of the constituents they represent as relating to each study and to the overall access to care for persons with TBI. The overarching goal of their collaboration is to ensure that the study results will be relevant, practical, and acceptable to members belonging to their respective professional organizations. Their involvement will be especially critical to the dissemination and policy development activities described later. The CEC will also assist in community outreach, dissemination, and policy implications. The LEPs will advise on the development of dissemination materials for individuals who have had a TBI and their family members. Together with our outreach and media specialist, they will co-develop social media posts, print materials, and brief multimedia presentations that will be used to disseminate study materials and findings throughout the course of the 4-year project (see the Outreach Plan subsection below). The PPPs will advise on successful dissemination strategies as well as relevant financing or other policy-related issues that influence the implementation and sustainability of products. We will leverage the significant leadership platforms in organizations within the VA and professional trade groups and the leadership of brain injury researchers to understand and advance policy and clinical practice guidelines emanating from the study results. We will work closely with the CEC to ensure engagement remains rooted in the principles of CBPR. We will utilize standard metrics of engagement success, including meeting attendance, self-reported empowerment within the group, and objective measures of engagement success pertinent to the completion of each study (see details in the CBPR statement in Multimedia Appendix 4).

#### Outreach Plan

Specifically, we will disseminate project updates and results via Twitter, Facebook, and outlets specific to V/SMs recommended by our LEPs. We will utilize multimedia, including video clips and visuals, which are accessible to a wide audience. These outreach efforts will occur at a minimum of once per quarter and as needed when exciting results emerge or other information is determined by the CEC and study teams to need dissemination. The CEC will work with the research teams for traditional academic outreach and dissemination via peer-reviewed papers, conferences, and reports to funders. A focus of our outreach plan is to work closely with our social media specialist to develop materials for social media and community-focused dissemination.

#### CEC Deliverables

The CEC has both process- and outcome-related deliverables. Process-related deliverables include measurable successful engagement via meeting attendance, participation, and self-reported empowerment. Co-learning, as measured by successful participation in research processes by CEC partners and successful incorporation of CEC advice and suggestions into projects, is a key deliverable. Outcome-related deliverables include inclusive consent and recruitment processes and documents that lead to optimal recruitment for each study; collaborative data analysis that results in meaningful use of data; community-informed dissemination materials to increase reach; and policy and practice guidance for successful dissemination. In addition, each study has identified key needs and deliverables that need to be met by the CEC. See Table 2 for an overview of the deliverables for the CEC and for each project’s engagement plan.

**Table 2.** Community Engagement Council deliverables.

Deliverable	Overall	Project			
		1	2	3	4
Measurable successful engagement	Yes	Yes	Yes	Yes	Yes
Co-learning related to research and community engagement	Yes	Yes	Yes	Yes	Yes
Optimal recruitment via inclusive consent and recruitment processes and documents	Yes	Yes	Yes	Yes	Yes
Meaningful data interpretation via collaborative data analysis	Yes	Yes	Yes	Yes	Yes
Community Engagement Council–informed dissemination materials	Yes	Yes	Yes	Yes	Yes
Successful dissemination via policy and practice guidance	Yes	Yes	Yes	Yes	Yes
Recruitment of focus group participants	No	Yes	No	Yes	No
Recruitment of survey participants	No	Yes	Yes	No	No
Toolkit or playbook development	No	Yes	Yes	Yes	No
Identification of resources for modifying behavioral health treatments	No	No	Yes	No	No
Formal review of the toolkit prototype or products	No	Yes	Yes	Yes	Yes

### I-HEAL WDMC Leveraging the TBI Model System Research Network Infrastructure

#### TBIMS Network Research Environment

The proposed series of projects will leverage the NIDILRR and VA TBIMS research network infrastructure. The TBIMS program is a longitudinal multicenter study that examines the course of recovery and outcomes following inpatient rehabilitation. This epidemiologic lifetime study, analogous to the Framingham study, has been funded for the past 30 years, with over 18,000 participants enrolled across 16 centers during inpatient rehabilitation for moderate to severe TBI. An elaborate administrative infrastructure developed by the TBIMS investigators supports collaboration via committees (ie, planning, research, knowledge translation, and data) and special interest groups (eg, disorder of consciousness and behavioral health). In addition, multicenter collaborative studies (module studies) are conducted in each funding cycle as part of infrastructure grants. In 2008, the NIDILRR and VA developed an interagency agreement to present the same program as the TBIMS at specialized rehabilitation hospitals within VA. Five polytrauma rehabilitation centers (PRCs) represent the rehabilitation arm of DOD trauma care. Owing to a memorandum of understanding between VA and the DOD, TBI rehabilitation care occurs at these 5 PRCs (Tampa, Richmond, Minneapolis, San Antonio, and Palo Alto). These 5 additional sites have been funded, increasing the TBIMS network to 21 centers, with over 19,000 civilians, veterans, and active-duty service members enrolled in TBIMS research. Most study PIs and co-investigators work within this infrastructure.

#### TBIMS National Data and Statistical Center

In addition to the network sites, the NIDILRR and VA fund the National Data and Statistical Center (NDSC) in each funding cycle. The research program at Craig Hospital in Denver, CO has successfully competed and won the NDSC award for the past 5 funding cycles (20 years) under senior leadership, including Drs Dave Mellick (director) and Jessica Ketchum (senior biostatistician). Craig Hospital has served as the data coordinating center for several extramural and intramural projects leveraging the TBIMS network. Moreover, it has served as the data management center for 36 module (multicenter) studies within the TBIMS network. Furthermore, it has served as the data coordinating center for 10 other multicenter studies funded by the DOD, VA, PCORI, NIDILRR, and NIH. The data management infrastructure and experience of investigators at Craig Hospital will be leveraged for this study.

#### I-HEAL Structure

The staff will plan, develop, manage, and secure the implementation of data systems and conduct all statistical analyses for the successful completion of relevant individual studies. The core will (1) work closely with the individual study PIs to ensure successful execution of the protocol and follow-up of all participants enrolled in individual quantitative data collection efforts across projects through the use of multiple communication strategies; (2) obtain, manage, and analyze data from relevant individual projects; (3) ensure adherence to the research protocol by participants and research staff through in-person and virtual data collector trainings alongside the ISC, regular teleconferences, and constant data validation to monitor the quality of data collection, record keeping, and documentation, and the accuracy of data entry; (4) provide statistical support, expertise, and oversight for the duration of the proposed studies; (5) coordinate communication between individual projects and cores as outlined in the implementation plan; (6) provide administrative and logistical support for the study; and (7) organize, maintain, and submit study data and deidentified study resources to a study repository upon the completion of the study.

#### Development of the Online Study Dashboard and Toolshed

We intend to create a web presence as a landing space for public and private dissemination of information [23]. The study website will allow for centralized storage of standard operating procedures (SOPs), manuals of operations, IRB documents, form versions, and contact information for staff across all study sites, obviating the need to solely rely on email to deliver information to project members. The study website will also allow all study personnel to securely access up-to-date dynamic study reports for information on the status of enrollment, data errors, and missing data. Automatic email reminders will be sent to all relevant personnel when new participants consent to the study, which will prompt them to schedule focus groups or key informant interviews. Moreover, personnel will receive notifications on the completion of data collection and other data quality updates.

#### Data Management and Biostatistical Innovations

The NDSC is funded until September 2026 (fifth 5-year funding cycle). The NDSC has had many accomplishments and innovations, including moving the TBIMS National Database to a web-based data management system; providing a web-based database and analytic support for TBIMS Collaborative Module studies; introducing new and innovative statistical techniques for analyzing the TBIMS National Database; developing a structured interview for TBIMS research follow-up; conducting intense quality support visits to individual TBIMS sites; achieving several data collection certifications, including certification for this study’s primary outcome; creating a dashboard of dynamic annual data reports; generating several data quality reports; implementing hundreds of computerized data checks; and maintaining an extensive SOP manual. Many of these technical and data management innovations will be incorporated into the procedures of this proposed study.

The WDMC will follow the best reporting standards to effectively display quantitative information visually. Based on the teaching of Tufte [24] and using the statistical package R and Shiny Server, we will produce a dynamic array of data and process reports that can best be understood by the data consumer.

The TBIMS environment is already functioning and is based on the best practices for a public web application, in which the web server sits outside the network in a demilitarized zone on a virtualized server. There will be only one connection between the web server and the database that flows through the firewall, which acts as a logical filter of traffic between the networks. In between these connections is an F5 web application firewall, which provides threat detection. Furthermore, the framework is 100% data-driven, meaning that the data entry forms are derived from information in the data dictionary.

Because the user interface is completely controlled by data, no code needs to be written to roll out a new data collection form, enabling a speedier and more efficient development-to-production time frame. These systems utilize a web front end (Linux web servers using ASP.NET Core, Angular, and web API) and a Microsoft SQL Server, which is encrypted using a FIPS 140.2 algorithm (the necessary level for government databases) and is consistent with the Federal Information Security Modernization Act legislation. Transmission of the data will also be encrypted using Transport Layer Security (TLS). Our technology solution is built on the security model of the CIA triad (confidentiality, integrity, and availability) using web technologies as a front-end system (where data are entered) and a SQL server as a back-end system (where data are stored).

#### Confidentiality

Confidentiality of the participants will be ensured through multiple measures. First, the database records will contain limited personal identifiers for use in contacting participants (eg, phone number). The web data system is set up in such a way that only authorized individuals using token protocols from ASP.NET 4.0 can access the study website. Additionally, each end user will have specific privileges to read/add/delete the data that are authorized by that center’s PI. Staff at the NDSC will have limited access to the data through the use of SQL server roles and active directory group membership. The staff will follow the method of least privilege permissions, in which all users have the minimum amount of system access that still allows them to perform their job functions. Any hardcopy data forms will be stored at the individual study sites and will follow IRB confidentiality protocols.

Representatives of the study sponsor are eligible to review study records, and this will be addressed with potential study participants during the enrollment and informed consent process. No sensitive information that is required to be reported to state or local authorities will be captured as part of this study. We will leverage the Craig Hospital Information Technology disaster recovery model, in which there are redundancies throughout the system, such as automatic and encrypted backups and co-location of servers in separate areas of the Denver metro area.

### Implementation Plan

#### Communication Strategy

Information on the communication strategy is provided in Multimedia Appendix 6. The management of operations for this proposed study requires close coordination of multiple functions carried out by numerous staff with distinctive qualifications. Forming executive and steering committees is essential to the management process. The executive committee (study MPIs and the overall project manager [PM]; see Multimedia Appendix 7 for project leadership) will oversee overall progress, communication with the funding agency, regulatory issues, and fiscal issues. The steering committee will be comprised of the study MPIs, PIs from all individual studies, core directors, and the centralized PM, who will provide support to both committees. An all-hands call will be established to include all study leaders (MPIs, cores, and study PIs) with all core and co-investigator personnel across the FPA. Individual projects and cores have their own meeting schedule and purpose. In-person meetings will be held in years 1 and 4 to provide an in-depth orientation and review of each project’s focus and progress to optimize CEC member input. The WDMC will establish an online secure website and dashboard that will alert the study team to consented participants for scheduling and follow-up.

#### Milestone Tracking

To ensure that the project will begin without delay, components of the proposal are included in the implementation plan as best practices/innovations by the NIH. Gantt charts for the overall FPA and for individual projects are included in the statement of work (Multimedia Appendix 2).

Regarding the utilization of project management software for milestone tracking for the overall program and individual studies, each core and individual study will utilize software, such as Click-Up or Microsoft Project, as a project management system. The PM will enter goals and tasks based on study milestone tasks (and due dates) and assign responsibility as appropriate. The project will be used for planning, management, tracking meeting agendas and notes, monitoring progress on a regular basis, and supporting other continuous QI activities. This system will be used to ensure that study milestones are met on time and to implement the evaluation plan. Additionally, the project will be linked to Microsoft Outlook and Microsoft Teams, which are available across all study sites for communication of study progress and upcoming events. The Microsoft Teams platform and dashboard will be used to conduct study meetings (to record content for later accessibility) and make available files, documents, and notes during all study-related meetings requiring the use of full-team review features. Decisions made and follow-up actions will be documented and archived for access by all team members. The PM will generate monthly reports to ensure the quality and timeliness of each task and identify tasks (at the PI, site PI, or core level) that require additional attention or staff adjustments. See the statement of work for detailed milestones and work activities across cores and individual projects (Multimedia Appendix 2).

#### Study Operating Procedures for Data Management and Quality Best Practices Across Projects

Over its 20-year history, the TBIMS NDSC has developed 31 SOPs for all aspects of study conduct (eg, identification of participants with TBI, guidelines for medical record abstraction, strategies for maximizing follow-up, handling unexpected events at follow-up, prevention and investigation of falsification of data, submission processes, performance target monitoring, branding, and authorship policies). Data quality and representativeness are monitored via 15 on-demand reports (eg, enrollment, follow-up, missing data, and underrepresented population recruitment and follow-up). All project PIs and the contact PI RN-R are currently funded TBIMS investigators and are familiar with these processes, which will be adapted for the proposed FPA. The data management infrastructure and SOPs developed at Craig Hospital will be leveraged for this study.

#### Monitoring of Performance Metrics for the Overall FPA and Individual Projects

During routinely scheduled meetings, as outlined in our communication strategy (Multimedia Appendix 6), updates regarding fiscal and logistical issues will be addressed. Project leadership teams will meet weekly during study initiation and transition to monthly meetings when study operations are well underway. Increased frequency will be added as needed. During these routinely scheduled meetings, the MPIs will resolve fiscal and logistical issues in a timely manner, including plans to proactively evaluate and prioritize study risks and issue corrective responses using TBIMS best practices. During these meetings, progress toward study milestones will be reviewed with the PMs to identify potential risks and issue corrective responses. On-demand reports are available to proactively identify risks. Study-specific reports are generated “on demand” regarding milestone tracking, including screening, enrollment, and the degree of data collection completion. Additionally, the DCC will generate monthly reports regarding follow-up rates, data missingness, and adverse event monitoring, if relevant. These reports will be reviewed during the executive committee meeting, with follow-up during the steering committee meeting. Project teams will also review reports weekly or as generated by the WDMC to proactively address potential risks. This mechanism of feedback has been highly successful in past trials completed in the TBIMS network with regard to the completion of data collection on schedule and the achievement of all study milestones well within the funding period.

#### Remediation Plans

For underperforming sites, TBIMS SOPs describe that remediation plans should be developed by site PIs to encourage review of local practices by study staff and individually developed plans in order to reach expected benchmarks for enrollment, follow-up data collection, and overall data quality. Remediation plans are expected for sites missing any of the benchmarks (eg, enrollment, follow-up, and missing data), using TBIMS data quality standards. The steering committee will review remediation plans for approval. Once executed, individual project performance will be monitored monthly for a quarter to determine improvement. If no improvement is observed, the study teams will engage one of the study cores (CEC, WDMC, or ISC) for additional engagement to overcome project challenges. Sites are accustomed to these standards and have had success in overcoming missed benchmarks with a remediation plan policy. RN-R has used this method across several trials, with success in achieving study milestones.

#### Dissemination of Findings

The ISC, CEC, and WDMC will collaborate throughout the funding period to facilitate communication of findings for various stakeholder communities. Consistent with our history, the overall FPA scientific products will be submitted for publication in leading scientific journals in sleep medicine (eg, *Chest*, *Sleep,* and *Journal of Clinical Sleep Medicine*) and rehabilitation (*Journal of Head Trauma Rehabilitation*, *Journal of Neurotrauma*, and *Archives of Physical Medicine and Rehabilitation*). Utilizing the infrastructure of the TBIMS Study Network, which includes a separately funded Knowledge Translation Center with an international web presence, the study team will propose and develop fact sheets on the Model Systems Knowledge Translation Center (MSKTC) website. These fact sheets are accessed by patients, caregivers, and clinicians from around the world for consumer-friendly information about TBI. Dissemination success is evidenced by the number of downloads annually. The broad reach of the MSKTC is evident by the 21,967 external web pages linked to the MSKTC site from over 4000 unique domains, with 39% in the area of TBI. From January 2022 through October 31, 2022, the MSKTC website was accessed 1,825,439 times (752,047 for TBI-only content). Among those accessing the MSKTC website, 332,065 were from the United States, with the remaining from 48 other countries, highlighting the international success of the MSKTC as a dissemination venue. Many of the products are now available in Spanish and formatted in HTML to be cell-phone accessible. We will propose updates to existing relevant fact sheets and new content in year 4. Finally, standard dissemination activities include presentations at the DOD’s annual professional conference, Military Health System Research Symposium, and other venues as indicated by the steering committee.

#### Strategic Partnership to Rapidly Translate Findings

The strategic selection of partners in the CEC will facilitate the rapid translation of knowledge products developed in the proposal. We have partnered with end users of the products to maximize usability and implementation in real-world VA, DOD, and civilian health care settings. We have partnered with professional organizations that provide guidance to end users in the field to maximize adoption by those professional communities. These include rehabilitation professionals and those in physiatry, social work, rehabilitation psychology, nursing, and neuropsychology. The LEP group is comprised of persons with lived experience of TBI who have navigated VA, DOD, and civilian health care settings. The LEP perspective will be incorporated into all products developed to maximize acceptability by patients with TBI and their families. The PPP group is comprised of representatives from federal agencies (VA, DOD, NIDILRR, and MSKTC) to help align products with policies and vice versa.

#### Study Closure, Data Sharing, and Biorepository Plans

Data from this project will be made available to the TBI research community by depositing deidentified research data into a nationally available biorepository at the end of the study. Primary data collection will be from providers in the form of qualitative data collection, and the corresponding data will be made available. Qualitative data from our prior work funded by NIDILRR will be archived at the Inter-university Consortium for Political and Social Research Biorepositories. The TBIMS NDSC, which will serve as the WDMC for this study, already has procedures in place to submit its core data to national biorepositories (via previous DOD funding and ongoing NIDILRR funding). They will work with the ISC on qualitative data submission. Funds have been set aside in year 4 for this submission.

Once the data collection phase of individual studies has ended and the datasets have been cleaned and locked for analysis, the data will be deidentified, merged, and securely stored for all study analyses. The final deidentified merged datasets will be securely sent to the study contact MPI (RN-R). The final datasets and documentation will be stripped of identifiers prior to release for sharing to any participating study sites by request once the project has been completed. The datasets will reside with the study contact MPI. Data will be provided in a timely manner to those requesting information for research purposes once a data sharing agreement is in place that meets all the data sharing requirements of all participating sites. To fully protect the human participants, we will evaluate each data request to ensure that special circumstances do not exist that would permit anyone to deduce the identity of individuals from the data. In any such cases, we will share the data based on an agreement stating that (1) the data will be used solely for research, and no individuals will be identified in any manner; (2) the data will be secured by electronic safeguards; and (3) once data analysis is complete, the data will be returned or destroyed.

### Individual Project 1: Health Care Disparity Risk Reduction via an Innovative Medical Records Flag for Post-TBI Cognitive Impairment: Cognitive Nudge Decision Support Tool

#### Problem and Solution

##### Problem

TBI is a leading cause of long-term disability [25], affecting over 2.8 million individuals annually in the United States [26] and over 458,000 military personnel since the year 2000 [27]. TBI has been described as an *invisible disability* because it causes substantial cognitive impairments [28] without overt physical evidence of injury [29]. During the chronic recovery stage, 48%‐50% of V/SMs were found to need assistance with coordinating and accessing medical services [30,31]. The need for a health care proxy to help access and coordinate health services is likely influenced by the unmet need for cognitive assistance, as reported by 61%‐70% of V/SMs followed 1 to 5 years after TBI [30]. The cognitive functioning and supervision needs of persons with TBI are not stagnant and indeed fluctuate over time [32], which further complicates health care service delivery. Environmental factors, such as institutional policies and clinical provider attitudes, serve as barriers to addressing health care access and coordination needs [30]. Involvement of a health care proxy (often a family member) has been found to improve access to services [6] and health outcomes among persons with both cognitive impairment and TBI [33,34]. Family involvement during care delivery is espoused by specialized (typically inpatient) rehabilitation programs where neuropsychological assessment informs individually tailored care recommendations [35,36]. However, outside these settings, family or other health care proxies may be excluded from health care encounters when providers are unaware that TBI-related cognitive impairments limit the patient’s ability to independently engage, retain information, and adhere to treatments. Unfortunately, 37% of caregivers of persons with TBI reported that they were excluded from health care to the detriment of their loved ones. Cognitive specialists, such as neuropsychologists, often evaluate decision-making capacity and make recommendations about the need for health care proxy involvement [37], but these tailored recommendations are embedded in lengthy reports that may not transfer to other health care settings. Consequently, other providers are unaware of these needs, placing the burden of identifying and accommodating cognitive impairment on clinicians with limited time and resources.

##### Solution

The proposed project will develop an innovative electronic medical record (EMR) flag (ie, “cognitive nudge” to cue providers to include health care proxies during health encounters when recommended by TBI specialists. This cognitive nudge will be developed using a CBPR approach in partnership with key partners, including health care clinicians, administrators, and persons with lived TBI experience, and it will be pilot tested in a V/SM health care setting [38].

### Background

Cognitive morbidity is a high-priority challenge in V/SMs with chronic TBI. In a sample of 120 persons with chronic TBI and 78 family members, 77% reported cognitive difficulties (impaired memory, concentration, processing speed, task initiation and organization, and problem solving), and it was mentioned that there was a high-priority unmet need [39]. These study findings were mirrored in studies of V/SMs with chronic TBI followed 1 to 5 years after injury, with 59%‐61% endorsing ongoing memory difficulties and 47%‐58% endorsing ongoing problem-solving difficulties [30,31].

Cognitive morbidity is associated with worse health outcomes. Survivors of TBI are at increased risk for myriad health morbidities, including neuroendocrine, genitourinary, cardiovascular, cerebrovascular, neurological, and systemic metabolic conditions, with reduced life expectancy [40,41]. Up to 41% of persons with TBI are readmitted to the hospital within the first year after injury [3,42,43], and poorer cognitive functioning has been shown to predict rehospitalization [3,42]. Poorer cognition was associated with both health morbidity [44] and rehospitalization [3,42].

Cognitive morbidity is the leading barrier to engaging in health care for persons with TBI. Persons with cognitive impairment following TBI experience myriad barriers across multiple dimensions of health care access. In a recent qualitative study of TBI survivors and their caregivers, 27% experienced communication issues impacting health care delivery [6]. Studies of TBI survivors at 1, 2, and 5 years after TBI found that 48%‐50% needed assistance with coordinating and accessing medical services within the past year [30,31], with other studies identifying cognitive impairment as a barrier to health care access and optimal utilization [45]. Indeed, when persons with TBI have health care advocates, it is considered a facilitator to accessing needed care [46,47].

A VA physician made the following statement:

*TBI patients unfortunately forget things, they're not as compliant because they don’t remember… You have to be cognizant of that when treating TBI patients. It's the patient population. You give them five or ten things (to do), they're probably not gonna do any of them.*

Providers require mechanisms that alert them to the need for health care proxy involvement. Federal law (eg, the Health Insurance Portability and Accountability Act of 1996 [HIPAA] and the Privacy Rule), while well-intentioned to protect a care recipient’s privacy, can unfortunately create an environment that functions to exclude family from health care encounters even when exclusion would be detrimental to the patient. When care providers, such as primary care physicians, are made aware of a patient’s cognitive disability, they are better positioned to help ensure that the patient’s rights and preferences are prioritized in clinical decision-making and care planning [48]. Indeed, the inclusion of care partners significantly improved health outcomes [49]. Fortunately, designed EMR systems facilitate a process for alerting health care providers to specific needs, patients’ preferences, and clinician-recommended actions for optimizing patient care. Feasibility and clinical utility of EMR nudges (or flags) have been demonstrated in prior research (eg, risk of readmission and suicide) [50-52]. However, it is not yet known how best to implement an EMR nudge to alert health care providers of cognitively impaired persons with TBI who may benefit from a health care proxy during health care visits.

With regard to impact, in the short term, this study will create a blueprint to reduce health care inequity and improve care for cognitively impaired V/SMs with TBI, which is an “invisible” disability affecting 5% of Americans and causing life-altering challenges without overt signs of injury [53]. At the conclusion of this study, we will have developed and refined the toolkit required to implement a cognitive nudge intervention in partnership with key partners; these outputs will be disseminated broadly for immediate use. In the long term, this study has the potential to positively impact health care delivery for persons with cognitive impairments outside of TBI. As evidence-based chronic care management protocols are being developed for TBI, similar nudge interventions can facilitate the implementation of proactive surveillance and tailored disease management interventions.

### Objectives

The aims are as follows:

- Aim 1.1 (discover): We aim to engage health care providers, persons with TBI, and their families to identify content, processes, and procedures for developing a cognitive nudge clinical support tool.
  - Aim 1.1a: We aim to determine the best practices across professional communities for the identification of persons with TBI-related cognitive impairments and recommendations for the engagement of health care proxies in medical care. We will conduct online surveys (aim 1.1a1) and focus groups (aim 1.1a2) with professional groups to identify these practices.
  - Aim 1.1b: We aim to identify the tailored needs and operability of a cognitive nudge intervention from the perspective of persons with TBI and their families (LEPs). The ISC will conduct focus groups with persons having lived experience to inform the development of the cognitive nudge intervention.
- Aim 1.2 (define and develop): We aim to co-design a cognitive nudge clinical decision support tool with health care providers, persons with TBI, and their families.
  - Aim 1.2a: We aim to develop a toolkit of resources and an implementation plan for the cognitive nudge clinical decision support tool intervention. In collaboration with the ISC, we will triangulate data from aim 1.1 to inform product development.
  - Aim 1.2b: We aim to engage partners, including CEC partners and individual engagement partners (IEPs), in the formative evaluation of the cognitive nudge toolkit and implementation plan. The ISC will conduct semistructured interviews during regularly scheduled CEC meetings to obtain input on the format, content, and usability to refine the cognitive nudge.
- Aim 1.3 (validate): We aim to implement (pilot) and evaluate the outcome of the cognitive nudge at a major VA medical center and PRC.
  - Aim 1.3a: We will conduct a formative evaluation of the clinical nudge to preliminarily assess acceptability, relative advantage, usability, and complexity. Following a 4-month implementation pilot, we will conduct semistructured interviews with providers, administrators, and patient/caregiver dyads to evaluate implementation outcomes and perceptions of proxy engagement in health care encounters.
  - Aim 1.3b: We aim to disseminate the cognitive nudge toolkit and implementation plan with professional partners. We will refine the toolkit and implementation plan based on the pilot and disseminate it by leveraging professional partners from federal agencies and professional organizations.

### Design

This project falls within the preimplementation phase of the QUERI Implementation Roadmap. The goal is to improve access to health care by improving overall engagement of persons with TBI in their health care encounters. Our prior work has shown that caregivers and advocacy organizations are critical to engaging in health care encounters and overcoming communication barriers reported by veterans with lived TBI experience [6]. The proposed mixed method study will identify the best practices to inform cognitive nudge development while considering LEP community preferences (aim 1: discover), develop products for supporting cognitive nudge implementation (aim 2: develop), and conduct a pilot to refine and disseminate the intervention for future implementation and evaluation (aim 3: validate and disseminate).

### Aim 1.1: Discover (Study Months 7-24)

#### Participants

##### Participants for Aim 1.1a1 (Provider Cohorts)

We will partner with PPPs and IEPs to recruit a national sample of health care providers who treat persons with TBI. Each provider will be required to meet the following inclusion criteria: (1) being a licensed health care provider (eg, medical provider [physician]), a psychologist (rehabilitation or neuropsychology), or other allied health care professional, and (2) spending at least 20% of their professional time in clinical services for patients with moderate-to-severe TBI.

To collect a representative sample across provider types and health care systems, we intend to recruit a minimum of 350 survey responders. This sample size will be large enough to estimate means and proportions from the survey, with sufficiently narrow CIs. A sample size of 350 will estimate a 2-sided 95% CI on standardized means (SD=1) with a margin of error no larger than 0.101 [54] and a 2-sided 95% CI on proportions with a margin of error no larger than 0.107 [55,56].

Partnering with professional organizations specific to this study (National Academy of Neuropsychology), hospital administrators (Chief of Staff, and Chiefs of Medicine, Mental Health, and Rehabilitation), and overall CEC partners increases access to a large pool of professionals involved in the care of persons with TBI for survey purposes, maximizing the positive response rate. We plan to use the Dillman method, which has produced response rates up to 50% [57]. The study team has a successful track record of recruiting providers for surveys involving clinical practice [58].

##### Participants for Aim 1.1a2 (Provider Focus Groups)

We will conduct up to 5 focus groups (n=8 maximum/group) until thematic saturation is reached. Each provider will be required to meet the following inclusion criteria: (1) being a licensed health care provider (eg, medical provider [physician]) or psychologist (rehabilitation or neuropsychology), and (2) spending at least 20% of their professional time in clinical services for patients with moderate-to-severe TBI. We will partner with volunteers identified by the PPPs to recruit a diverse provider cohort. We will examine CFIR-guided constructs to determine the extent to which the cognitive nudge intervention is viewed as suitable or likely to be used, and how it can be successfully delivered.

Convenience and snowball sampling methods will be used to identify and recruit participants across the professional organizations engaged. We plan to overenroll underrepresented groups to understand the implementation challenges across racial and ethnic groups.

The number of focus groups has been determined for achieving theme saturation. Theme saturation is defined as the lack of new codes being introduced into codebook development [59]. For determining the number of focus groups needed in qualitative exploratory research, we have referred to the methodological work by Hennink et al [59]. The authors showed that 94% of all codes and 96% of high-prevalence codes are identified by the fourth focus group, with no new codes of this type introduced thereafter. They highlighted that this approach is particularly suitable for designing interventions (the purpose of this aim) but may be limited for other purposes [59]. Therefore, we propose that up to 5 focus groups with ≤8 persons per group is more than sufficient to achieve thematic saturation.

##### Participants for Aim 1.1b (Lived Experience Focus Groups)

We will conduct up to 5 focus groups (n=8 maximum/group) until thematic saturation is reached. The inclusion criteria for the focus groups are as follows: (1) having moderate-to-severe TBI or being a TBI proxy care partner, and (2) having a reliable internet connection (web-based focus group participation).

We will partner with our LEPs and use TBIMS patient registries to recruit lived experience participants following IRB procedures. This approach has worked well to recruit V/SMs with lived experience and their families in past qualitative TBI research [6].

The number of focus groups has been determined for achieving theme saturation. We have referred to the methodological work by Hennink et al [59], and we propose that up to 5 focus groups with ≤8 persons per group is more than sufficient to achieve thematic saturation.

### Data Collection

#### Data Collection Measures and Tools for Aim 1.1a1

A semistructured survey developed in collaboration with our CEC members (PEPs and LEPs) will be conducted with the goals to: (1) determine current practices that identify persons with cognitive impairments after TBI and policies that require it across health care settings; (2) determine current practices for identifying medical decision-making capacity or inability to participate in health care encounters independently; (3) identify documentation practices and locations in medical record systems for identifying previous goals (1 and 2); (4) review innovative practices for ensuring persons with TBI have health care proxy assistance in medical encounters (open-ended question); and (5) assess provider attitudes regarding satisfaction with existing practices and willingness to trial new approaches to ensure health care proxy assistance in medical encounters. Questions will be developed using both the open-ended (qualitative) approach and the Likert scale rating.

#### CFIR Guide for Aims 1.1a2 and 1.1b

The CFIR guide will be used to inform the implementation determinants (facilitators and barriers) of the cognitive nudge intervention to determine the extent to which the cognitive nudge intervention is viewed as suitable and likely to be used, and how it can be successfully delivered. The CFIR guide has been used internationally to study all types of health conditions in military and civilian health care settings [60,61]. The overarching access framework domains from the supply/organization side and consumer/demand side will also inform the focus group questions and have been successfully used in our own TBI research [6,46,47,58]. The focus group guide will be pilot tested with people representing those we will engage to ensure comprehension of questions and content.

### Study Procedures

#### Procedures for Aim 1.1a1

Recruitment flyers, emails, social media posts, and telephone scripts will be developed and approved during study initiation. Project 1 team members and PPPs will hold monthly meetings during quarters 3‐4 of year 1 to develop and field test the survey, which will be deployed (quarter 1 of year 2) to members of professional health care organizations (eg, American Congress of Rehabilitation Medicine, Association of American Physicians, National Academy of Neuropsychology, American Psychological Association’s Division of Rehabilitation Psychology, VA, and DOD) via organizational email distribution groups and personal network emails of the research team and core groups. Survey links will be sent up to three times via email. Up to 20 respondents will be randomly selected to receive a US $50 gift card for participation.

#### Procedures for Aims 1.1a2 and 1.1b

Recruitment flyers, emails, social media posts, and telephone scripts will be developed and approved during study initiation. Up to 10 focus groups (5 focus groups per stakeholder type [professionals and persons with lived TBI experience] will be conducted, with ≤8 people for each focus group). Each focus group will be scheduled for 90 minutes, conducted on online conferencing platforms (eg, Microsoft Teams and Zoom), digitally recorded, and professionally transcribed with quality checks by the study team. Participants will receive a US $50 gift card for participation if permissible.

### Analysis Plan

The project team, in consultation with partners (PPPs, LEPs, and IEPs), will analyze the collected data.

#### Survey Analysis for Aim 1.1a1

Survey data will be summarized descriptively using means, SDs, and percentiles for continuous data, and frequency counts and proportions for categorical data. Survey data will be summarized for the overall sample and by provider type and health care setting. Responses will be compared across provider types and health care systems using chi-square tests, ANOVA, and nonparametric alternatives as necessary (Fisher exact test and Wilcoxon rank sum test). Open-ended survey responses will be exported to an Excel file. Individual responses will be analyzed using a rapid matrix analysis approach by comparing similar responses and grouping them to create categories [62]. For both the qualitative and quantitative data, sensitivity analyses will examine responses by demographics (age, gender, race, ethnicity, and years in practice).

#### Qualitative Analysis for Aims 1.1a2 and 1.1b

Focus group and individual interview transcripts will be analyzed using matrix analysis, a rapid assessment approach, to summarize and identify themes. Rapid assessment is a team-based approach to iteratively collect and analyze qualitative data that emphasizes speed of data collection and analysis in relation to focused programmatic questions or problems [62]. Two experienced qualitative researchers will conduct the matrix analysis. Together, they will create codes both deductively from known constructs and inductively from the data, and use matrices to categorize responses under domain names, applying codes that emerge from the data. Codes and preliminary themes will be reviewed by the research team and partners to establish consensus. A matrix analysis approach will facilitate a rapid turnaround of the results to provide immediate feedback to inform the cognitive nudge and supportive implementation materials, such as the implementation blueprint.

### Aim 1.2: Design the Toolkit and Develop the Implementation Plan (Study Months 25-37)

#### Overview

Using a CBPR approach that strengthens knowledge translation efforts [63,64], we will schedule a series of meetings to review the triangulation of aim 1 findings with the CEC, identify needs informing toolkit product development (Multimedia Appendix 8), and evaluate the usability of toolkit content.

#### Initial Content Identification

Project 1 engagement partners represent key partners in the eventual implementation of the cognitive nudge decision support tool. They will review aim 1 findings to identify needs from their unique perspectives and make suggestions for the content, structure, and language used in products in the toolkit.

#### Product Development and Prioritization

Project 1 IEPs (VA hospital administration and national VA physical medicine and rehabilitation policy leads) will review product suggestions and prioritize content development. Existing national policies for the use of nudge interventions and products that exist for other clinical reminders in the EMR will be used as a starting point for all products, such that most products will be adaptations to maximize feasibility and acceptability, and to optimize integration into the existing health care system. The study team will then utilize feedback across engagement partner communities to adapt existing content and develop new content for the toolkit.

#### Formative Evaluation of Toolkit Products and Implementation Plan

The ISC will conduct semistructured interviews with the CEC and IEPs for user testing of toolkit products. No formal analyses will be conducted, and feedback will be used by project investigators to finalize the content.

### Aim 1.3: Deliver and Validate (Study Months 38-48)

#### Overview

We will work with project 1 team members (hospital administration and national physical medicine and rehabilitation EMR leads) to pilot the implementation of the cognitive nudge decision support tool for final refinement prior to dissemination.

#### EMR Implementation

The study team will follow recommendations for implementing a category 2 patient record flag (ie, nudge) in compliance with Veterans Health Administration Directive 2010‐05 and Patient Record Software Patch v5.3 (March 2019). These directives outline policy and guidance for the proper use of patient record flags to enhance safety for patients, employees, and visitors. Category 2 flags are for nonimmediate risk that can be implemented at a facility level. Category 2 flags denote risk factors (eg, cognitive impairment) that must be known in the initial moments of a patient encounter. Facility-level partners responsible for the implementation of a category 2 flag are involved as IEPs in this proposal. Software patches in the VA EMR system permit entry, editing, and monitoring of patient flags. The team and IEPs will work with the Tampa VA Patient Safety Office, overseeing EMR flags to trial the cognitive nudge intervention.

#### Staff Training and Pilot Implementation

Veterans Health Administration Directive 2010‐05 requires training on any element of software utility, activation, continuation, and deactivation of the cognitive nudge. Additional areas of training include confidentiality and appropriate responses to a cognitive nudge. Training in these areas will follow the toolkit and implementation plan developed in aim 2. Training for Tampa VA TBI rehabilitation providers and primary consultants will be delivered via existing staff meetings across services. As noted, chiefs of those services serve as project I engagement partners to facilitate local training and unique needs across services. Following training, the cognitive nudge will be piloted for a 4-month period in the inpatient polytrauma rehabilitation unit (18/56 inpatient beds) at the Tampa VA, the largest rehabilitation facility in the VA Healthcare System.

#### Formative Evaluation of the Implementation of the Cognitive Nudge Clinical Decision Support Tool

At the conclusion of the pilot, ISC staff will conduct semistructured interviews with providers who interacted with the cognitive nudge during the pilot (Medical Director of the brain injury inpatient program and 2 unit neuropsychologists) using key informant interviews. The ISC will also conduct focus groups with allied health providers (case management providers, rehabilitation therapists, nurses, and family therapists). Key informant interviews and focus groups will use the CFIR Interview Guide to identify facilitators and barriers to implementation. Codebook development will include key implementation constructs of acceptability, relative advantage, usability, and complexity. Data will inform final revisions to the toolkit and the implementation plan tailored to rehabilitation settings in the VA/DOD system. Persons with TBI receiving care during the pilot and their health care proxies will also be approached and consented to complete a semistructured interview with the ISC to understand whether the cognitive flag nudge intervention enhanced health care proxy engagement in the health care encounters during the pilot. Findings will be integrated into the finalization of the toolkit.

### Potential Challenges and Solutions

A potential problem is insufficient interest in surveys or focus groups. If we experience difficulty recruiting representative samples, we will inform individual members of the CEC to personally invite their respective networks to participate in surveys and/or focus groups. We will use feedback from participants to optimize the research participation experience and reduce deterrents to participation as applicable. Using methods proposed herein, our team recently recruited over 350 professionals to complete an internet survey in a 4-month time span. By building on our past success, we will minimize recruitment barriers.

Another potential problem is low acceptability of the cognitive nudge among persons with lived experience. We have considered the potential disadvantages of an EMR flag alerting providers to a patient’s cognitive challenges, and we will work with our partners to determine the best practices for deferring and/or removing the flag for any reason, including patient/family preferences. A better understanding of what aspects of a nudge intervention may be less attractive to those with lived experience is essential for designing the toolkit proposed herein. Based on extensive data from our team and others, a plurality of TBI survivors living with cognitive impairments report that these impairments pose barriers to care, and this study will determine how best to mitigate those barriers through co-development of a decision support tool in partnership with partners.

### Community-Based Participatory Approach

This study embodies the CBPR approach by engaging end users of the cognitive flag intervention as full partners in the conceptual development, development, and dissemination of products developed across study aims.

### Individual Project 2: Provider Toolkit for Accommodating Cognitively Impaired Persons in Evidence-Based Treatments

#### Problem and Solution

##### Problem

Proposal investigators have robustly shown that cognitive morbidity (ie, cognitive impairment) is the number one rehabilitation need in the chronic stage of TBI [7,30,31], with unmet needs associated with poorer satisfaction with life among V/SMs [7]. Further, V/SMs with greater TBI disability (and greater cognitive morbidity) experience unique barriers to accessing care in the chronic stage of TBI [6]. Our most recent work characterizing health care delivery for the most common comorbid conditions [8,65] in TBI highlights cognitive morbidity [46,47] as a barrier in referral to and receipt of evidence-based behavioral treatments (EBTs), limiting health care access and meaningful engagement in the management of TBI comorbidities that may improve outcomes (ie, suicide risk, PTSD severity, arrest rates, and worsening cognition) [66-70].

##### Solution

The proposed project will address determinants (ie, facilitators and barriers) that limit a cognitively impaired person’s ability to engage in EBTs for the most common comorbid conditions (ie, PTSD, depression, sleep disorders, and chronic pain) in TBI by conducting an environmental scan to identify existing EBT adaptations for persons with cognitive impairments (aim 1), selecting adaptations identified through a CBPR approach (aim 2), and developing and disseminating a provider toolkit for accommodating cognitively impaired persons, which will aid the identification and engagement of cognitively impaired persons in EBTs (aim 3).

### Background

Psychological health conditions are common in the chronic stage of TBI. Our group has found high rates of sleep difficulties [71-73], chronic pain [8,74], PTSD [67], and depression [75] in individuals with TBI from months to many years after injury. As high as 46% of those with moderate to severe TBI report ongoing chronic pain from 1 to 30 years after injury. These conditions often co-occur, resulting in constellations of disorders, with signature injuries of military service and deployment making up the polytrauma clinical triad (ie, TBI, chronic pain, and PTSD).

Post-TBI psychological health conditions are associated with worse recovery and an increased risk of suicide. Chronic pain, depression, and PTSD are associated with greater disability and reduced quality of life following TBI [76-79]. Unaddressed, these conditions may exacerbate one another. For instance, the presence of PTSD contributes to avoidance of activity, exacerbating functional limitations [80,81] and resulting in poorer adjustment to ongoing pain [82]. Alone or in combination, the presence of these psychological health conditions is associated with an increased risk of suicide [83,84], making successful engagement in evidence-based treatment in this population an urgent need.

A VA neuropsychologist made the following statement:

*It is challenging to get comorbidities treated by providers who understand cognitive disability that come along with brain injury…finding a therapist who can take into consideration the cognitive limitations is hard to find.*

Cognitive difficulties have been found to limit referral to and receipt of evidence-based treatments for behavioral health needs in persons with TBI [47]. In our recent work, 67% of providers described cognitive impairment as the number one barrier to receiving evidence-based health care [47]. Provider expectations of limited treatment benefit due to cognitive deficits limit referrals to evidence-based care, which can be adapted to overcome cognitive impairments. In 2022, the American Psychological Association published a new guideline mandating disability accommodations [85]; however, a major gap was the lack of specificity for what accommodations would be enacted and how to integrate them into behavioral health treatments. In the peer-reviewed literature, existing strategies for adapted behavioral health treatments for disabilities, such as cognitive impairments, are inconsistent and lack specific guidance for providers [86-88] delivering EBTs. To address this gap, this project proposes to develop a toolkit of specific accommodation strategies, reviews of the literature, and specific guidelines for managing cognitive limitations in delivering evidence-based behavioral health care. The toolkit will contain practical strategies consolidated from practice-based surveys, evidence synthesis, and validation by clinicians and persons with lived experience of TBI.

Project investigators [89] have shown that adaptations for TBI morbidity are effective in the delivery of evidence-based, behavioral health treatments for veterans with TBI. The MPI JNH has adapted cognitive behavioral therapy for chronic pain in veterans with TBI and shown improvements in pain interference, a key outcome in this population [89]. Other project investigators have used these adaptations in collaborative care models and the delivery of motivational interviewing to promote the use of continuous positive airway pressure among veterans with TBI and cognitive morbidity. Further, frontline behavioral treatments for PTSD (cognitive processing therapy and prolonged exposure therapy) [90-93] and insomnia [94,95] have also been adapted for persons with TBI and shown to have efficacy. Investigator-described adaptations include the use of concrete examples (avoid abstract constructs), the use of visual aids, and caregiver engagement in sessions. Not all treatments manualized the adaptations but included abbreviated sessions, use of reminder calls, and integration of memory aids [90-93]. To facilitate the utilization of cognitive adaptations of behavioral health treatments for persons with TBI, we propose to use HCD to gather existing resources for adapting treatments for cognitive difficulties, curate those resources to identify the most up-to-date and relevant resources with the support of expert provider partners, develop a toolkit, and conduct a formative review to determine readiness for dissemination. Partnering with the CEC, we will create awareness of the toolkit of adaptation strategies to ultimately increase access to evidence-based treatments for common TBI comorbid conditions known to worsen outcomes.

### Objectives

**Figure 4.**
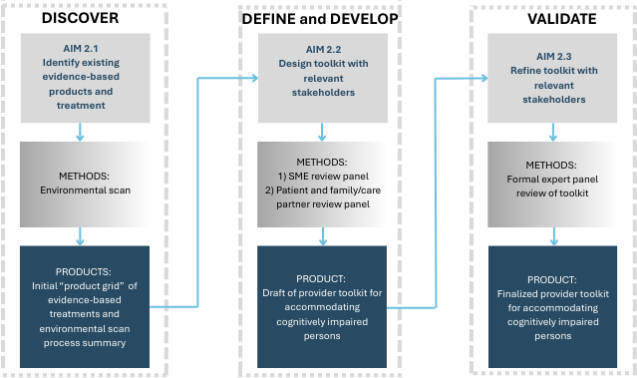
Project 2 aims. SME: subject matter expert.

The aims of project 2 are presented in Figure 4. The aims are as follows:

- Aim 2.1 (discover): We aim to conduct an environmental scan of existing recommendations and EBT adaptations for persons with cognitive impairments.
  - Aim 2.1a: We aim to conduct an environmental scan to identify existing resources that fall into three broad categories: (1) tools for assessing cognitive difficulties; (2) general information on adaptations for EBTs, including how to include caregivers/family members when needed; and (3) manuals that include adaptations of existing evidence-based treatments.
  - Aim 2.1b: We aim to summarize the environmental scan results to develop a product grid.
- Aim 2.2 (define and develop): We aim to engage the CEC in prototyping the provider toolkit for accommodating cognitively impaired persons by defining its scope, elements, and function.
  - Aim 2.2a: We aim to convene a SME review panel and include LEPs to (1) select which high-quality materials from the product grid should be included in the toolkit and (2) brainstorm additional toolkit content, layouts, and functions.
  - Aim 2.2b: We aim to synthesize data from aim 2.2a and develop a prototype of the toolkit.
- Aim 2.3 (validate): We aim to activate the CEC to conduct a formal review of the provider toolkit prototype for accommodating cognitively impaired persons to validate its design and identify implementation strategies.
  - Aim 2.3a: We aim to conduct a formal review of the toolkit prototype to determine key strategies for dissemination to practitioners, educational institutions, health care systems, and other identified groups to increase the utilization of the recommendations.
  - Aim 2.3b: We aim to synthesize formal review data and make data-driven revisions to finalize the toolkit.

### Design

The proposal aligns with the preimplementation phase of the QUERI Roadmap through the development of knowledge products (toolkit) from published literature and other sources. An HCD process will guide the design and implementation of the toolkit. HCD is an approach that “centers” end users (ie, the people who will ultimately use or benefit from a product or service) by integrating them into every aspect of the design process [14,15,17], thus strengthening the impact and utilization of the product or service. HCD can combine familiar qualitative and quantitative data collection methods with novel efforts to engage beneficiaries in an iterative design process. Each of our project aims aligns with the 4-phase Double Diamond HCD model [16]. This model directs researchers and designers to engage end users to first *discover* and *define* a problem and then take focused action to *develop* and *deliver* (ie, *validate*) a solution [16]. HCD supports our process of making high-quality evidence-based tools that are as effortless as possible to find, easy to understand, and esthetically pleasing.

### Aim 2.1: Environmental Scan and Product Grid Development (Study Months 1-18)

#### Initial Toolkit Content Identification (Aim 2.1a)

Following project approval as a QI project, the team will commence a comprehensive environmental scan using a CBPR approach. First, the team will review project goals with CEC members to facilitate identification of provider resources for modifying behavioral health treatments for persons with cognitive impairment. Professional partners will be asked to help identify existing online materials and utilize their professional contacts for finding additional materials and tools. Next, an anonymous Qualtrics survey that queries providers about resources used to provide adaptations for persons with cognitive impairment will be developed. The survey will include a request to submit content to the project (if permitted) to complete the environmental scan. The PPPs within the CEC will be asked to help disseminate the survey to their constituency using professional and email listserv platforms available within their organizations. Social media content will be developed by our social media specialist and shared to help increase the reach of the survey. The WDMC will develop an online platform to store and organize relevant materials about adaptations.

#### Product Grid Compilation (Aim 2.1b)

Research staff guided by MPIs (AMM, JMH, and Daniel Goldschmidt) will work with the ISC to conduct the comprehensive environmental scan process by (1) compiling collected materials from SMEs on a secure drive, (2) searching extensively via compiled sources (eg, organization websites) and web searches for additional leads on tools, and (3) conducting a literature review with search terms to capture adaptations for cognitive deficits for behavioral health treatments (eg, chronic pain, PTSD, and insomnia). A study research associate will begin this process in quarter 1 of year 1, as they will already be onboarded at the start of the project. Materials identified through the environmental scan will be entered into an Excel document using a product grid template. The product grid, developed by the MPI JNH, uses rows and columns to review, organize, and rate materials (1 product per row) by preestablished qualities or domains (column descriptors). Research staff will review each resource and preliminarily complete a review of products in the product grid. SMEs will serve as IEPs for project 2 to aid in product grid validation. IEPs represent content expertise across commonly comorbid conditions in military personnel after TBI (Multimedia Appendix 9). IEPs will partner with the project team to validate adaptations proposed and their impacts on specific evidence-based treatment content.

### Aim 2.2: Expert Panel Product Grid Review (Study Months 19-36)

#### Review and Rating of Products by SMEs (Aim 2.2a)

Once the product grid is populated based on a preliminary review by the study team, a secondary SME review will be conducted. The product grid will include general accommodations for cognitive difficulties and be organized by psychological health condition (ie, PTSD, depression, chronic pain, and sleep disorders). SME review panel members will be assigned products corresponding to their primary area of expertise to review. All panel members will review general accommodations. Each line item in the product grid will be evaluated by 2 SMEs, and for any product where there is a discrepancy in ratings, a third SME will evaluate to help reach consensus. Products will be assigned to and independently reviewed by the SME review panel along the following domains: (1) verification of the intended audience (eg, licensed therapist), (2) quality of accommodations made (rating appropriateness from 0 [poor] to 5 [excellent]), and (3) complexity of using the tool (0 [very difficult] to 2 [very easy to implement]).

#### Convening of the SME Review Panel

Once the SMEs complete their individual reviews of the product grid, the SME review panel will convene to review the products identified as being of high quality, which can be implemented with the greatest ease. Prioritized products will be translated into tools or components of the toolkit. SMEs will collaborate to (1) identify gaps in tools that may require development and (2) provide guidance on how to create tools that need to be developed.

#### LEP Engagement

In tandem with the SME review, the LEPs will convene to review the identified products to be included in the toolkit to inform appropriateness for the target patient population. Additionally, the LEPs will discuss the appropriateness of language use and orientation for providers when working with persons with TBI. The LEPs will be specifically asked about the appropriateness of language regarding the introduction of tools and the overall approach to guiding providers/end users of the toolkit on integrating modifications. Gaps in the toolkit will be identified through discussions with LEPs based on lived experiences of engaging with behavioral health providers and treatments. For example, feedback about the importance of having a toolkit like this or the frustrations experienced when interacting with behavioral health providers as a person with TBI will be invaluable to the project team for creating content in the “introduction” and “purpose of the toolkit” sections.

#### Toolkit Compilation, Additional Content Creation, and Design (Aim 2.2b)

The project team will triangulate feedback from aim 2.1a to develop the initial prototype of the toolkit, tentatively titled “The Provider Toolkit for Accommodating Cognitively Impaired Persons.” The team will partner with the ISC product designer to compile tools in the product grid and develop additional tools or toolkit components (see Table 3 for possible components of the toolkit). Details outlined in the product grid categories will inform background narratives, applications, and other framing aspects for each tool.

**Table 3.** Sample toolkit content.

Toolkit section	Information
Executive summary	Overview and purpose of the toolkit (gap addressed, applications, and target audience)
Table of contents	Identified sections
Tip sheets	Abbreviated guidance for implementation, including engagement of health care proxies in the treatment process, summarized in an algorithmic approach
Quick references	Source materials, including literature reviews, existing treatment manuals, clinical practice guidelines, and position statements, for relevant topics
Screening resources	Resources for identifying those in need of cognitive adaptations in treatment processes
Tools: introduction and framing of each tool	Target population for each tool, brief background of creation, and utility for specific treatments and disorders, including summary infographics to facilitate understanding
Patient and family materials	Tip sheet explaining behavioral health approaches for persons with cognitive morbidity
Training	PowerPoints, fact sheets, videos, etc
Resources	URLs and references
Toolkit development background	Literature review of the topic (to enhance credibility of the toolkit)
	Project leadership and history
How to share the toolkit	Instructions on how to share toolkit content

### Aim 2.3: Engagement of SMEs (Study Months 37-48)

#### Activate the CEC to Conduct a Formal Review of the Toolkit Prototype (Aim 2.3a)

A formative review will be conducted to review, provide overall impressions, and make recommendations to improve the toolkit [96]. The toolkit prototype completed in aim 2.2b will be circulated several weeks prior to review sessions. Review sessions will take place online, and a facilitator and notetaker will be present. The team will engage the PPPs of the CEC to address organizational insight and high-order messaging, framing, and usability of the tools, as well as identify targets for future dissemination within organizations, educational institutions, and health care systems. The provider SME review panel will assist in validating the toolkit in terms of content validity, treatment fidelity, and usability of the tools across and within EBT approaches. The team will engage the LEPs of the CEC to provide final validation of the appropriateness and sensitivity of the language to guide providers/end users on integrating modifications in a way acceptable to patients and families. Any items still needing resolution will also be reviewed.

Data will be analyzed using the rapid assessment process [62], a team-based approach, which allows for quick turnaround of qualitative data analysis and provides prioritized findings. Review session notes alongside spot checks of audio recordings to clarify any missed notes or important reviewer quotes will be processed and cleaned with quick turnaround (within 3 days of data collection). This data analysis approach uses triangulation and iterative data analysis to quickly generate insights from qualitative data, and intensive team interaction to analyze the data will help to quickly translate reviewer feedback into prioritized modifications for the team to address in aim 2.3b [97].

#### Synthesizing Formative Review for Toolkit Finalization (Aim 2.3b)

The study team will integrate findings from the formative review to finalize toolkit compilation. The team will work with the social media specialist to create awareness of the project, and milestone completion will be highlighted on social media. The team will work with the WDMC to highlight project milestone completion on the overall FPA online dashboard. The study team will publish data collected across study aims in rehabilitation journals specializing in psychological health and as advised by the PPP members (eg, *Rehabilitation Psychology*, *Archives of Physical Medicine and Rehabilitation*, and *Provider Informational Education Page*). The toolkit and implementation plan (playbook) will be published on the study Toolshed Site hosted by the WDMC. The team will work with professional engagement partners and individual professional engagement partners to create a communication plan for the toolkit with various professional organizations, including those issuing clinical guidance to the field. Future work will evaluate the dissemination and implementation of the toolkit, and the outcome of toolkit use to benefit V/SMs with TBI.

### Potential Challenges and Solutions

A potential challenge is the absence of respondents for the survey of the environmental scan. Team members have had success recruiting from professional organizations using informal methods. Formal engagement and buy-in from these organizations from the start of the project will help facilitate commitment to engaging their respective constituencies for input.

Another potential challenge involves the scaling of the project and its timely completion. The project team will prioritize content for high-frequency conditions that are often comorbid with TBI and are known to be associated with poor outcomes.

### Community-Based Participatory Approach

The proposed project will use a CBPR approach to engage partners across all aims. The proposed aims will engage SMEs and LEPs in aim 1 to review the product grid, aim 2 to develop toolkit content, and aim 3 to conduct a formative review of the finished products in the toolkit. Collectively, our CBPR approach will ensure that the toolkit and its contents are relevant, useful, and appropriate for the target end users.

### Individual Project 3: Empowering Teams to Implement Evidence-Based Behavioral Interventions

#### Problem and Solution

##### Problem

Challenging behaviors after moderate-to-severe TBI (eg, agitation and impulsivity) are common (44%‐74%), frequently impacting access and quality of health care [98]. Our preliminary data indicate that these challenging behaviors are associated with denial of access to health care settings for persons with TBI, as well as increased rehabilitation staff injuries and turnover [58,99]. Possibly due to perceived lack of alternative options, one-third of providers worldwide have reported treating challenging behaviors with sedating medications thought to impede adaptive behavior, cognition, and neurorecovery [100,101]. While effective methods for optimizing adaptive behavior while reducing challenging behaviors exist, widespread implementation of evidence-based, nonpharmacological treatment programs has not yet occurred.

For the question “What are the challenges to have effective programs that manage maladaptive behaviors?” an inpatient rehabilitation provider made the following statement:

*Limited knowledge of the nursing staff regarding TBI, anticipated behavioral issues, and appropriate management strategies.*

##### Solution

We will develop and pilot a playbook for implementing an interdisciplinary program in TBI inpatient rehabilitation settings to promote adaptive behavior and reduce the challenging behavior of patients with TBI. The Staff Training in Assisted Living Residences-VA (STAR-VA) [102] will be adapted to produce the new program, TeamBI (Team-based Behavioral Interventions). This program will incorporate the principles of applied behavior analysis and positive behavior support [103,104] to prepare interdisciplinary teams in inpatient rehabilitation settings for setting up the environment to optimize adaptive, positive behaviors and minimize challenging behaviors. The playbook will also provide implementation strategies to assist with establishing the program.

### Background

Maladaptive behaviors are prevalent following brain injury. Behavioral disturbances after brain injury occur frequently and often involve the presence of socially unwanted behaviors (eg, agitation, impulsivity, and apathy). The frequency of these behaviors ranges from 44% to 74%, depending on injury severity and the time since injury [98]. A systematic review by Stéfan et al [98] in 2016 examined the prevalence of behavioral disorders following TBI. The most common behavioral disorders following brain injury include agitation (11%‐70%), aggression (25%‐39%), irritability (29%‐71%), and apathy (20%‐71%). Other common behavioral disorders seen following brain injury include impulsivity, confusion, disinhibition, fixation, confabulation, restlessness, sexual inappropriateness, and anosognosia (poor insight/awareness) [105-107].

Maladaptive behaviors negatively impact patient access to health care and long-term outcomes. Brain injury–related challenging behaviors have consistently been associated with disparities in health care access and poor long-term outcomes [58,108-110]. Behavioral disturbances often negatively impact an individual’s ability to fully engage in rehabilitation care, thereby limiting the scope and benefit of rehabilitation [111]. Further, behavioral sequelae can compromise discharge placement as many facilities and families are unable to manage challenging behaviors due to safety concerns and the need for extensive direct supervision [100,112,113]. Behavioral disturbances following brain injury significantly limit and may even prevent successful community reintegration, including returning to work and/or social environments [109,114].

Effective interventions exist for reducing challenging behaviors but are not consistently implemented. Clinical guidelines support the use of patient- and family-centered, integrated approaches to manage behaviors that interfere with participation in rehabilitation and with the successful performance of daily functions. Recommended interventions are based on two related schools of thought: “applied behavior analysis” and “positive behavior intervention and support.” Systematic reviews indicate that these approaches can be highly effective across a range of populations, including persons with brain injury [103,115-118]. The personalized nature of the interventions limits studies to single-case experimental designs that have inherent methodological challenges, especially when implemented in clinical settings. However, decades of research and clinical applications clearly support their use. The “ABC” model is fundamental to applied behavior analysis, as it examines and identifies the antecedents (what was occurring before the target behavior), behavior (accurately identifying the behavior to be modified), and consequences (what was the result of the behavior). Utilization of this model aims to identify behavior patterns and factors that may be eliciting and reinforcing unwanted behaviors. The positive behavior intervention and support model focuses on antecedents and on the notion that, through collaboration between staff, patients, and family members, the environment can be restructured to promote adaptive behaviors and, by extension, reduce challenging behaviors.

Unfortunately, the consistent application of behavioral interventions, even in units designated to treat persons with brain injury and challenging behaviors, is low [119]. Implementation of behavioral interventions requires staff with behavioral training, a cohesive team approach, and extensive knowledge about brain injury sequelae. In an international survey, only half of clinicians felt that they had adequate training to implement behavioral interventions [100]. In environments where staff rotate, training opportunities and time are limited, and behavioral expertise may not be available, more restrictive measures to manage unwanted behaviors may be used, such as sedating medications [100,120]. Some medications paradoxically increase challenging behaviors [101] and negatively impact cognition and neurorecovery [121,122].

For the question, “What are successful approaches to managing behavioral dysregulation during inpatient rehabilitation?” an inpatient rehabilitation provider made the following statement:

*Interdisciplinary approaches to behavioral management, with high team involvement, high family involvement, and collaboration across disciplines.*

Preliminary data support the need for systematic implementation of a comprehensive, interdisciplinary behavior intervention program. Our preliminary data gathered from a national survey of over 300 brain injury treatment providers revealed that the most frequently endorsed patient outcomes impacted by maladaptive behaviors included limited options for discharge placement (91%), poor therapy engagement (90%), interference with activities of daily living care delivery (86%), and increased length of stay (85%). The most frequently endorsed system outcomes impacted were an increased number of staff needed (85%) and increased staff burnout (81%) [58,99]. From the health care infrastructure standpoint, facilitators for managing behavior included a culture that prioritized staff collaboration and communication (69%) and specialized staff treating individuals with brain injury (61%). The identified barriers for managing maladaptive behavior included inconsistent treatment strategies (35%) and an ill-prepared, untrained workforce (65%). Some of the most important treatment elements for managing maladaptive behaviors included the use of environmental strategies (68%), the use of individualized care plans (67%), and the implementation of objective measures to monitor progress (35%).

Additional preliminary data collected from 28 PRC staff indicated that they lacked confidence in managing challenging behaviors (77% of team members requested additional training). In another preliminary study with a team at a civilian site, an educational program improved the confidence of rehabilitation staff in addressing behavioral disturbances [123]. These preliminary results highlight key facilitators and barriers that impact access to effective care. The findings provide intervention targets to inform treatment planning, program development, and educational programming.

The STAR-VA program addressed a similar gap in managing the challenging behaviors of persons with dementia receiving care in VA facilities. The STAR-VA program was developed at the request of the VA National Mental Health Director for Psychotherapy and Psychogeriatrics to improve the management of challenging dementia-related behaviors in VA Community Living Centers [102]. The STAR-VA intervention is an interdisciplinary behavioral approach based on the intervention by Teri et al [124], which was developed for training non-VA care workers to improve the care of older adults with dementia. Since the national rollout of STAR-VA over 10 years ago, empirical studies have consistently demonstrated that veterans enrolled in STAR-VA experience significant decreases in the frequency and severity of target behaviors, depression, anxiety, and agitation, with improved staff confidence [125,126].

STAR-VA is a collaborative, interdisciplinary team intervention based on universal behavioral management principles that are applicable to any patient population. The core elements include: (1) setting realistic expectations; (2) effective communication; and (3) identifying antecedents, behaviors, and consequences (ABCs) in order to modify the severity and frequency of challenging behaviors. While these elements are applicable to any patient population, additional elements specific to each target population are needed. As a result, STAR-VA training has not been authorized for other VA programs/providers, despite requests throughout the VA system of care and the need for standardized interventions to manage behaviors in many other patient populations. The goal of this study is to adapt the STAR-VA program for use with rehabilitation teams that care for patients with TBI. The STAR-VA program has consistently demonstrated efficacy in improving staff response to challenging behaviors, and we anticipate similar findings when it is adapted for teams treating veterans with brain injury.

We intend to adapt the STAR-VA manual to target brain injury and related behaviors. We will build upon the concepts presented in the manual to highlight how the social and physical environment can be set up to optimize adaptive behaviors following TBI and how behavioral interventions can reduce the incidence of challenging behaviors. In addition to staff training, the contents of the manual will guide staff in how to acquaint themselves with and train family members so that they can maintain the adaptive behaviors when the individual transitions home. We will also provide guidance and strategies on how to set up and sustain the program. We will be transforming a staff training manual developed for the dementia population into a playbook that will provide not only materials to train staff but also program implementation guidance to secure a more lasting and impactful change in TBI rehabilitation programs.

### Objectives

The aims are as follows:

- Aim 3.1 (discover, define, and develop): We aim to co-design a TeamBI Playbook through engagement of rehabilitation team members, persons with brain injury, and family members/care partners to identify areas of the STAR-VA manual that need adaptation and gap areas that need further development.
  - Aim 3.1a: We aim to engage persons with brain injury and family members/care partners to understand participants’ lived experiences with participating in rehabilitation and their perspectives regarding acceptable practices for optimizing adaptive behavior and reducing challenging behavior. We will conduct virtual focus groups with veterans having brain injury and their family members/care partners who have experienced rehabilitation.
  - Aim 3.1b: We aim to engage representatives from rehabilitation disciplines to identify the modifications and additions needed to transform the STAR-VA manual into the TeamBI Playbook. We will conduct 12 working meetings with IEPs to adapt the STAR-VA manual and integrate insights from focus groups (aim 3.1a).
  - Aim 3.1c: We aim to produce the TeamBI Playbook. Utilizing the data gathered in aims 3.1a and 3.1b, the investigators and ISC will compile and package the content of the playbook into an accessible online format.
- Aim 3.2 (validate): We aim to validate, refine, and disseminate the playbook. An initial validation study will be conducted through a pilot to determine the impact on rehabilitation team leaders’ self-evaluation of capability, opportunity, and motivation to implement the TeamBI program [126]. We will also determine if additional refinement is needed to address barriers to implementation.
  - Aim 3.2a: We hypothesize that exposure to the TeamBI Playbook will change rehabilitation leaders’ self-evaluation with regard to their (1) capability to implement a team-based program to reduce challenging behaviors and improve adaptive behaviors, (2) opportunity to implement a team-based program to reduce challenging behaviors and improve adaptive behaviors, and (3) motivation to implement a team-based program to reduce challenging behaviors and improve adaptive behaviors. A sample of rehabilitation leaders will complete a baseline assessment of capability, opportunity, and motivation to implement a team-based behavioral intervention program. They will then be exposed to the playbook and undergo reassessment. Pre- and postexposure responses will be compared to evaluate the impact of the playbook.
  - Aim 3.2b: We aim to determine if the playbook’s implementation strategies sufficiently address potential barriers. The rehabilitation leaders will complete an implementation readiness survey, and responses will be used to identify barriers that were not sufficiently addressed in the playbook.
  - Aim 3.2c: We aim to refine, finalize, and disseminate the playbook. We will refine the playbook based on findings from aims 3.2a and 3.2b. Following final review by the CEC, the playbook will be disseminated through the project toolshed, leveraging partners from the VA, other federal agencies, and professional organizations.

### Design

This project falls within the preimplementation phase of the QUERI Implementation Roadmap. The goal is to improve access to rehabilitation programs that effectively promote adaptive behaviors and reduce challenging behaviors, thus allowing patients with TBI to fully participate in rehabilitation. The proposed mixed methods study will engage veterans with TBI, family members/care partners, and rehabilitation professionals to develop and design the TeamBI Playbook (aim 1) and conduct a pilot to validate, refine, and disseminate the intervention for future implementation and evaluation (aim 2).

### Aim 3.1 (Study Months 3-24)

#### Participants

The participants in the focus groups will include veterans with brain injury and their family members/care partners. The inclusion criteria are as follows: (1) age 18 or older, (2) participants with TBI who have received rehabilitation and family members/care partners of veterans with TBI who have received rehabilitation within the past 5 years, and (3) online access to participate in a virtual meeting.

We will be conducting focus groups with a homogeneous composition. There will be 3‐6 focus groups with veterans having TBI and 3‐6 focus groups with their family members/care partners. Each focus group will have 6‐8 participants, resulting in a total sample size of approximately 64 participants. The sample size was determined based on the understanding of what it will take to reach thematic saturation in the field [127-129].

The sample will be recruited with the assistance of the CEC, IEPs, and VA PRCs. We will seek to recruit at least 25% of participants representing a racial/ethnic minority and oversample female veterans.

#### Data Collection

##### Overview

We will use 3 tools in data collection. The first tool is a brief demographic questionnaire to collect the following information from participants: age, race, gender, and date of rehabilitation. The second tool is the Ohio State University TBI Identification Method (OSU TBI-ID) [130] to ensure consistency in identifying TBI exposure. The OSU TBI-ID has established reliability and validity and has been used with veterans to provide lifetime exposure rather than being limited to injuries experienced during deployment [131-136]. The third tool is a focus group interview guide consisting of 8 questions (1 introductory question, 6 main questions about their inpatient rehabilitation experiences, and 1 concluding question). The focus group interview guide will be reviewed and revised during study initiation to ensure the questions are at an 8th-grade reading level and follow federal plain language standards to promote understanding [137]. The focus group guide will be pilot tested with people representing those we will engage to ensure comprehension of the questions and content, and it will be finalized based on the pilot test data for IRB approval prior to use.

##### Focus Groups

To begin our process of adapting the existing STAR-VA manual, 2 trained qualitative researchers will conduct virtual focus groups with veterans having brain injury and with their family members/care partners. The goal of the focus groups is to understand participants’ lived experiences engaging in rehabilitation as it relates to optimizing adaptive behavior. We will seek to understand the acceptability of various behavioral intervention practices from the perspectives of the veterans and their family members/care partners. Participants will be consented and assured of their rights and privacy. They will complete the demographic questionnaire and OSU TBI-ID in advance of the focus groups. The focus groups will last no longer than 90 minutes and will be audio-recorded with their permission. The virtual platform for the focus groups will include closed captioning and verbal descriptions of visual stimuli to improve accessibility.

The focus groups will be conducted by a facilitator who asks questions, probes for additional information, and manages group dynamics (including the use of cognitive accommodations when needed), and a co-facilitator who documents and summarizes participant responses, probes with clarifying questions, and manages recording technology. Focus group recordings will be transcribed and uploaded to ATLAS.ti version 22 (ATLAS.ti Scientific Software Development GmbH), along with any notes taken during the focus groups.

##### Working Groups

IEPs and SMEs, including rehabilitation therapists, nurses, behavioral health providers, physicians, and other team leaders, will be invited to a series of 12 one-hour structured working meetings, which will be scheduled over the course of 18 months. The research team will prepare and distribute materials for review 2 weeks prior to each meeting. Meetings will be recorded with permission and facilitated using a structured agenda outlining meeting goals, key questions, and expected deliverables. At the conclusion of each meeting, attendees will be asked to provide simple plus/delta feedback to evaluate and modify meeting processes in a cycle of continued improvement (unpublished data).

Six meetings will evaluate sections of the manual to determine gaps, identify areas for improvement, and determine modifications to be made based on panel members’ expertise and data insights from the focus groups (aim 3.1a). Another 6 meetings will focus on measurement to identify appropriate outcome measures (eg, reduction in challenging behaviors, increased discharge to home, and improved staff morale/reduced burnout). In preparation for aim 3.2, input from the IEPs and the entire CEC will be sought to (1) adapt an existing measure of capability, motivation, and opportunity to determine the readiness of rehabilitation professionals to implement the TeamBI program [138] and (2) choose items from the CFIR Interview Guide to solicit open-ended responses regarding barriers and facilitators to implementation of the TeamBI program.

##### Playbook Production

The content of the e-playbook will be compiled into a web-accessible electronic package. The package will include chapters, tools (eg, checklists, A-B-C forms, and outcome measures), the implementation readiness measure, and a recorded webinar describing the playbook. Our e-playbook will be compiled to ensure relevance, accessibility, acceptability, and utility to all end users, including those with disabilities. Our digital presence will align with Section 508 of the Rehabilitation Act of 1973 [139].

### Analysis Plan

Focus group transcripts will be analyzed using matrix analysis, a rapid assessment approach, to summarize and identify themes. Rapid assessment is a team-based approach to iteratively collect and analyze qualitative data that emphasizes the speed of data collection and analysis in relation to focused programmatic questions or problems [62]. Two experienced qualitative researchers will conduct the matrix analysis. Together, they will create codes both deductively from known constructs and inductively from the data, and use matrices to categorize responses under domain names, applying codes that emerge from the data. Codes and preliminary themes will be reviewed by the research team and IEPs to establish consensus. Data insights will be aligned with relevant sections of the playbook (eg, experiences of communication, environment, staff behaviors, and family inclusion in inpatient rehabilitation environments) in a brief, consumable report for IEPs to incorporate into the design of the playbook (aim 3.1b).

### Aim 3.2 (Study Months 25-48)

#### Participants

Participants will include at least 30 rehabilitation professionals in team leadership roles that align with program development and implementation. While we anticipate that most will be employed at a VA PRC, some will also be drawn from civilian facilities that serve veterans. Each included participant must (1) be a rehabilitation professional in one of the key leadership roles for TeamBI program implementation (team leader, administrator, behavioral health provider, and nurse manager) or hold a position that includes program development and implementation in job duties, (2) be employed in a TBI inpatient rehabilitation setting that serves V/SMs, and (3) have online access.

Participants will be recruited with assistance from the CEC. To ensure diversity, we will call upon volunteers from the CEC to assist with outreach. Consent will be obtained online prior to commencement of study procedures.

A sample size of n=30 has at least 80% power at 5% significance to detect a change in means associated with an effect size as small as 0.53 (change in mean/pretest SD) [140,141], which is considered to be a medium effect size.

#### Data Collection

The Capability, Opportunity, Motivation to Implement a Team-Based Behavioral Intervention (COM-TBI) model, a pre/posttest instrument based on the COM-B model [142,143], will be used to evaluate the impact of exposure to the playbook on rehabilitation professionals’ likelihood to implement the TeamBI program. The COM-B model purports that in order for change to occur, the individual needs to be motivated; however, motivation will not lead to behavior change unless the individual perceives themselves as capable and having the opportunity to perform the behavior. Typically, studies of behavior change, including the likelihood that an individual will implement a specific program, require the construction of a study-specific measure. However, a generic 6-item measure was developed specifically for adaptation across different studies [138].

The generic questionnaire was tested with health care professionals and a sample of individuals with low socioeconomic status, and was found to be acceptable, understandable, reliable, and valid [138]. Scores on the scale significantly predicted actual behavior change (delivery of opportunistic behavior change interventions by health care professionals). Confirmatory factor analysis indicated a well-fitting 3-factor model aligning with the 3 constructs (capability, opportunity, and motivation). The Bayesian information criterion and Akaike information criterion indicated that the 3-factor model was superior to a unidimensional model. We will adapt this generic measure, tailoring items to be relevant to the implementation of a team-based behavioral intervention program. Since the participants will not have been exposed to the playbook at the time of the baseline pretest, they will be asked to respond based on the implementation of a generic team-based behavioral intervention program for patients with TBI. The CEC and IEPs will assist with item development and piloting prior to use with the study sample. The psychometrics of the adapted instrument will also be evaluated with the study sample.

The CFIR Interview Guide will be used to identify open-ended questions for use in evaluating implementation readiness and the identification of any determinants (barriers and facilitators) that may not have been sufficiently addressed in the playbook. The CFIR guide has been used internationally to study all types of health conditions in military and civilian health care settings [59,60]. The overarching access framework domains from the supply/organization side and consumer/demand side will also inform the focus group questions, and they have been successfully used in our own TBI research [6,46,47,58]. The interview guide will be pilot tested with people representing those we will engage to ensure comprehension of the questions and content. We will include questions about the TeamBI program’s adaptability, complexity, and cost; the design quality and packaging; and the implementation climate at their settings. We will also use the guide’s questions about the individual’s knowledge and beliefs about the TeamBI program, self-efficacy, and the individual stage of change to implement the program. Responses will be used to further refine the playbook.

Information will be obtained from participants regarding age, gender, race, profession/discipline, job title, type of facility where they are employed, whether the facility routinely serves veterans with TBI, whether the facility is a VA or civilian facility, and whether they have online access.

Participants will be sent a link to the consent form, and after they have successfully completed the consent, a link will open a screening form to ensure they meet the inclusion criteria. Participants who meet the inclusion criteria will be linked to the baseline version of the COM-TBI to evaluate capability, motivation, and opportunity to implement a generic team-based behavioral intervention. Upon completing the COM-B, they will be sent the playbook; a link to the online webinar that reviews the playbook’s contents; and a link to the postexposure version of the COM-TBI to evaluate capability, motivation, and opportunity to implement the TeamBI program. They will also be sent the open-ended questions from the CFIR Interview Guide. Upon completion of the postexposure measures, the participants will receive continuing education credit and a financial incentive (US $50). The total time involved is estimated to be 2 hours per participant.

#### Analysis Plan

The sample will be used to test the hypothesis that participants’ perceptions of their capability, opportunity, and motivation will improve from baseline after exposure to the playbook. Means, SDs, and percentiles will be used to summarize continuous data and frequency counts, and percentages will be used to summarize categorical variables. Sample characteristics (eg, age, gender, race, setting [type and civilian vs VA], job title, and discipline) will also be collected and summarized for participants in this sample. Paired *t* tests will be used to test for a change in capability (hypothesis 1), opportunity (hypothesis 2), and motivation (hypothesis 3) from baseline to postexposure at a significance level of α=.05. We will also explore whether setting (VA vs civilian and facility type) has an impact on improvements in knowledge, confidence, and motivation by examining the means, SDs, and effect sizes of each of the outcome variables using analysis of covariance models.

For aim 3.2b, data from the implementation readiness survey will be compiled to identify barriers that may need to be addressed with additional implementation strategies. Our preliminary study yielded a wealth of information from open-ended questions about determinants to implementing behavior programs, and we anticipate that we will receive similarly detailed responses in this study. Open-ended responses will be analyzed using matrix analysis, a rapid assessment approach, to summarize and identify themes. Rapid assessment is a team-based approach to iteratively collect and analyze qualitative data that emphasize the speed of data collection and analysis in relation to focused programmatic questions or problems [81]. Two experienced qualitative researchers will conduct the matrix analysis. Together, they will create codes both deductively from known constructs and inductively from the data, and use matrices to categorize responses under domain names, applying codes that emerge from the data. Codes and preliminary themes will be reviewed by the research team and CEC to establish consensus.

For aim 3.2c, in consultation with the ISC, investigators will refine the playbook based on the findings from aims 3.2a and 3.2b. Strategies will be added for any barriers that were not sufficiently addressed. Any setting-specific barriers will be called out in the playbook with strategies targeted to the setting. Approval of the final playbook will be obtained from the CEC version of the playbook before moving to wider dissemination via the project toolshed.

### Potential Challenges and Solutions

A potential challenge is the possibility of the team facing recruitment difficulties. Recruitment outreach will be expanded using recommendations from the CEC.

Another potential challenge is the possibility that rehabilitation professionals’ perspectives of capability, opportunity, and motivation are not improved by exposure to the playbook. The CEC and ISC will be consulted for suggested revisions and guidance.

### Community-Based Participatory Approach

Community-based engagement is being conducted across project aims and communities. Engagement throughout the project aims is designed to improve outcomes for V/SMs (better access), improve the health care system (system outcomes), and improve implementation (increase buy-in).

### Individual Project 4: Data-Driven Policy Recommendations for Virtual Health Care for Persons With TBI Morbidity

#### Problem and Solution

##### Problem

Universal policy mandates for virtual health to enhance health care access exist in VA and the DOD; however, no research exists for understanding its successful implementation among persons with TBI morbidity. Implementation of virtual health resources has been a priority initiative for VA and the DOD for the past 2 decades because virtual technologies can reduce barriers to accessing care. For example, over 5.5 million veterans actively use the VA’s electronic health portal (My HealtheVet) [144]. JNH and other virtual health experts in the field have spent more than a decade evaluating users’ experiences and implementing HCD efforts to support uptake and sustained use of virtual health resources. However, the COVID-19 pandemic caused a shift toward sustaining access to care, relying heavily on the use of virtual health resources. What was once an option for accessing care has now become a necessity. During the COVID-19 pandemic, implementation efforts and emergency mandates saw a landmark increase in the use of virtual health resources across all health care services [145]. However, the recent universal mandates to increase access do not consider the unique needs of populations who may require accommodations for cognitive impairments, including alternative communication approaches for accessing health care. As the field of TBI systems of care and the health care systems in general embrace protocols that take a “one-model-fits-all” approach to virtual health care resource use, clinicians are identifying barriers that are uniquely present among V/SMs with TBI. To date, there has been little, if any, research focusing on the virtual health care resource needs of persons with TBI.

##### Solution

This project proposes to leverage existing data to develop a taxonomy of domains and themes relevant to access to virtual health care for persons with TBI morbidity. The study will leverage an existing qualitative dataset examining facilitators and barriers to chronic pain care in persons with TBI. The use of virtual health resources was commonly noted but not the focus of the primary study. This proposal offers a cost-efficient approach that will launch this untapped area of science into a *discovery* process, which can inform an HCD approach to providing recommendations regarding the appropriate use of virtual health care delivery to persons with TBI. A preliminary review of the relevant dataset indicates a clear need to re-evaluate the data through a lens to assess barriers to the use of virtual health resources among persons with TBI. Although virtual health resource use emerged as a main theme in NIDILRR analyses, facilitators and barriers to using virtual health care were not explored. The focus of the secondary analysis will be on virtual health resource use in the context of delivering chronic pain care, which is the top comorbidity among persons with military TBI [8]. Analysis of this secondary data will inform the identification of (1) necessary virtual health resource accommodations and (2) policy recommendations needed to determine appropriate virtual health resource use for persons with TBI-related morbidity (eg, cognitive, physical, and behavioral impairments).

### Background

The COVID-19 pandemic shifted how medical care is provided to patients through a rapid increase in virtual health resources and/or the adoption of virtual health resources to facilitate safe, timely, and accessible care remotely [146,147]. Before the pandemic, V/SMs with chronic TBI reported that distance is a barrier to accessing needed health care services; however, they also indicated that virtual health care is a barrier [6]. Virtual health care is mandated to foster remote access to care, particularly for V/SMs in distant/rural locations [145]. However, universal mandates to increase access do not consider the unique needs of populations who may require accommodations for cognitive impairments, including alternative communication approaches, additional resources, and extra time for appointments, in order to optimally access health care. Qualitative data from provider interviews conducted as part of our NIDILRR-funded study on TBI and chronic pain found that most providers (n=63; rehabilitation therapists, medical doctors and nurses, and psychologists from VA, the DOD, and community health care systems) described the use of virtual resources and telehealth as a main facilitator (93%) to providing care to patients during the COVID-19 pandemic [148]. However, TBI morbidity (ie, cognitive impairments) was reported by 63% of the sample (in thematic analysis) as the primary barrier to delivering health care.

A neuropsychologist made the following statement:

*[During COVID-19] I did a fair amount of telehealth which included video, actually no, I’ll take that back. A lot of telephone contacts. Videoconferencing was not as successful due to the cognitive impairment of the patients.*

Qualitative data from TBI providers highlight the need to better understand how virtual health care interventions require adaptation for TBI survivors with cognitive and physical limitations [46,47]. The use of virtual health care emerged as a main theme in facilitating access to care, but barriers persist for V/SMs with TBI and chronic pain. Understanding and developing a taxonomy of access to virtual health care for persons with TBI and chronic pain can inform the accommodations needed to improve access across relevant access framework dimensions. This taxonomy will inform the development of content needed to inform the creation of policy recommendations for the appropriate use of virtual health care resources among persons with TBI. JNH and Bridget A Cotner will leverage previous experience using mixed-methods and CBPR stakeholder engagement strategies with partners to co-create policy recommendations and the dissemination products needed to support the spread and adoption of stakeholder-driven policy recommendations. This project proposes a QI approach that will only engage CEC PPPs for the purpose of developing recommendations and products relevant to the appropriate use of virtual health care resources among persons with TBI morbidity.

### Objectives

**Figure 5.**
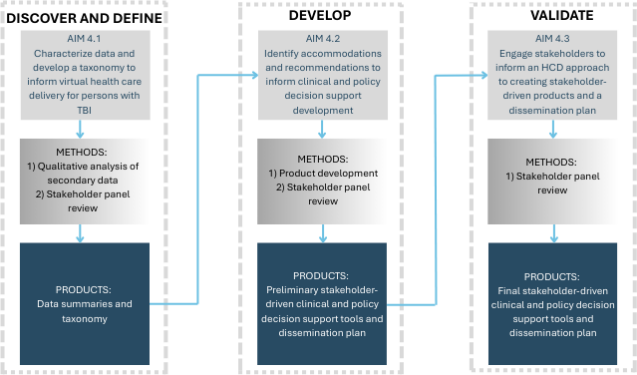
Project 4 aims. HCD: human-centered design.

This project proposes an HCD and CBPR approach with sequential mixed methods to inform the appropriate use of virtual health care resources among persons with TBI (2.5-year implementation study). This proposed project will leverage existing data archived from a multicenter NIDILRR-funded study of TBI health care needs. The aims of project 4 are presented in Figure 5. The aims are as follows:

- Aim 4.1 (discover and define - secondary analyses; 6 months): We aim to characterize data (facilitators and barriers) and develop a taxonomy to inform virtual health care delivery for persons with TBI morbidity.
  - Aim 4.1a: We aim to access and create datasets. Existing provider interview transcripts will be accessed for secondary analyses. This qualitative dataset has been generated from an NIDILRR-funded multicenter trial and includes 63 semistructured interviews with rehabilitation providers across disciplines and settings. Interview questions were informed by the access to health care conceptual framework and focused on treatment and referral practices for persons with TBI and chronic pain, and facilitators and barriers to accessing treatment.
  - Aim 4.1b: We aim to develop and analyze the codebook. Secondary analyses of this existing dataset will allow the identification and organization of data to inform: (1) necessary accommodations and (2) policy recommendations needed to determine the appropriate use of virtual health resources to improve access and user experiences. Upon creation of the preliminary codebook and analysis of 10% of the transcripts, the CEC (PPPs and LEPs) will be engaged for the first stakeholder panel meeting (n=10‐15) to participate in codebook construct definition review and interpretation of initial data findings to inform a stakeholder-driven analytic process in order to ensure relevance, accuracy, and usefulness of the data findings. CEC review and feedback will inform the analysis of the remaining 90% of the data transcripts.
  - Aim 4.1c: We aim to create data summaries and a taxonomy. Coded archived transcripts will be analyzed to create a taxonomy of data findings, which will inform a multilayered approach to understanding the use of virtual health resources among persons with TBI, including facilitators, barriers, opportunities, and recommendations. A second stakeholder panel meeting will engage them for a review of data summaries and for providing input on product development. Finalized data summaries and the taxonomy with stakeholder input will allow data-driven development of products (eg, executive summary, briefing, presentation slide deck, white paper, testimonial, etc) to inform accommodations and recommendations for virtual health care delivery for persons with TBI.
- Aim 4.2 (develop - stakeholder-driven product and dissemination plan development; 1 year): We aim to engage partners to identify the necessary accommodations and recommendations to inform the development of clinical and policy decision-making support for virtual health resource use among persons with TBI morbidity.
  - Aim 4.2a: We aim to conduct gap analysis and draft content. A gap analysis will be conducted to inform the development of a product grid and dissemination plan to inform clinical and policy decision-making products in order to inform accommodations and recommendations for virtual health resource use among persons with TBI. The initial product development product grid will take a preliminary approach to prioritizing: (1) products and deliverable items, (2) target audiences, (3) purpose, (4) content needed, (5) format, (6) complexity, (7) readiness, (8) weighted score (complexity × readiness), (9) pain points, (10) designated roles of developers, and (11) potential impact.
  - Aim 4.2b: We aim to engage the panel to inform product grid, product, and dissemination plan development. An established protocol for product development and validation will be used to re-engage CEC members (PPPs only, ie, key policymakers in the DOD, VA, and civilian health care systems) to participate in a third stakeholder panel meeting (n=8‐10) to review and inform the development of a stakeholder-driven prioritized product grid, product, and dissemination plan to facilitate high-impact data-driven clinical and policy decision support tools. Panel activities will inform the review and weighted scores (complexity × readiness) of products to inform prioritized product development.
  - Aim 4.2c: We aim to develop products and a dissemination plan. Upon finalization of the stakeholder-driven product grid, the products and dissemination plan will be developed (eg, executive summary, briefing, presentation slide deck, white paper, testimonial, etc) to inform clinical and policy decision support relevant to the appropriate use of virtual health resources among persons with TBI.
- Aim 4.3 (validate - products and dissemination plan; 1 year): We aim to re-engage CEC PPPs (ie, key policymakers in the DOD, VA, and civilian health care systems) to inform the HCD approach for creating stakeholder-driven products and a dissemination plan to support data-driven virtual health resource use among persons with TBI morbidity.
  - Aim 4.3a: We aim to compile and deliver content to panelists to prepare for a panel meeting. The team will compile a package of stakeholder-driven products and the dissemination plan and deliver the package to PPP panel members from aim 4.2, with instructions for preliminary review to prepare for the final panel meeting to be conducted in 4.3b.
  - Aim 4.3b: We aim to engage the panel to obtain feedback on the final product grid, products, and dissemination plan. A fourth stakeholder panel meeting (n=8‐10) will be conducted to inform the requirements needed to finalize the product grid, products, and dissemination plan. Panel meeting activities will review the evidence-based process of product development, the final review of products, and the dissemination plan. The PPP panel activities in aim 4 will be the “soft launch” of the dissemination plan, as this aim will rely on key DOD, VA, and civilian health care system representatives who are positioned to champion planned efforts subsequent to this project timeline, to disseminate products for supporting clinical and policy decisions.
  - Aim 4.3c: We aim to finalize the product grid, products, and dissemination plan. Feedback from the fourth, and final, stakeholder panel meeting will inform the finalization of products and the dissemination plan.

### Design

#### Overview

This project will use HCD and CBPR approaches, adopting sequential mixed methods to complete the QI project. Leveraging existing qualitative interview data from the NIDILRR study provides a unique opportunity to conduct secondary analyses to understand specific barriers to treatment and delivery for persons with TBI and chronic pain [46,47]. Investigators and analysts from the parent study are the investigators for the proposed project, who have familiarity with the dataset leveraged for secondary analyses and methods, thus maximizing the success of the study objectives.

In alignment with the I-HEAL theoretical framework, HCD and CBPR approaches, using sequential mixed methods, will be used to complete the QI project. Descriptive qualitative methods will be used to conduct the secondary qualitative data analysis of semistructured provider interview data that have been recently collected (October 2020-November 2021) as part of the NIDILRR-funded “Characterization and Treatment of Chronic Pain after Moderate to Severe TBI” study. CBPR will be used to facilitate stakeholder engagement activities for partnering with key CEC partners throughout the proposed project activities to complete the project aims.

#### Aim 4.1 (Discover and Define - Secondary Analyses; 6 Months)

Two data sources will be utilized to conduct the secondary analysis. First, transcripts of semistructured interviews will query providers about their treatment and referral practices for patients with TBI and co-morbid chronic pain, including what improves or impedes access to treatment. These semistructured interviews consist of 14 questions that are informed by the Access to Healthcare Framework [10]. The qualitative data collection team will utilize probing to ensure important concepts related to access are fully discussed. Second, a provider questionnaire consisting of 14 questions will characterize the provider sample, including information on work settings and the types of patients they treat. No new data will be collected for aim 4.1.

Aim 4.1 will be conducted in 3 phases. In aim 4.1a, data will be accessed and managed for analysis. Dr Bridget A Cotner, an anthropologist and qualitative research expert at Tampa VA who led the primary data collection activities and analysis, will lead aim 1 of this project. The qualitative team, consisting of 2 trained qualitative researchers and led by Dr Bridget A Cotner, has access to the interview data and qualitative analysis software, ATLAS.ti (v.22) [149], to be used for the proposed analyses. Data are housed on a secure password-protected network that is accessible only by qualified research team members. In aim 4.1b, codebook development will be completed. To adequately capture virtual health care access barriers among those with TBI, a qualitative codebook will be developed using deductive codes generated from relevant constructs reflected in the Access to Healthcare Framework and CFIR framework (see Figure 2), and it will be developed inductively from interview data and input from CEC partners, including PPPs, LEPs, and IEPs. As such, once the team develops the initial codebook constructs with definitions and 10% of the transcripts are analyzed, Drs JNH and Bridget A Cotner will facilitate an initial 90-minute virtual or in-person stakeholder panel meeting (n=10‐15) for codebook construct definition review and interpretation of the initial data findings. The stakeholder panel will drive the analytic process to ensure relevance, accuracy, and usefulness of the data findings.

The maximum number of potential participants (PPP, LEP, and IEP group members) is 15, which is beyond the size of a typical group for facilitating a focused group discussion with activities. If the project team determines that the codebook constructs, definitions, and initial analyses are broad and/or complex, the team may opt to divide the PPPs and IEPs into one panel group and the LEPs into another stakeholder panel group. Collectively, the CEC panel meeting review and feedback will inform the analysis of the remaining 90% of the data transcripts.

In aim 4.1c, coded transcripts will be used to create a taxonomy of data findings. Interview transcripts will be recoded by the qualitative team using the codebook augmented by our CEC partner feedback to determine barriers and facilitators to virtual care delivery. Additional codes will emerge inductively from the interview data. The coding team will read one interview transcript separately and discuss the addition of new codes with examples. This process will continue with subsequent transcripts until no new codes are generated. Interrater reliability will be established when the coding team reaches at least 80% coding agreement. Intercoder reliability will be routinely monitored to ensure consistency and limit potential drift in coding. Any discrepancies will be discussed and resolved among PIs, co-investigators, and study staff during weekly meetings to ensure coding and analysis are completed on schedule. Analysts will adopt a constant comparative approach to conduct a matrix analysis [150], a rapid assessment approach [62], which will be used to develop themes for the overall sample by system of care (VA/DOD: n=26; civilian: n=37) and by provider discipline (rehabilitation therapists: n=28; mental health provider: n=15; medical provider: n=15). This analysis will be organized into taxonomies of domains and subdomains that reach saturation. Development of a taxonomy relevant to the informed use of virtual health resources among persons with TBI will provide a formal system for classifying a set of common conceptual domains and dimensions to this multifaceted complex topic. This taxonomy will inform a multilayered approach to understanding: (1) mandated versus actual use of virtual health resources, (2) facilitators and barriers, (3) opportunities and recommendations for virtual health resources for persons with TBI, (4) identified dissemination and implementation strategies, and (5) other factors that surface as relevant to the topic throughout data analysis. A second 90-minute virtual or in-person stakeholder panel meeting will engage them in review of the final data summaries and taxonomy to provide input on product development and dissemination planning. Stakeholder input will inform stakeholder-driven product development (eg, executive summary, briefing, presentation slide deck, white paper, testimonial, etc).

Qualitative data analysis relies on the saturation of data to validate findings, that is, when no new information emerges. The larger parent study, Characterization and Treatment of Chronic Pain after Moderate to Severe Traumatic Brain Injury: A Qualitative Study (PR00039496), provided a sample size justification based on the principle of “saturation.” Due to the specificity of the proposed analysis and interview participants who are knowledgeable of this topic [128], a smaller sample size is needed. As such, for this secondary analysis, themes are expected to reach saturation for the overall sample by system of care (VA/DOD: n=26; civilian: n=37) and by provider discipline (rehabilitation therapist: n=28; mental health provider: n=15; medical provider: n=15) due to a sample size that is more than adequate.

#### Aim 4.2 (Develop - Stakeholder-Driven Product and Dissemination Plan Development; 1 Year)

Aim 4.2 will be conducted in a multiphase stakeholder engagement process to drive product and dissemination plan development. This aim will involve 3 phases. In aim 4.2a, a gap analysis will be conducted based on aim 4.1 findings and previous experience to inform the development of a product grid to inform accommodations and recommendations for virtual health resource use among persons with TBI. Based on previous experience, the team anticipates that a collective group of products (eg, executive summary, briefing, presentation slide deck, white paper, testimonial, etc) will be required for development to inform clinical and policy decision support relevant to the appropriate use of virtual health resources among persons with TBI. The product development product grid will take a preliminary approach to prioritizing (1) products and deliverable items, (2) target audiences, (3) purpose, (4) content needed, (5) format, (6) complexity, (7) readiness, (8) weighted score (complexity × readiness), (9) pain points, (10) designated roles of developers, and (11) potential impact.

In aim 4.2b, CEC PPP and IEP partners from aim 4.1 will be re-engaged for a third 90-minute in-person or virtual panel meeting (n=8‐10) to present data summaries, the taxonomy, and the preliminary list of products and dissemination activities. Re-engagement efforts will focus on PPPs, those with leadership experience in the areas of virtual care delivery and TBI, and other panel members, as deemed appropriate by the aim 4.1 data findings. The aim 4.2b panel meeting will be recorded, and the facilitators (led by JNH and Bridget A Cotner) will use methods with a semistructured interview script to solicit respondents’ perceptions about the information and marketing needs and preferences to inform and solicit respondents’ recommendations. During the panel meeting, data summaries and the taxonomy will be reviewed and evaluated for validity among panel members, and the proposed products and dissemination activities will be reviewed. The team will work with partners to ensure that the dissemination plan includes (1) products for engaging partners with targeted messages, (2) an overview of scheduled communication activities, and (3) anticipated outcomes for stakeholder groups. During this panel meeting, the team will address the identification of all partners and specific contact information to ensure all potential influencing partners are identified. Troubleshooting barriers and discussing workarounds for current policies on the use of virtual health care delivery for persons with TBI will be specifically addressed. JNH and other team members will prepare and anticipate potential barriers and troubleshooting, as these topics tend to arise during policy development and implementation. During the panel meeting, the team will conduct an “activity” with panel members to prioritize products by level of complexity, readiness, and potential impact. Data received during the panel and field notes will be documented and integrated using an established qualitative rapid content analysis process. Audio recordings will also be transcribed and analyzed using rapid content analysis. Initial analyses from the panel meeting will be converged with the analyzed transcribed data, and this approach will support the validity of the findings. This approach will also maximize the relevance and feasibility of recommendation adoption and policy change throughout the DOD, VA, and civilian health care systems.

Brief follow-up calls may be conducted as needed with panel partners (or small groups of 3‐4 members) to finalize or clarify any unresolved topics that might arise during the virtual panel meeting or during subsequent analysis. This iterative development process allows for the emergence of data and/or recommendations, which, although unforeseen, reflect stakeholder values throughout the study process. Once all data are reviewed and compiled, data and product priority rankings will be used to finalize the project product grid and inform and finalize product and dissemination plan development. In aim 4.2c, product development and dissemination planning will be completed.

#### Aim 4.3 (Validate Products and the Dissemination Plan; 1 Year)

In aim 4.3, the team will re-engage CEC partners to validate the clinical and policy decision-making support products and the dissemination plan. This process will ensure that the products and plan are relevant and useful, and have optimal potential for high impact. Given the professional roles of PPPs, as key DOD, VA, and civilian health care system representatives, this group will be the “front line” of leaders who are uniquely positioned to support dissemination and policy impact. As such, this aim will be the “soft launch” of the dissemination plan, as this aim will rely on those who are positioned to champion planned dissemination efforts.

Aim 4.3 will be conducted in 3 phases. In aim 4.3a, before the third and final panel meeting is conducted, the product grid, content, and dissemination plan will be sent to panel members as a compiled “package,” with instructions for preliminary review in preparation for the third and final panel meeting. Subsequently, in aim 4.3b, the fourth 90-minute panel meeting (in-person or virtual) with CEC PPP and IEP members (n=8‐10) will be scheduled to formally present the final product grid (with ratings, product content, and the dissemination plan) to the panel to prompt discussion, collective review, and synthesis of feedback and recommended revisions. As in previous aims, the facilitators will use a semistructured script to guide major discussion points and activities to ensure that all topics are addressed and planned activities are completed. The team will finalize the product grid, products, and dissemination plan with panelists. Other issues, such as stakeholder contacts, will be confirmed as current, and opportunities for troubleshooting and workarounds will be reconfirmed. As in aim 4.2, this iterative process will allow for the emergence of data and/or recommendations, which may be unforeseen but reflect stakeholder values throughout the project phases. As with aim 4.2b, data received during the panel, data from field notes, and information from transcribed data will be converged and analyzed using rapid content analysis to optimize validity and maximize the relevance and feasibility of recommendation adoption and policy change throughout the DOD, VA, and civilian health care systems. In aim 4.3c, once all data are reviewed and compiled, Drs JNH and Bridget A Cotner will work with ISC team members, particularly Mrs Margeaux Chavez, to take a rapid, systematic approach for finalizing the product grid, products, and dissemination plan to prepare for the launch of the dissemination plan with our panel of CEC partners.

### Potential Challenges and Solutions

The first potential challenge involves the rapid timeline. We will leverage existing data that was previously analyzed by Bridget A Cotner and leverage established protocols used by JNH and Bridget A Cotner in previous projects.

The second potential challenge is the limitations of secondary data findings. Though secondary data may limit insights, we have established grounds for the secondary analysis based on the primary analysis of data. Moreover, these data can inform follow-up data collection as appropriate for future project efforts.

The third potential challenge is a lack of participation by panel members. Our team has expertise in developing and disseminating resources, with stakeholder input, to support the appropriate use of virtual resource implementation with targeted audiences. Invested partners have expressed a willingness to participate on a panel to provide feedback on the development of products and dissemination planning. We will collaborate with our engagement stakeholder group to invite them to participate on a panel to provide rapid feedback on products and communication strategies to disseminate policy recommendations. Panel member participation will be minimized to 3‐5 hours overall.

The fourth potential challenge is the limited impact of dissemination efforts. Previous work conducted by JNH has effectively resulted in policy change and redesign efforts of VA’s electronic health record and virtual health resources (ie, secure messaging), and as such, we have the experience and confidence that appropriate data validation and dissemination should have similar results in the proposed effort. Dr JNH will champion the finalized products and the dissemination with PPP members and the ISC to implement and optimize impact.

### Community-Based Participatory Approach

In alignment with the I-HEAL framework, this project will leverage a CBPR approach to engage the CEC at multiple timepoints in order to inform the process, activities, and development of products and the dissemination plan. Activities to support ongoing involvement will include a virtual/in-person meeting at the beginning of the project to discuss the project’s aims and activities, and discuss the access framework and qualitative findings from initial themes mapped onto the Access to Healthcare Framework. Partners will meet at designated time points to inform analysis, discuss, create, review decision-making support products and the dissemination plan, and support and inform the dissemination plan.

To ensure panel engagement throughout the lifetime of the proposed project, panelists will receive project updates every 6 months to ensure they are aware of project aims, processes, and outcomes, and are motivated to continue to stay engaged throughout the project. The first contact with panelists will be their invitation to participate in the project. During this initial contact, team members will inquire about 6-month updates and solicit panelists’ preferences for the update format (eg, newsletter, “all call,” etc). Panelists’ preferred methods will be used to develop the method and protocol for providing project updates. The final project update will be used to share project findings and final products for dissemination. Drs JNH and Bridget A Cotner will work with PPP stakeholder panel members and the ISC to implement the dissemination plan subsequent to the proposed project activities.

## Results

I-HEAL was funded as an implementation science FPA by Congressionally Directed Medical Research Programs, and start-up activities began in October 2023. After IRB approval (projects 1‐3: August 12, 2024; project 4: December 2, 2019), all 4 projects are currently underway, with funding through September 2027. Project 1 has enrolled 48 participants, and project 3 has enrolled 34 participants through September 2025. Project 2 has completed the environmental scan and has identified both general and specific EBTs for inclusion in the toolkit. Project 4 has completed the secondary data analysis and is working on dissemination.

## Discussion

### Rationale and Expected Impact

The I-HEAL protocol contributes to the field in 4 major ways. First, it supports the overall responsiveness to the health of V/SMs, as previous research has highlighted disparities in populations with TBI and specifically military populations with TBI. Second, it supports scientific advances in the delivery of evidence-based care requiring integration and the use of implementation science in the field of TBI. Third, it creates knowledge products from both synthesized scientific evidence and our project innovations in the health care field that are critical to timely knowledge translation. Fourth, it supports the engagement of V/SMs, their families, and health care system SMEs from VA and the DOD.

### Challenges

Potential challenges of the overall I-HEAL effort include (1) interdisciplinary collaboration in a new area of translation, requiring training and novel communication strategies across respective fields of science; (2) a dearth of capacity in the field of TBI and implementation science, thus requiring additional efforts to effectively communicate broadly and transparently to the field; (3) budgetary constraints to respond to new/novel strategies of engagement from established partners during the course of the grant; and (4) funding opportunities to address translational next steps, including sustainment planning for each of the individual projects and core activities.

### Dissemination

The cores are actively working to support projects through partner engagement, implementation science consultation, administration, research methodology, and creating dissemination venues such as I-HEAL’s website and social media accounts (Multimedia Appendix 3). Additionally, the projects have completed 4 podcasts and 39 presentations at well-known conferences, such as the Military Health System Research Symposium and the American Congress of Rehabilitation Medicine, and will continue to present and publish findings.

### Conclusion

The individual preimplementation phase projects in the proposal are a series of innovations to improve access to health care by overcoming barriers that have led to unmet care needs, delivery of non–evidence-based care, and subsequent health care disparity for V/SMs with TBI-related morbidity (cognition, behavior, and psychological functioning). Through HCD, engagement, and iterative evaluation, clear success measures will inform project continuation (Multimedia Appendix 2). Subsequent protocols should focus on the next stages of the implementation and sustainability of the innovations.

## Supplementary material

10.2196/79738 Multimedia Appendix 1 Impact and relevance to military health.

10.2196/79738 Multimedia Appendix 2 Statement of work.

10.2196/79738 Multimedia Appendix 3 Transition plan.

10.2196/79738 Multimedia Appendix 4 Community-based participatory research approach.

10.2196/79738 Multimedia Appendix 5 Community Engagement Council schedule.

10.2196/79738 Multimedia Appendix 6 Communication strategy.

10.2196/79738 Multimedia Appendix 7 Leadership.

10.2196/79738 Multimedia Appendix 8 Cognitive nudge toolkit development.

10.2196/79738 Multimedia Appendix 9 Individual engagement partner areas of subject matter expertise.

10.2196/79738 Peer Review Report 1 Peer review report from the U.S. Army Medical Research and Development Command Congressionally Directed Medical Research Programs, Fiscal Year 2022 Traumatic Brain Injury and Psychological Health Research Program, Focused Program - Healthcare Access Review Panel (Department of Defense, USA).

## Abbreviations

CBPR: community-based participatory research
CEC: Community Engagement Council
CFIR: Consolidated Framework for Implementation Research
COM-TBI: Capability, Opportunity, Motivation to Implement a Team-Based Behavioral Intervention
DOD: Department of Defense
EBT: evidence-based behavioral treatment
EMR: electronic medical record
FPA: Focused Program Award
HCD: human-centered design
I-HEAL: Improving Health Care Access and Engagement for Veterans and Service Members with TBI Morbidity
IEP: individual engagement partner
IRB: Institutional Review Board
ISC: Implementation Science Core
LEP: lived experience partner
MPI: multiple principal investigator
MSKTC: Model Systems Knowledge Translation Center
NASEM: National Academies of Science, Engineering, and Medicine
NDSC: National Data and Statistical Center
NIDILRR: National Institute on Disability, Independent Living, and Rehabilitation Research
NIH: National Institutes of Health
OSU TBI-ID: Ohio State University TBI Identification Method
PCORI: Patient-Centered Outcomes Research Institute
PEP: project engagement partner
PI: principal investigator
PM: project manager
PPP: policy and professional partner
PRC: polytrauma rehabilitation center
PTSD: posttraumatic stress disorder
QI: quality improvement
QUERI: Quality Enhancement Research Initiative
RE-AIM: Reach, Effectiveness, Adoption, Implementation, Maintenance
SME: subject matter expert
SOP: standard operating procedure
STAR-VA: Staff Training in Assisted Living Residences-Veterans Affairs
TBI: traumatic brain injury
TBIMS: TBI Model Systems
TeamBI: Team-based Behavioral Interventions
V/SM: Veteran and Service Member
VA: Veterans Affairs
WDMC: Website and Data Management Core
