# Supplementary material for: Using Implementation Science to Improve Health Care Access and Quality for People With Traumatic Brain Injury–Related Morbidity (I-HEAL): Protocol for a Translational Multiproject Program Award
Source: JMIR Res Protoc. 2026 Mar 6;15:e79738. doi: 10.2196/79738 (PMC12995600; doi:10.2196/79738)
Supplement: Multimedia Appendix 1 [file resprot-v15-e79738-s001.pdf]

Impact and Relevance to Military Health

This proposal aligns with the TREAT Focus Area by using innovation development to address improvements in systems of care by enhancing access and delivery of health care services for persons with TBI-related cognitive, physical, or behavioral challenges (morbidity). The proposal focuses upon a gap highlighted in the National Academies of Science, Engineering, and Medicine (NASEM) report on accelerating progress in TBI, specifically, promoting research translation using implementation science. The individual studies in the proposal are a series of innovations to improve access to healthcare by overcoming barriers which have led to unmet care needs, delivery of non-evidence-based care, and subsequent healthcare disparity for Veterans and Service Members (V/SM) with TBI related morbidity (cognition, behavior, psychological functioning). The individual studies uniquely target supply and demand dimensions that influence healthcare access to optimize outcomes by utilizing implementation science interventions paired with a community participatory action-based approach (Focus Area 3C and Secondary Focus Area 3A). As requested in the funding opportunity announcement, this proposal will address implementation of evidence-based treatments and engage stakeholders to promote adoption of evidence-based care to promote optimal outcomes after military-related TBI.

Overall Responsiveness to V/SM Health. Our work in the NIDILRR and VA TBI Model Systems has highlighted persons with TBI and greater neurologic morbidity are 6.9 times more likely to die than the general population resulting in a 12.2 years reduction in life expectancy. When examining service utilization, we reported that those with greater neurologic burden experience a two-fold increase in rehospitalization rates in the first year post-TBI compared to those with lesser neurologic burden. Veterans and Service Members have higher rates of rehospitalization (44%) compared to civilians with TBI in the Model Systems. Further, those with greater neurologic burden have higher rates of death with distinct causes of early mortality (self-inflicted deaths and preventable injuries) across groups. TBI morbidity (impaired cognition, behavioral regulation) coupled with polytrauma comorbidity (depression, PTSD, chronic pain, sleep disorders) impacts access and meaningful engagement in high quality healthcare to reduce these poor healthcare outcomes. This disparity is further evidenced by our work highlighting unmet healthcare needs in chronic stages and comorbidities associated with worse outcomes. The recent National Academy of Science, Engineering, and Medicine Report on Accelerating Progress in TBI recognizes and calls for action to improve healthcare access for persons with TBI. Poor access to healthcare is an inequity that can be addressed by employing implementation science. The NASEM highlights a dearth of implementation science research in TBI that could help improve access and close the quality-of-care chasm that persons with TBI disability experience.

Scientific advances in the delivery of evidence-based care require integration and use of the field of implementation science in traumatic brain injury. There is a growing recognition of significant delays in research translation across scientific fields. The emerging field of implementation science (IS) has been recognized as a critical field in the research translation process. Implementation science is complex and requires the use of mixed methods approaches to understand, implement, and examine outcomes associated with promoting the use of evidence-

based care in practice. The field also recognizes that the use of evidence-based practice requires adaptations or innovations in how care is delivered (to retain technical quality, fidelity) across healthcare systems (e.g., VA, DOD, civilian, rural, urban settings) and populations (e.g., disabled vs health cohorts). Persons with TBI-related morbidity and comorbidity experience decreased access to healthcare in general including known evidence-based treatments for physical and psychological healthcare. The purpose of this proposal is to address this healthcare inequity informed by the VA QUERI Implementation Roadmap which defines three distinct stages to implementation science research (Pre-Implementation, Implementation, and Sustainment). All projects fall in the Pre-Implementation Phase (adaptation and innovation to prepare for implementation) which often includes identifying stakeholders, understanding needs, and knowledge product development to facilitate the next stage (implementation).

Knowledge products that synthesize scientific evidence from the literature or represent innovations in healthcare are critical to timely translation efforts. The VA QUERI Roadmap highlights phases of translation to bring evidence-based healthcare to patients. In the Pre-Implementation Phase (focus of this proposal), knowledge products are needed that clarify the problem being addressed, best treatment practices, and stakeholders involved. The knowledge products generated across cores and projects (using CBPR) will be informed by several conceptual frameworks used in the field of Implementation Science described in the Core descriptions. The second Implementation Phase includes preparing for and conducting implementation to promote use evidence-based or innovative care. The Sustainment phase evaluates long-term impacts and assignment of implementation ownership. Given the scope of the FOA, actual implementation (Stage 2) and sustainment phases (Stage 3) of research will be addressed in future proposals and are described in the Transition Attachment. Throughout this process, new questions arise which require generation of new data and re-initiation of the roadmap to address evolution of the healthcare delivery. The proposed FPA will generate products relevant for the Pre-Implementation and Implementation Phases of this model. Knowledge products will be produced targeting key stakeholders in the adoption of study findings which include 1) other scientists, 2) clinicians (indicate types), and 3) policymakers, 4) hospital administrators, 5) trainees, and 6) persons with traumatic brain injury and their families. As products are finalized, they will be housed on the study website which will serve as a repository of tools (a Toolshed) available to the public with links pointing from engagement partners.

Engagement of V/SMs, Families, and health care system subject matter experts from VA and DOD are engaged in all aspects of the study. To reflect the lived experience of military TBI, V/SMs and their families (n=9) will serve on the Community Engagement Council as Lived Experience Partners (CEC-LEP). As members of the CEC-LEP, they will meet with project leadership monthly and with individual project teams throughout the conduct of the study as outlined in individual project descriptions. VA and DOD administrators, providers, and executive stakeholders at the national level serve as Community Engagement Council Policy and Professional Partners (CEC-PPP). Major professional organizations key in the implementation of proposed innovations in care will meet quarterly with project leadership and individual study teams throughout. In addition, members of the CEC-PPP include VA employees and Veterans

with professional understanding of military TBI. Individual studies further engage military and lived experience subject matter expertise relevant to their project in the development, execution, and dissemination of deliverables.

Overall Focused Program Award Impact. The individual studies in the proposal are a series of innovations to improve access to healthcare by overcoming barriers which have led to unmet care needs, delivery of non-evidence-based care, and subsequent healthcare disparity for V/SM with TBI related morbidity (cognition, behavior, psychological functioning). As requested in the funding opportunity announcement, this proposal will address implementation of evidence-based treatments and engage stakeholders to promote adoption of evidence-based care to promote optimal outcomes after military-related TBI. In the short term, product development and testing will occur across projects that are critical in the translation process. Engagement of stakeholder communities throughout increases likelihood of adoption across clinical settings. The I-HEAL proposal enhances clinical care by proposing four synergistic projects that will improve access and engagement in high-quality, evidence-based healthcare services for V/SM with TBI morbidity. Collectively the four projects propose to accomplish the following overarching objectives of the FPA: (1) adapt existing interventions to promote access and engagement in healthcare; (2) engage stakeholder communities to maximize uptake and translation; (3) promote research translation that informs policy and practice through knowledge translation products and deliverables targeting key stakeholders (clinicians, Veterans, Service Members, caregivers, policymakers, and other researchers); (4) facilitate research and implementation to enhance access to high-quality healthcare for V/SM with TBI-related morbidity; and (5) foster development of early/mid-career researchers in advancing implementation science research on access to care for V/SM with TBI. All project deliverables have translation potential to civilian healthcare settings to benefit public health.

Project 1 Problem

TBI has been described as an invisible disability because TBI causes substantial cognitive impairments without overt physical evidence of injury. Cognitive impairments can interfere with a TBI survivor's ability to engage in evidence-based treatment during health care encounters. Indeed, up to 50% of V/SM report needing assistance with accessing and coordinating medical services. Likewise, health care access was reported as an unmet need by family members and care partners (i.e, health care proxies) of persons with TBI. Involvement of a health care proxy (often a family member) has been found to improve access to services and health outcomes among cognitive impaired persons with TBI. Unfortunately, health care providers may not be aware of the need to involve health care proxies during health care encounters, with 37% reporting that they excluded from health care to the detriment of their loved one with TBI. (Solution). The project will engage stakeholders in VA, DOD, and civilian healthcare settings to design, develop, and pilot able an electronic medical record (EMR) flag-based cognitive nudge and implementation toolkit in a TBI VA polytrauma rehabilitation center. This innovation will systematically alert health care providers to modify usual practice by proactively including a health care proxy in the delivery of evidence-based treatment during health care encounters with persons with cognitive impairment due to TBI.

Project 1 Impact

Near-term impact: At the conclusion of this project, the study team will have co-designed an EMR flag-based (cognitive nudge) in collaboration with multiple stakeholder groups, including TBI health care providers, health care administrators, and persons with lived TBI experience (TBI survivors and their care partners/health care proxies). We will have user tested the cognitive nudge and gathered pilot data on its implementation at one of the flagship VA Polytrauma Rehabilitation Centers, thus generating empirical data supporting its utility in reducing health care access barriers among cognitively impaired Veterans with TBI who are vulnerable to health care disparities due to limitations in their ability fully engage in evidence-based treatments during and between health care treatment encounters. Future Veterans with TBI seen at this institution will have the opportunity to receive the EMR flag-based cognitive nudge and subsequently have their health care providers receive prompts to include needed health care proxies during health care encounters.

Long-term impact: The development and pilot implementation of the cognitive nudge will have set the foundation to other health care settings (within and outside VA and Military systems), as well as other V/SM populations with cognitive impairment due to etiologies beyond TBI. The cognitive nudge implementation blueprint developed for this study can be integrated at other VA hospitals and clinics and translated to civilian settings by targeting systems-level change, such as adoption of the cognitive nudge into EMRs via organizational and policies, clinical practice guidelines, and standards and recommendations by agencies that accredit health care programs; examples include Veterans Health Administration (VHA) standard operating procedures, Joint Commission on Accreditation of Healthcare Organizations, Commission on Accreditation of Rehabilitation Facilities, and Centers for Medicare & Medicaid Services.

Project 2 Problem

Proposal investigators have robustly shown that cognitive morbidity (i.e., cognitive impairment) is the number one rehabilitation need in chronic stages of TBI with unmet needs associated with poorer satisfaction with life among V/SMs. Further, V/SMs with greater TBI disability (and greater cognitive morbidity) experience unique barriers to accessing care in chronic stage of TBI. Our most recent work characterizing healthcare delivery for the most common comorbid conditions in TBI highlights cognitive morbidity as a barrier in referral to and receipt of evidence-based behavioral treatments (EBTs), limiting healthcare access and meaningful engagement in management of TBI comorbidities that may improve outcomes (i.e., suicide risk, PTSD severity, arrest rates, worsening cognition). (Solution). The proposed project will address determinants (i.e., facilitators and barriers) that limit a cognitively impaired person's ability to engage in EBTs for the most common comorbid conditions (i.e., PTSD, depression, sleep disorders, chronic pain) in TBI by (Aim1) conducting an environmental scan to identify existing EBT adaptations for persons with cognitive impairments, (Aim 2) selecting adaptations identified through a community-based participatory research (CBPR) approach, and (Aim 3) developing and disseminating a Provider Toolkit for Accommodating Cognitively Impaired Persons that will aid identification and engagement of cognitively impaired persons in EBTs.

Project 2 Impact

Near-term impact: At the conclusion of this project, we will have developed a product grid matching adaptations for cognitive-deficits onto evidence-based intervention strategies for the most commonly comorbid psychological and behavioral health conditions among those following TBI. Our product grid will inform a consolidated toolkit for clinicians and providers and will address gaps we have identified among providers in both the referral to (i.e., access to services) and engagement in services among those who have sustained cognition altering TBI. These evidence-based behavioral interventions (e.g., cognitive behavioral therapies, acceptance and commitment therapy, motivational interviewing) are front-line treatments for common V/SM co-occurring health problems such as sleep disorders, chronic pain, and PTSD. We will maximally engage stakeholders from multiple ecological perspectives to validate toolbox strategies in the care of the comorbidities in a behaviorally and cognitively complex clinical population. Our stakeholders will feature providers, patients and family members with military backgrounds and who deliver services within MHS and VHA facilities where context relevant perspectives will make the most significant impact in the lives of V/SM who have sustained TBI. This toolkit will empower providers, patients, care partners and family members to make supportive adaptations more readily within care and home environments.

Long-term impact: Having developed this toolkit, we will be poised to facilitate next steps in implementation of these recommendations to determine feasibility and evaluate their ability at increasing engagement in evidence-based, behavioral (ie. nonpharmacological treatments) in this population. Our toolkit will facilitate engagement in evidence-based interventions among those with TBI long-term by consolidating and enhancing useability of adaptation strategies that can be broadly applied to (1) address cognitive deficits (i.e., transdiagnostic) as well as (2) comorbidity specific treatment adaptations, such that it may inform implementation research in a variety of specified clinical contexts and conditions. Our development of this toolkit with input from diverse stakeholders will facilitate its utility across settings where military and civilian cohorts with post-TBI comorbidities are likely to be treated. This will optimize messaging for successful dissemination and implementation in our next phase of research along the implementation roadmap. The study team is composed of subject matter experts in delivery of adapted care in this population and implementation science, and who have a history of translating research knowledge to inform care in medically complex populations.

Project 3 Problem

Challenging or maladaptive behaviors after moderate-severe TBI (e.g., agitation, impulsivity) are common (44-74%), frequently impacting access and quality of healthcare. Our preliminary data indicate that these challenging behaviors are associated with denial of access to healthcare settings for persons with TBI, as well as increased rehabilitation staff injuries and turnover. Possibly due to perceived lack of alternative options, one-third of providers worldwide have reported treating challenging behaviors with sedating medications thought to impede adaptive behavior, cognition and neuro-recovery. While effective methods for optimizing adaptive behavior while reducing challenging behaviors exist, widespread implementation of evidence-based, non-pharmacological treatment programs has not yet occurred. (Solution). We will develop and pilot a Playbook for implementing an interdisciplinary program in TBI inpatient rehabilitation settings to promote adaptive behavior and reduce challenging behavior of patients

with TBI. The Staff Training in Assisted Living Residences-VA (STAR-VA) will be adapted to produce the new program, TeamBI: Team-based Behavioral Interventions. This program will incorporate the principles of applied behavior analysis and Positive Behavior Support (PBS) to prepare interdisciplinary teams in inpatient rehabilitation settings to set up the environment to optimize adaptive, positive behaviors and minimize challenging behaviors. The Playbook will also provide implementation strategies to assist with establishing the program.

Project 3 Impact

Near-term impact: Expected outcomes include a manualized intervention with an implementation plan to promote adoption into programs. Engagement of partners representing varied stakeholders in managing maladaptive behaviors on inpatient rehabilitation units (nursing, psychology, therapy providers, PMR administration) and professional organizations (e.g., social work, rehabilitation psychology, physiatry) on the Community Engagement council maximize translation and adoption by commenting on the evolution of study products, identifying venues for provider education, and endorsement as meeting accreditation standards such as those by CARF International. Professional organizations may partner to disseminate formal training programs. The infrastructure of the American Congress of Rehabilitation Medicine Adult Training Institute could be utilized to offer training programs and accompanying manualized interventions (owned and distributed by that organization).

Long-term impact: Mechanism and materials for training the rehabilitation workforce in delivering evidence-based care for maladaptive behaviors has the potential to improve patient outcomes in a myriad of ways. A trained workforce is prepared to manage these neurologic symptoms thus may improve admissions to rehabilitation programs. It may also result in lowering reliance on sedating medications (delivering the wrong treatment) and thus improve rehabilitation outcomes because V/SM are able to better engage in rehabilitation therapy. From a health system standpoint, a trained workforce may result in lower turnover in VA employment among front-line nursing staff working in these programs (a major hiring challenge for VA/DOD), staff injuries, and employee satisfaction.

Project 4 Problem

Universal policy mandates for virtual health to enhance healthcare access exist in VA/DOD; however, no research exists understanding its' successful implementation for persons with TBI morbidity. Implementation of virtual health resources has been a priority initiative for VA and DOD for the past two decades because virtual technologies can reduce barriers to accessing care. For example, over 5.5 million Veterans actively use the VA's electronic health portal [My HealtheVet (MHV)]. Project 4 team members and other virtual health experts in the field have spent more than a decade evaluating user's experiences and implementing human-centered design (HCD) efforts to support uptake and sustained use of virtual health resources. However, the COVID-19 pandemic shifted to the priority of sustaining access to care, relying heavily on use of virtual health resources. What had once been an option for accessing care, has become a necessity. During the COVID-19 pandemic, implementation efforts and emergency mandates saw a landmark increase in the use of virtual health resources across all healthcare services. However, the recent universal mandates to increase access do not consider unique needs of

populations who may require accommodations for cognitive impairments, including alternative communication approaches for accessing healthcare. As the field of TBI systems of care – and healthcare systems in general – launch into protocols which take a “one model fits all” approach to virtual healthcare resource use, clinicians are identifying barriers that are uniquely presented with V/SM with TBI. To date, there has been little, if any, research focusing on the virtual healthcare resource needs for persons with TBI. (Solution). This project proposes to leverage existing data to develop a taxonomy of domains and themes relevant to access to virtual healthcare for persons with TBI morbidity. The study will leverage an existing qualitative dataset examining facilitators and barriers to chronic pain care in persons with TBI. The use of virtual health resources were commonly discussed by participants but not the focus of the primary study. This proposal offers a cost-efficient approach that will launch this untapped area of science into a discovery process which can inform a HCD approach to informing recommendations regarding appropriate use of virtual healthcare delivery to persons with TBI. Preliminary review of the relevant dataset indicates a clear need to re-evaluate the data with a lens to assess barriers to use of virtual health resources with persons with TBI. Although virtual health resource use emerged as a main theme in NIDILRR-analyses, facilitators, and barriers to using virtual healthcare were not explored. The focus of the secondary analysis will be on virtual health resource use in the context of delivering chronic pain care which is the top comorbidity in persons with military TBI. Analysis of this secondary data will inform identification of: (1) necessary virtual health resource accommodations; and (2) policy recommendations needed to determine appropriate virtual health resource use for persons with TBI-related morbidity (e.g., cognitive, physical, behavioral impairments).

Project 4 Impact

Near Term Impact: At the conclusion of this project, the team will have developed clinical and policy decision making support tools and products and engaged key policymakers to deploy the tools and products. Findings from our secondary data analysis (aim 4.1) will identify determinants that support or hinder virtual healthcare access and delivery. These findings along with our diverse stakeholders representing operational partners, clinical providers, V/SMs, family members, and subject matter experts will inform the creation of decision-making support documents and a communication plan to inform recommendations for virtual healthcare delivery for persons with TBI morbidity. Aim 4.2 and 4.3 will engage stakeholders in the creation and deployment of these materials to raise awareness and inform decisions at the individual/family, provider/organizational, and policymaker/national levels.

Long-term impact: With the development of recommendations and clinical and policy decision making support products, the initial deployment through dissemination to key organizations and stakeholders will be poised for implementation of these recommendations and products to determine feasibility and evaluate their ability to improve virtual healthcare delivery for persons with TBI morbidity. The study team is composed of subject matter experts in implementation science, virtual healthcare, adapted care in this population, and who have a history of translating research knowledge to inform care in medically complex populations.

Potential Challenges and Solutions

Project 1: How will the team maximize acceptance of the Cognitive Nudge among persons with lived experience. We have considered the potential disadvantages of an EMR flag alerting providers to a patient's cognitive challenges, and we will work with our lived experience partners (stakeholders) to determine best practices for deferring and/or removing the flag for any reason, including patient/family preferences. Better understanding what aspects of a nudge intervention may be less attractive to those with lived experience is essential for designing the toolkit proposed herein. Based extensive data presented above from our team and others, a plurality of TBI survivors living with cognitive impairments report that these impairments pose barriers to care; the current study will determine how best to mitigate those barriers through co-development of a decision support tool in partnership with stakeholders.

Project 2: What if there are no respondents to the survey of the environmental scan? Team members have had success recruiting from professional organizations using informal methods. Formal engagement and buy-in from these organizations from the start of the project help facilitate commitment to engaging their respective constituencies for input.

Project 3: What if rehabilitation professionals' perspective are not improved by exposure to the training materials for managing challenging behaviors? Solution: To maximize acceptance and benefit of the materials developed, the team will engage with members of the Community Engagement Council which includes end-users of the knowledge products being developed.

Project 4: What if there is limited impact of dissemination efforts? Previous work conducted by Haun has effectively resulted in policy change and redesign efforts of VA's electronic health record and virtual health resources (i.e., secure messaging), as such we have the experience and confidence that appropriate data validation and dissemination should have similar results in the proposed effort.
