# Supplementary material for: Using Implementation Science to Improve Health Care Access and Quality for People With Traumatic Brain Injury–Related Morbidity (I-HEAL): Protocol for a Translational Multiproject Program Award
Source: JMIR Res Protoc. 2026 Mar 6;15:e79738. doi: 10.2196/79738 (PMC12995600; doi:10.2196/79738)
Supplement: Multimedia Appendix 2 [file resprot-v15-e79738-s002.pdf]

Overall Program Award Statement of Work (GANT Chart) – See Project Specific Details on Subsequent Pages

Table S1. Overall FPA Implementation Milestones and Timeline

Year	Year 1				Year 2				Year 3				Year 4			
**Quarter**	**1**	**2**	**3**	**4**	**1**	**2**	**3**	**4**	**1**	**2**	**3**	**4**	**1**	**2**	**3**	**4**
**1.0 Overall Project Activation (Responsible Party: MPIs)**																
1.1 Complete Prime Contract																
1.2 Initiate Subawards																
1.3 Hire FPA Staff (non-trainees)																
1.4 Establish calendar invites for communication strategy																
1.5 Regulatory declaration, union approvals etc.																
**2.0 Web and Data Management Core Activation (Responsible Party: Mellick, Ketchum)**																
2.1 Obtain Feedback on Public Facing Website Design																
2.2 Quantitative Data Capture and Analysis																
2.3 Finalize Content and Deploy Toolshed Website with Ongoing Product Updates																
**3.0 Implementation Science Methods Core Activation and Ongoing Activity (Responsible Party: Haun)**																
3.1 Implementation Science Methodology Training																
3.2 Qualitative Data collection, Analysis, and Product Development																
3.3 Implementation Science Training Registry																
**4.0 Activate Professional and Patient Engagement Communities (Responsible Party: Corrigan, Moore, Raadwan)**																
4.1 Initiate Videoconference Meetings																
4.2 Conduct In-Person Engagement Meetings																
4.3 Define and Measure Successful Engagement																
**5.0 Project Initiation and Conduct (Responsible Party: Project MPIs)**																
5.1 Project 1 *(See Detailed GANT Chart in Project Description)*																
5.2 Project 2 *(See Detailed GANT Chart in Project Description)*																
5.3 Project 3 *(See Detailed GANT Chart in Project Description)*																
5.4 Project 4 *(See Detailed GANT Chart in Project Description)*																
**6.0 Dissemination (Responsible Party: Overall MPIs, Core Directors, Project MPIs)**																
6.1 Meetings and Conferences																
6.2 Manuscript Submission																
6.3 Data sharing activities																

Project 1 Statement of Work (GANT CHART)

Project 1: Cognitive Nudge Decision Support Tool Milestones																			
	Year 1				Year 2				Year 3				Year 4				Core Involved		
**Item**	**Q1**	**Q2**	**Q3**	**Q4**	**Q1**	**Q2**	**Q3**	**Q4**	**Q1**	**Q2**	**Q3**	**Q4**	**Q1**	**Q2**	**Q3**	**Q4**	**WDMC**	**ISC**	**CEC**
**Study Start-Up.** *Responsible Party: Project Manager, MPIs*																			
Meetings																		X	
Regulatory																		X	
**AIM 1 (Discover): Identify best practices, standards and preferences.** *Responsible Party: Project Manager, MPIs, Cores, IEPs.*																			
Survey Development																	X	X	
Survey Deployment																	X		X
Survey Analysis																	X	X	
Lived Experience Focus Groups																		X	X
Provider Focus Groups																		X	X
Publish Aim 1 Findings																	X	X	
**AIM 2 (Design): Codesign nudge and adapt existing policies and procedures.** *Responsible Party: Project Manager, MPIs, Cores, IEPs.*																			
Meeting 1-2																		X	X
Meeting 3																			X
Meeting 4-6																			
Meeting 7-9																		X	X
**AIM 3 (Deliver, Validate, Disseminate): Pilot Nudge within VHA System.** *Responsible Party: Project Manager, MPIs, Cores, IEPs.*																			
EMR Integration																			X
Staff Training																			X
Pilot																			
Formative Eval																		X	X
Refinement																		X	
Dissemination																	X	X	X

Project 2 Statement of Work (GANT Chart)

Project 2: Provider Toolkit for Accommodating Cognitively Impaired Persons in Evidence Based Treatments																			
	Year 1				Year 2				Year 3				Year 4				Core Involved		
**Item**	**Q1**	**Q2**	**Q3**	**Q4**	**Q1**	**Q2**	**Q3**	**Q4**	**Q1**	**Q2**	**Q3**	**Q4**	**Q1**	**Q2**	**Q3**	**Q4**	**WDMC**	**ISC**	**CEC**
**Project Start-Up.** *Responsible Party: Project Manager, MPIs, Cores, IEPs*																			
Investigator/Study Team Meetings																			
Regulatory/Quality Improvement Determination																			
**Aim 1 (Discover): Environmental Scan and Product Grid Development.** *Responsible Party: Project Manager, MPIs, Cores, IEPs.*																			
Engage CEC and IEP/SMEs to validate scope of environmental scan and criteria for product grid																			x
Develop and launch Qualtrics survey with social media specialize to collect materials and tools																	x	x	
Meet with PEP and request materials, disseminate Qualtrics link broadly																	x	x	x
Environmental scan initiated by research coordinator and continued by post-doctoral fellows with MPI/Study Team Oversight																		x	
**Milestone**: Completed Product Grid						X												x	
**Aim 2 (Define and Design): SME Review, Development of Toolkit Prototype.** *Responsible Party: Project Manager, MPIs, Cores, IEPs.*																			
Convene SMEs to review and evaluate product grid, prioritize products and identify gaps																		x	x
Create content for any missing/needed products																		x	x
Convene LEP for feedback on acceptability of toolkit main points/overview																		x	x
Develop, format, and organize toolkit content																		x	
**Milestone**: Completed Toolkit Prototype												X							
**Aim 3 (Validate): Refine and Validate Toolkit through Formative Review.** *Responsible Party: Project Manager, MPIs, Cores, IEPs.*																			
Convene PEP for formative review and begin identifying dissemination targets																		x	x
Convene SME review panel for formative review																		x	x
Convene LEP for formative review																		x	x
Refine toolkit from formative review feedback																		x	
Share finalized version of toolkit with engagement partners																			x
Engage PEP to establish dissemination plan for toolkit to reach providers with PEP																		x	
**Milestone**: Completed toolkit ready for early dissemination, future pilot testing																X			

Project 3 Detailed Statement of Work (GANT Chart)

**Project 3: Empowering Teams to Implement Evidence-Based Behavioral Interventions (TEAM-BI)**																			
	**Year 1**				**Year 2**				**Year 3**				**Year 4**				**Core Involved**		
**Item**	**Q1**	**Q2**	**Q3**	**Q4**	**Q1**	**Q2**	**Q3**	**Q4**	**Q1**	**Q2**	**Q3**	**Q4**	**Q1**	**Q2**	**Q3**	**Q4**	**WDMC**	**ISC**	**CEC**
**Study Start-Up.** *Responsible Party: Project Manager, MPIs*																			
Meetings																			
Regulatory																			
**AIM 1 (Design and Develop): Identify best practices, standards, preferences; Develop Playbook.** *Responsible Party: Project Manager, MPIs, Cores, IEPs*																			
Recruit, conduct and analyze focus groups																			X
Working groups																		X	
Playbook production																	X		
**AIM 2 (Refine and Deliver): Validate, refine and disseminate playbook.** *Responsible Party: Project Manager, MPIs, Cores, IEPs*																			
Recruit sample and run pilot																			
Analyze quantitative and qualitative data																	X		
Refine playbook, obtain CEC approval for dissemination																	X	X	X
Load Playbook into toolshed																	X		

Project 4 Statement of Work (GANT Chart)

Project 4: Data-driven Policy Recommendations																			
	**Year 1**				**Year 2**				**Year 3**				**Year 4**				**Core Involved**		
**Item**	**Q1**	**Q2**	**Q3**	**Q4**	**Q1**	**Q2**	**Q3**	**Q4**	**Q1**	**Q2**	**Q3**	**Q4**	**Q1**	**Q2**	**Q3**	**Q4**	**WDMC**	**ISC**	**CEC**
**Study Start-Up. Responsible Party: Project Manager, MPIs**																			
Meetings																			
Regulatory																			
**AIM 1 (Discover & Define): Characterize data and develop taxonomy.** *Responsible Party: MPIs, Core*																			
Access Data Sets																			
Codebook Development & Analysis																			
Meeting 1																			X
Create Data Summaries & Taxonomy																			
Meeting 2																			X
Deliverables: Data Summaries & Taxonomy																			
Publish Aim 1 Findings																	X	X	X
**AIM 2 (Develop): Stakeholder driven products and dissemination plan.** *Responsible Party: MPIs, Core*																			
Conduct Gap Analysis and Draft Content																			
Meeting 3																		X	X
Develop Products and Dissemination Plan																		X	X
Meeting 4																		X	X
Deliverables: Product Grid, Products and Dissemination Plan																		X	X
**AIM 3 (Validate): Products and dissemination plan.** *Responsible Party: MPIs, Core*																			
Compile content																			X
Meeting 5																		X	X
Finalize Product Grid, Products, and Dissemination Plan																			
Meeting 6																			
Deliverables: Final Product Grid, Products and Dissemination Plan																		X	X
Project Final Reports and Presentations																	X	X	X
