# Supplementary material for: Using Implementation Science to Improve Health Care Access and Quality for People With Traumatic Brain Injury–Related Morbidity (I-HEAL): Protocol for a Translational Multiproject Program Award
Source: JMIR Res Protoc. 2026 Mar 6;15:e79738. doi: 10.2196/79738 (PMC12995600; doi:10.2196/79738)
Supplement: Multimedia Appendix 3 [file resprot-v15-e79738-s003.docx]

**Transition Plan**

Our transition plan is based on the Department of Veterans Affairs QUERI (Quality Enhancement Research Initiative) Implementation Roadmap, a comprehensive, practical guide for health care practitioners and researchers to plan and deploy methods to support uptake of effective practices in routine care settings. The roadmap highlights a continuous learning cycle broadly classified into three phases. The Pre-Implementation Phase involves gathering data or information to create knowledge products. The Implementation Phase of the roadmap includes knowledge synthesis to inform or influence performance of the healthcare system to close the quality in care gap. The Sustainment Phase of the cycle includes building sustainability of knowledge, products, and interventions developed in the prior cycles. In a continuous learning healthcare system, performance benchmarks may reveal new or ongoing gaps that require new data to inform knowledge creation (Pre-Implementation) to inform the next generation of implementation efforts which is the focus of this proposed program of projects.

Data to Knowledge (Phase 1). Each project will inform development of resources (e.g. playbook, tool-kit, policy brief, grey literature, etc.) which will be managed and disseminated collectively on the online I-HEAL Toolshed (online dashboard and resource center). We will track knowledge products through the use of a strategic knowledge translation plan as outlined in the narrative. As an overall program (I-HEAL), we will leverage translational methods including: engagement (e.g., partnering with our intended end-users throughout); leveraging partnerships (e.g., Community Engagement Council); tailoring (e.g., using Human Centered Design); process modeling (e.g., adapting for workflow integration); and implementation planning. Engagement and leveraging partnerships are central to the I-HEAL transition planning. We will work closely with our Community Engagement Council Members which include key representatives from federal agencies, professional organizations, and persons with lived experience of TBI to maximize the success of our translation plan. Individual projects and the over-arching center will leverage integrated translation methods, to facilitate the following outcomes: (1) data to knowledge products (e.g., publications, presentations); (2) knowledge to performance outcomes (e.g., I-HEAL Professional Provider Toolshed); and (3) sustainment of efforts. Below, we describe our plan for achieving these goals in translation.

Individual Project Knowledge Production Process: We have proposed four unified projects prefaced on the QUERI (Quality Enhancement Research Initiative) Implementation Roadmap that will use tailored approaches to using translational methods to inform their intended outcomes and impact. The projects will use multiple translational methods including: engagement with the Community Engagement Council Members to develop recommendations for persons with TBI morbidity; leveraging partnerships with operational partners, family, V/SMs, and clinical providers from the overall center’s TBI Community will facilitate the creation evidence-informed products that are useful and impactful for clinical, system, and policy decision making; tailoring recommendations for persons with TBI morbidity; and prioritizing implementation planning as an aim to strategically support ongoing deployment through dissemination to key organizations and outlets identified by our Community Engagement Council with an eye towards future sustainment. Below, we specify key audiences, translation methods, and outcomes by project. Our partnerships with key stakeholders will maximize impact on care systems, advocacy efforts, and policy initiatives.

Project 1: Cognitive Nudge. Persons with TBI with cognitive disability experience gaps in quality of care due to poor engagement particularly without healthcare proxy support. The proposed study will develop and pilot a healthcare system intervention by using a nudge intervention in the electronic medical record system to cue providers to engage healthcare proxy or family members in healthcare appointments. Family member engagement has been found to be a facilitator to all types of healthcare for persons with TBI. Expected Outcomes include development of policy and procedure resources for implementation of the nudge intervention across healthcare systems. Engagement and user testing of these resources with healthcare system partners on the community engagement council and individual study engagement partners (e.g., Chief of Staff, PMR, Medicine, providers for persons with cognitive disability) enhance usability of products developed. Findings will be used in translational efforts to build the evidence base for deployment and adoption into routine systems of care.

Project 2: Enhancing Engagement in Behavioral Health Evidence-Based Treatments (EBT). Our work has shown that persons with TBI are not referred to or have difficulty engaging in guideline-endorsed behavioral health treatments due to TBI-related morbidity. Providers are mandated to adapt treatments for persons with disability but lack resources and education on how to adapt treatments. This project will identify best practices and further adapt evidence based behavioral health interventions (PTSD, Sleep, Chronic Pain, Depression) for persons with cognitive morbidity. Expected Outcomes include an online resource toolkit for providers and patients to maximize engagement in behavioral health treatments. Professional organizations partnering with the study team can help promote access to the toolkit and inform future implementation as demonstration of use as compliance with guideline-based care and incorporation into formal training programs with a diversity, equity, and inclusion focus.

Project 3: Improving management of maladaptive behaviors after TBI. Maladaptive behaviors after TBI are common and associated with a host of adverse patient and system outcomes. For example, maladaptive behaviors have been associated with denial to rehabilitation programming, delivery of non-evidence-based care, prolonged lengths of stay, and healthcare staff burnout and turnover. Rehabilitation team members highlight gaps in formal training and decreased confidence in management of maladaptive behaviors after TBI. This project proposes to adapt an evidence-based approach (STAR-VA for dementia) for healthcare teams in managing of maladaptive behaviors in the TBI inpatient rehabilitation setting. Expected outcomes include a manualized intervention with an implementation plan to promote adoption into programs. Engagement of partners representing varied stakeholders in managing maladaptive behaviors on inpatient rehabilitation units (nursing, psychology, therapy providers, PMR administration) and professional organizations (e.g., social work, rehabilitation psychology, physiatry) on the Community Engagement council maximize translation and adoption by commenting on the evolution of study products, identifying venues for provider education, and endorsement as meeting accreditation standards such as those by CARF International. Professional organizations may partner to disseminate formal training programs. The infrastructure of the American Congress of Rehabilitation Medicine Adult Training Institute could be utilized to offer training programs and accompanying manualized interventions (owned and distributed by that organization).

Project 4: Data driven policy recommendations for virtual healthcare for persons with TBI morbidity. Understanding the virtual healthcare needs and experiences for persons with TBI and chronic pain can inform accommodations needed to improve access across relevant access framework dimensions. This project proposes secondary analyses of existing qualitative interview data to examine facilitators and barriers to using virtual healthcare for persons with TBI and chronic pain. Expected Outcomes include development of data summaries which will drive development of recommendations with our Community Engagement Council to support policy recommendations for virtual healthcare for persons with TBI morbidity. These recommendations will be used to develop policy briefings and may also inform the initial development of parameters for a virtual health resource assessment for persons with TBI morbidity to determine appropriate approaches to virtual health resource use.

Knowledge to Performance (Phase 2). Diffusion and dissemination (year 3-8) of Knowledge Translation Products: The I-Heal team will partner with the community engagement council members to disseminate knowledge to potential users. The team will use a multifaceted dissemination strategy leveraging the resources of VA (VA TBI Knowledge Translation Center), DOD (Defense Health Agency TBI Center of Excellence), and NIDILRR (TBI Model System Knowledge Translation Center) to present and publish the results across individual projects. Community Engagement Council comprise members from these federal agencies and organizations for this purpose. These members include Ms. Linda Picon CCSP, VA TBI Liaison to DOD, who oversees the national Knowledge Translation program for TBI in VA rehabilitation settings. Dr. Kathryn Stout, Associate Director, Defense Health Agency TBI Center of Excellence, who oversees DOD’s comprehensive TBI educational products clearinghouse throughout the DOD. Lastly, have partnered with the NIDILRR-funded TBI Model System National Data and Statistical Center to develop an online dashboard and interim “Toolshed” for project deliverables. Many of the key personnel on this project serve on the NIDILRR Knowledge Translation Committee for the model Systems and oversee product development and deployment. Engagement partners are strategic in facilitating a long-term sustainable access point for project deliverables. Traditional dissemination will also include scientific meetings and journals, news outlets, and consumer-based media (e.g., web, trade journals, popular media, social media). We have partnered with TBI social media influencer, Kathryn Snedeker, CEO and founder of Pink Concussion, to create a social media campaign across Facebook, Twitter, and Instagram throughout the life of the project. Her content will be shared with partners from professional organizations and federal agencies to help reach multiple end user groups to create awareness of the Provider Toolshed and individual products. Subsequent dissemination grants will be submitted for the sustainment of the Professional Toolshed Dissemination Venue.

Sustainment (Phase 3). End user adoption, implementation, and institutionalization (year 9-12): The last phase of the QUERI Roadmap is system wide integration of the project interventions to improve access to high quality care for V/SM with TBI morbidity. The Professional and Policy Council members and Individual Project Engagement Partners will identify other key collaborators so that the interventions can be offered across health care systems in VA, DOD, and ultimately civilian healthcare. Implementing and sustaining evidence-based practices is challenging and complex, and may include a change champion in the organization who can address potential implementation challenges, piloting/trying the change in a particular patient care area of the organization, and using multidisciplinary implementation teams to assist in the practical aspects of embedding innovations into ongoing organizational processes. Health services and implementation science research is applicable to this stage of implementation and delivery to evaluate improvements in care and system-wide reduction healthcare disparities for persons with cognitive and other TBI morbidity.

To learn more about I-HEAL’s progress, visit the project website at ([https://ihealbrain.org](https://ihealbrain.org/)) and follow I-HEAL on social media:

X: @iheal_brain

Instagram: @iheal_brainhealth

Facebook: <https://facebook.com/iheal.brainhealth>

The study team is comprised of investigators with a successful health services, dissemination, and implementation science funding record at VA (Health Services Research and Development, QUERI), PCORI, and NIDILRR. Funding strategy and timeline for programmatic sustainment. Study investigators and engagement partners will advance the translational agenda for each project and examine implementation, patient, and system outcomes associated with each intervention designed to improve access. Potential funding sources include implementation science focused awards offered by VA, DOD, NIH, AHRQ, CDC, and PCORI. Study investigators have been successful in obtaining grants from these agencies (RR, JH, JB, JH, JC, KD, MS, BC). Long term sustainment goals for each project are identified in Table 4. Post-Award Timeline Toward Clinical Impact. The current proposal is prefaced on the QUERI Implementation Roadmap to optimize spread and impact. Therefore, we plan to secure subsequent funding in the following timeframe: 10/2026-all data analyzed and submitted for publication; 04/2027 – results prepared for grant submission; 06/2027-Grant submission for translational steps.

Table S1: Transition Plan

| **Projects and Potential Long-Term Sustainment Goals.** | |
| --- | --- |
| Cognitive Nudge (Systems Intervention) | **Targeting healthcare system adoption**   - Promotion as an objective criteria of patient safety, quality, and improvement for JCAHO standards across at-risk patient cohorts with neurologic cognitive morbidity. - Adoption in to Hospital Value-Based Purchasing Programs (i.e., CMS) |
| Provider Toolkit Adapting Behavioral Health EBTs (Provider Intervention) | **Targeting provider adoption**   - Utilization demonstrated as denoting Evidence Based Care - Educational curriculum for psychologists and a training program standard by the American Psychological Association Accreditation of Training Programs |
| Team Based Intervention for Managing Maladaptive Behaviors | **Targeting healthcare team adoption**   - Promotion as an objective-criteria for evidence-based management of maladaptive behaviors in accreditation programs for rehabilitation care (i.e., CARF). |
| Data Driven Policy for Telehealth Utilization in TBI | **Targeting healthcare policy**   - Recommendation adoption in VA and DOD |

Data Availability. Data will be stored and maintained in accordance with the VA/DOD approved Record Control Schedule. Final de-identified data sets underlying publications resulting from the proposed research will be available outside VA in an electronic format, through email upon request, after results are published. Data sets will be available for collaborators and other investigators upon request prior to publication (expected time period: 30 days post request). The extent of the data will be de-identified aggregate data only. De-identified data will be publicly shared through publications and scientific presentations at meetings with adequate detail to permit validation of results. Publications/presentations will be made available to the public through the National Library of Medicine PubMed Central website within one year upon request. The current project will not generate any identifiable human data.
