# Supplementary material for: Using Implementation Science to Improve Health Care Access and Quality for People With Traumatic Brain Injury–Related Morbidity (I-HEAL): Protocol for a Translational Multiproject Program Award
Source: JMIR Res Protoc. 2026 Mar 6;15:e79738. doi: 10.2196/79738 (PMC12995600; doi:10.2196/79738)
Supplement: Multimedia Appendix 4 [file resprot-v15-e79738-s004.docx]

**CBPR Approach**

Our proposal utilizes a Community Based Participatory Research (CBPR) approach. CBPR exists on a continuum of community engaged research and is the most recognized form of community engaged research aimed at addressing health outcomes. We employ it here as it has traditionally been used, to address disparities in healthcare by shifting the power in research teams to incorporate community voices and prioritize community solutions. Our team has been engaged in the CBPR partnership development process with those involved in this proposal for many years. We utilize both the research methods and the collaborative orientation to research embodied in CBPR principles. We have strong, bidirectional, and long-standing relationships between a diverse group of those with lived experience of TBI, professional partners, clinicians, and researchers. The ongoing work together has built trust and mutual respect over the years. Our collaborative team has been involved in the problem definition and prioritization, development of this proposal, and will continue to be involved through the implementation of study design and interpretation and dissemination of findings. We will use shared decision-making processes to ensure all aspects of the proposal are grounded in community principles, knowledge, and goals. We anticipate that this will lead to significant immediate, intermediate and long-term outcomes.

Figure S1: I-HEAL CBPR Conceptual Model

For this proposal, our ongoing process has led to the development of the Community Engagement Council (CEC) with three synergistic partner groups identified as key to the success of the proposal. The first group is the Policy and Professional Partners who will focus on dissemination and policy development. The second group is the Lived Experience Partners who bring their perspective on study impact for those with lived experience especially as this relates to facilitators and barriers to successful implementation and outcomes. The third group is a diverse group of partners who have been identified by each proposed project team to support the specific needs of each project.

We will achieve the I-HEAL objectives via recommended engagement principles of reciprocal relationships, co-learning, deep partnership, and transparency and honesty in all communication. We will operationalize and tailor these recommendations using strategies our team developed and utilized in a previous study. The strategies we utilized were developed as resource material for the Patient-Centered Outcomes Research Institute, particularly for teams with persons with TBI or other conditions that might affect cognition (paper in development). The strategies aim to improve the science and research outcomes by strengthening community and commitment and maximizing contributions of each partner. Specific engagement strategies employed in the current proposal include the following: 1) collaborative development of clear individual partner responsibilities as well as group responsibilities, 2) election of a co-chair from the Lived Experience Partner group to increase collaboration on meeting agendas and decision-making processes, 3) use of a consistent meeting structure to decrease the amount of new information to be processed at each meeting, 4) balance of team building and task oriented activities in meetings, 5) use of multiple methods of communication (e.g. text, phone, email) to provide reminders for meetings, meeting materials, and tasks, 6) provide technical support as needed to ensure capacity for meeting participation, 7) a mix of in-person and virtual meeting formats to increase opportunities for different kinds of connection and engagement, and 8) consistent respect for ideas and thoughts shared in meetings, allowing space for all interested to be able to participate. We will assess the success of these strategies using a study-specific brief survey of empowerment and engagement after each meeting of the CEC.

| Summary of CBPR Approach in Projects 1-4 | | | | | |
| --- | --- | --- | --- | --- | --- |
|  | End-user | Discover Aim 1 | Develop Aim 2 | Validate Aim 3 | Impact |
| Project 1 | Individual Engagement Partners | Engagement | Formative evaluation of toolkit products | Review of formative evaluation & input on finalization of toolkit & implementation content | Facilitate implementation |
|  | Professional Engagement Partners | Mixed methods surveys, focus groups |  | Semi-structured interviews, focus groups | Increased feasibility, acceptability, & adoption |
|  | Lived Experience Partners | Focus groups |  | Semi-structured interview | Increased acceptability |
| Project 2 | Individual Engagement Partners | Product grid development | Evaluation of product grid | Formative evaluation of toolkit | Increase feasibility & acceptability, increase skills & training, improve access to evidence base, facilitate implementation |
|  | Professional Engagement Partners | Environmental scan | X |  | Increase feasibility & acceptability, facilitate implementation |
|  | Lived Experience Partners | X | Evaluation of product grid |  | Increase access to care, increase acceptability |
| Project 3 | Individual Engagement Partners | Focus group recruitment, implementation readiness measure design | Focus group recruitment, implementation readiness measure design | Final approval of playbook | Pre-implementation buy-in |
|  | Professional Engagement Partners | Working meetings | Working meetings | Playbook pilot & Toolshed dissemination | Reduced burnout & injury, improved confidence in implementation |
|  | Lived Experience Partners | Focus groups | Focus groups | X | Improved access to evidence-based care |
| Project 4 | Individual Engagement Partners | Secondary analysis | Products & dissemination plan | Formative evaluation of products & dissemination plan | Pre-implementation buy-in, increase evidence base, readiness, adaptability, feasibility |
|  | Professional Engagement Partners |  |  |  |  |
|  | Lived Experience Partners |  |  |  | Pre-implementation buy-in, increase readiness |

***Study Inputs, Deliverables, and CBPR Effectiveness Measures***

Each study has identified specific inputs from the CEC that are outlined in the study descriptions. Specific study inputs from the CEC include study development to date, recruitment of focus group and survey participants, toolkit or playbook development and review, and identification of resources for existing behavioral health treatments for persons with TBI. Deliverables for the CEC, as outlined in the narrative description, include co-learning regarding research and community engagement, optimal recruitment via CEC-informed inclusive consent and recruitment processes and documents, meaningful data interpretation via collaborative analysis, CEC-informed dissemination materials, and successful dissemination of findings to inform policy and practice guidelines.

Effectiveness of our CBPR approach will be measured in several ways. We will utilize standard metrics of engagement success, including meeting attendance, self-reported empowerment within the group as measured by a brief, study-specific survey of partners administered after each meeting, and objective measures of engagement success pertinent to completion of each study. As outlined in the statement of work, each study will engage with the CEC at various points in their research. Reaching these engagement milestones will be documented and the success of the products determined by successful dissemination.

***Co-Learning***

We will utilize a co-learning model to ensure all team members are prepared for ongoing engagement in the research and in collaborative CBPR processes. At the first in-person meeting in Year 1, we will host trainings on research methods utilized in the four proposed studies and trainings on CBPR and strategies to achieve equitable participation and decision-making. Drs. Moore, Corrigan, and Radwan will work together with members of the CEC and research teams to coordinate and provide these trainings. All members of the teams have relevant expertise and experience to share. Guest speakers will also be sought when needed. Methods for inclusive learning will be used, including small group discussion, bidirectional opportunities for sharing ideas, and multiple methods for providing input.

In addition, Drs. Moore, Corrigan, and Radwan and other members of the CEC will engage in structured co-learning activities quarterly. We will focus on incorporation of innovative CBPR strategies, research methods relevant to the proposed studies, and engaged policy and dissemination strategies. These activities will include reading and discussion of relevant articles, popular media, or books suggested by members of the CEC on a rotating basis. Study team members will attend CEC meetings throughout the course of the study. In preparation for these meetings, study teams will be asked to review relevant CBPR processes being used by the CEC and prepare research presentation materials in a format that incorporates all of the levels of research knowledge in the CEC. They will provide their materials at least two weeks prior to their presentation to the CEC to allow CEC partners have time to review, prepare questions, and request additional information. A summary of all of the co-learning activities and key takeaways will be created by our social media and Communication Specialist for dissemination to all team members, investigators, and partners. As part of their roles in facilitating communication and co-learning between the CEC, the cores, and study teams, Drs. Moore, Corrigan, and Radwan and co-chair of the Lived Experience Partner group will provide structured and inclusive discussion of these materials at the beginning of each All-Hands Meeting.

***Democratization of Resources, Decision Making, and Authorship***

This proposal has been designed from the beginning to ensure democratization of resources, decision making and authorship. The CEC is one of three critical cores, including Dr. Moore (MPI) who serves also in the center leadership. A significant portion of the budget has been allocated to the CEC, including support or Drs. Moore, Corrigan, and Radwan’s time and equity-driven compensation for CEC partners.

Shared decision-making is a core CBPR principle. We will rely on trusted models of shared-decision making that include a focus on inclusion of relevant information, experience and preferences, deliberation, consensus building, and inclusion of choices and options. We will also utilize the inclusive structure of our CEC to ensure all voices are heard, which is particularly important for successful CBPR.

We will democratize authorship, guided by the principles of CBPR. The opportunity to participate as an author will be offered to all team members involved in each project. Since the CEC is substantively involved in all aspects of the proposal and ongoing work, those interested in participating in the writing of peer-reviewed manuscripts will be offered authorship. Other outreach and dissemination materials to communities will be authored by members of the CEC and study teams and credit given accordingly.

***Dissemination and Outreach Plan***

All findings will be disseminated widely to V/SM and to partners involved in TBI. We will produce a quarterly newsletter developed collaboratively with the CEC to be disseminated widely amongst our partners and relevant agencies. In addition, our Social-Media and Communication specialist will utilize her more than 40,000 social media followers in the TBI and V/SM community to disseminate quarterly study updates and exciting findings. The Policy and Professional Partners will advise on policy development strategies, and these will be incorporated into our policy and dissemination plan. Lastly, we have partnered with the TBI Model System Knowledge Translation Center as a major dissemination venue for long-term sustainment of products.
