# Supplementary material for: Using Implementation Science to Improve Health Care Access and Quality for People With Traumatic Brain Injury–Related Morbidity (I-HEAL): Protocol for a Translational Multiproject Program Award
Source: JMIR Res Protoc. 2026 Mar 6;15:e79738. doi: 10.2196/79738 (PMC12995600; doi:10.2196/79738)
Supplement: Multimedia Appendix 5 [file resprot-v15-e79738-s005.pdf]

Community Engagement Council Meeting Schedule				
**Communication Strategy**	**Purpose**	**Lived Experience Partners** *Purpose: Provide lived experience perspective to ensure successful outcomes*	**Policy and Professional Partners** *Purpose: Dissemination and policy development*	**Individual Study Partners** *Purpose: Advise on all aspects of individual studies and cores*
CEC Director		CR and MM	JC	MM
Kick-Off Meeting (Year 1 In-Person)	Investigator and stakeholder collaboration and agenda setting	X	X	X-Partial
Virtual Monthly LEP (YR 1-4)	Consumer input on planning, conduct, and dissemination	X		
Virtual Quarterly PPP (YR 1-4)	Professional input on planning, conduct, and dissemination		X	
Individual Project Specific Virtual Calls (YR 1-4)	End-user feedback on usability and relevance of products developed			X
Translation Meeting (Year 4 In-Person)	Investigator and stakeholder interpretation, dissemination, and translation planning	X	X	X-All
