# Supplementary material for: Using Implementation Science to Improve Health Care Access and Quality for People With Traumatic Brain Injury–Related Morbidity (I-HEAL): Protocol for a Translational Multiproject Program Award
Source: JMIR Res Protoc. 2026 Mar 6;15:e79738. doi: 10.2196/79738 (PMC12995600; doi:10.2196/79738)
Supplement: Multimedia Appendix 6 [file resprot-v15-e79738-s006.pdf]

Summary of Communication Strategies for Operational, Data Management, and Tracking Support			
**Communication Strategies**	**Purpose**	**Timing**	**Entities Involved**
**MEETINGS**			
**Videoconferences**			
All Hands Call	High-level review progress of study stand-up and conduct, analyses and dissemination	Monthly then Quarterly	MPIs, Cores, Project leads, Individual Project Co-Is
Executive Committee	Review progress of regulatory administrative, and financial issues	Weekly then Monthly	MPIs, PM
Steering Committee	Proactively address progress and situational awareness of core issues throughout the study	Biweekly then Monthly	MPIs, Cores, Project Leads
Individual Project Meetings	PI Meetings from CEC and WDMC to manage administrative and study issues	Weekly	CEC-MPI, WDMC-PI
Implementation Science Core and Website Data Management Core	Detailed review of progress on study conduct, analyses, and product generation	Weekly, then Monthly, then Quarterly	MPIs, BC, CR, NA, DO, MC, DM, JK
Community Engagement Council Professional and Policy Engagement Council	Professional audience feedback on methodology, implementation planning, and dissemination to professional organizations and federal agencies	Quarterly	Core lead and PPP members
Community Engagement Council Brain Injury Lived Experience Council	Consumer input on methodology, implementation planning, and dissemination to persons with TBI and their families	Monthly	JC, MM, CR, and LEP members
**In-Person Meetings**			
Kick-Off Meeting (Once)	Introduction to overall FPA, focused feedback sessions with individual studies (live focus groups)	Year 1	MPIs, Core Leads, PIs, CEC (LEP, PPP)
Final Translational Meeting (Once)	Review study findings, identify key stakeholder dissemination targets, discuss translation and implementation of study findings in live focus group sessions	Year 4	MPIs, Core Leads, PIs, CEC, (LEP, PPP, IEPs)
**AUTOMATED REPORTS**			
Enrollment, Missing Data	Missing Data Report (Each variable listed)		
**ELECTRONIC COMMUNICATION**			
Study Listserv	Private listserv for study communication	Year 1-5	MPIs, Cores, Project Leads, Individual Project Co-Is
Secure Website	Secure website with login via approved credentials for study staff		
Note: Mos=Month; MPI=Multi-Principal Investigators; Co-I=Co-Investigators; CEC=Community Engagement Council; ISC=Implementation Science Core; WDMC=Website and Data Management Core, PEP=Professional Engagement Partners, LEP=Lived Experience Partners; IEP=Individual Study Engagement Partners.			
