# Supplementary material for: Using Implementation Science to Improve Health Care Access and Quality for People With Traumatic Brain Injury–Related Morbidity (I-HEAL): Protocol for a Translational Multiproject Program Award
Source: JMIR Res Protoc. 2026 Mar 6;15:e79738. doi: 10.2196/79738 (PMC12995600; doi:10.2196/79738)
Supplement: Multimedia Appendix 7 [file resprot-v15-e79738-s007.docx]

Overall Project Leadership

The overall structure of the study team is reflected in Figure 1. The MPIs for this project represent expertise in military TBI and rehabilitation (Richardson), implementation science (Haun), and healthcare disparities (Moore) for persons with disability. The divergent expertise of the MPIs exemplify the team science approach in the proposed study and increasingly called for in translational science. Three cores WDMC, ISC, CEC will support the overall and individual projects. The individual project teams will be led by investigators across the TBIMS Research Network (see WDMC description) pairing senior investigators with early to mid-career investigators to foster development of the next generation of military TBI scientists in TBI and implementation science research. The investigators of this project have an extensive and successful work history on multicenter studies highlighting their ability to productively work together to complete projects.

Figure S1:


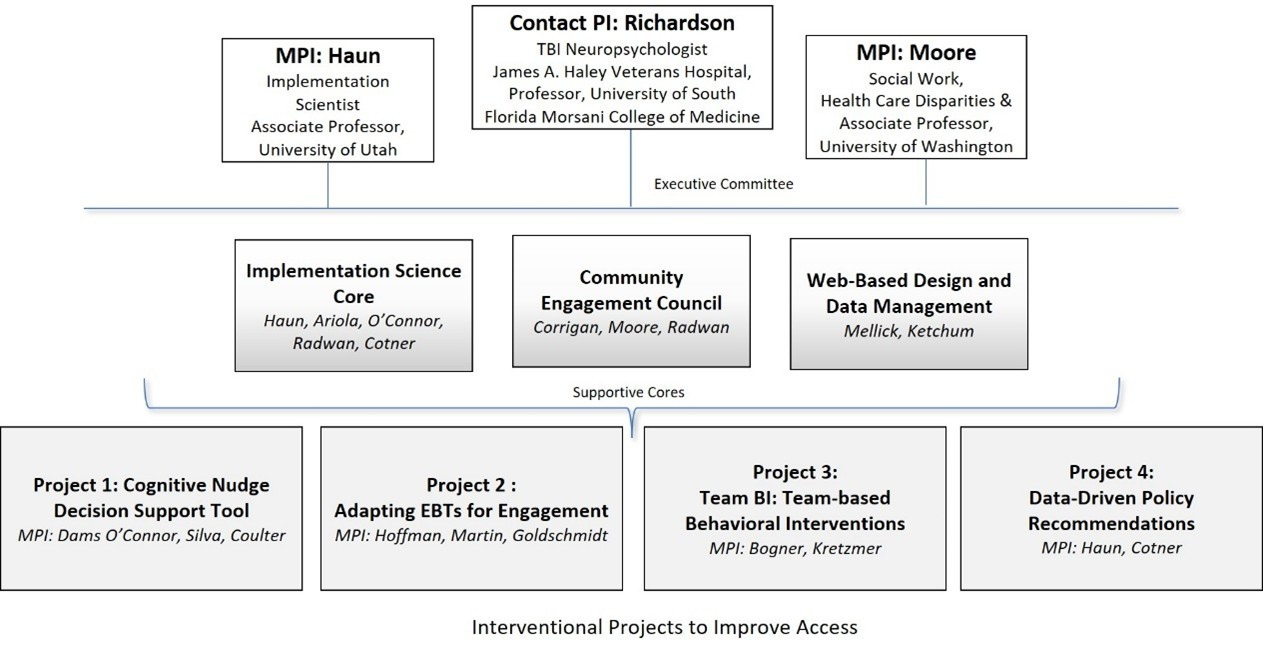


MPI Plan

Dr. Nakase-Richardson will serve as the contact PI and oversee all administrative aspects of the proposed projects. Dr. Haun will serve as the director of the ISC serving each project’s qualitative and implementation needs. Dr. Moore will oversee the operations of the CEC to effectively serve the needs of individual projects. Final dissemination and transition plans will be coordinated among the three MPIs.

Risa Nakase-Richardson, PhD, contact MPI (Military TBI Expert, 25% FTE). Dr. Nakase-Richardson is a joint faculty member serving as a Professor in the Department of Internal Medicine at the University of South Florida (Tampa, FL) and clinician investigator at the largest Polytrauma Rehabilitation Center (PRC) in the VA Polytrauma System of Care. Dr. Nakase-Richardson has successful leadership experience in leading large, multi-center projects in the VA and NIDILRR TBIMS Network. She has served as Principal Investigator (PI) on four multi-center studies funded by DOD, PCORI, NIDILRR, and VA QUERI leveraging the 21-center TBIMS Network. All projects successfully completed milestones on schedule or early. Her work has involved many of the collaborators on the proposed study highlighting excellent synergy in completing the proposed work. Dr. Nakase-Richardson has served in various capacities across 19 funded grants resulting in 121 peer-reviewed publications, knowledge translational products (3 clinical guidelines and position statements, [1-3] 1 manualized intervention of motivational interviewing to promote sleep apnea treatment adherence adapted for TBI), and patient health literacy products. According to Dimensions in VA, she is the number two most published author on military TBI.[4] Her extensive multi-center PI leadership and high-volume productivity demonstrate her expertise in serving as overall PI for this award. Dr. Nakase-Richardson also has extensive civilian and military TBI clinical and research expertise. Dr. Nakase-Richardson’s work has focused on both acute and chronic issues facing persons with TBI. Her clinical work has been funded and published on military TBI comorbidity topics including sleep disorders, chronic pain, and psychological health. She has published extensively on TBI related disability including cognitive, behavioral, and physical health challenges. Her work on military TBI has informed DOD and VA congressional reports on the long-term effects of military TBI and health system responses to their needs. [5] She has served as invited editor of three special issues in leading rehabilitation journals and professional magazines (*Journal of Head Trauma and Rehabilitation, Archives of Physical Medicine and Rehabilitation, and Brain Injury Professional*). Topics have included military TBI outcomes, TBI and sleep disorders, and severe TBI outcomes and healthcare utilization. Her recent work has highlighted barriers to accessing care thus inspiring this project’s focus and collaborators. Emphasis on Research Translation and Implementation Science: To facilitate translation of her own work, Dr. Nakase-Richardson has partnered with implementation scientists to increase this field of study in TBI. She and Dr. Jolie Haun (proposed study MPI) co-lead a VA QUERI implementation grant to characterize and study innovations in treating V/SMs in mild TBI residential programs. She is also a MPI on a grant examining facilitators and barriers to delivering chronic pain treatments for persons with TBI using a national cohort recruited through the TBIMS and professional communities. These and similar studies highlight her familiarity and expertise with common methods used in implementation science such as qualitative data collection, qualitative analyses, commonly used conceptual frameworks and theories, and unique dissemination strategies. Mentorship of the next generation of military scientists in the field of implementation scientists to promote research translation is a major focus of this proposal. For the past 23 years, Dr. Nakase-Richardson has mentored psychology postdoctoral fellows (neuropsychology, rehabilitation psychology), sleep medicine fellows, physical medicine and rehabilitation residents, nursing doctoral candidates, and health services specialists in both clinical and research capacities. Six of these students have received national or regional recognition with early career awards. She has previously served as a Director of Training of an APPCN-approved neuropsychology fellowship training program and is an active member of the University of South Florida’s Appointment, Promotion, and Tenure Committee in the Department of Internal Medicine. She currently mentors early and mid-career faculty in developing focused programs of research with three of her four junior faculty achieving independent funding. Collectively, these experiences support Dr. Nakase-Richardson’s expertise in serving as the I-HEAL contact PI.

Jolie Haun, PhD, EdS, Multi-Principal Investigator, MPI (Implementation Scientist; 20% FTE). Dr. Haun is joint faculty appointment serving as an Adjunct Associate Professor within the Division of Epidemiology, Department of Internal Medicine at the University of Utah. She has collaborated on 23 funded projects, including PI role on several research and operational quality improvement and implementation projects. Dr. Haun has 51 peer-reviewed publications, 4 book chapters, 3 white papers, 40 trade journal publications, 19 multi-media products, and 68 professional presentations focusing on implementation and outcomes research. Dr. Haun has successful leadership experience in leading large, enterprise-wide projects in the VA focused on implementation science. Dr. Haun has the experience and expertise required to participate in this proposed work as MPI and ISC lead. She has considerable experience using a CBPR approach to conduct Human Centered Design (HCD) projects with mixed methods to evaluate processes, and outcomes of implementation initiatives.[6] She has developed a body of published work in the redesign, implementation, and evaluation, and is nationally recognized for enterprise-wide implementation of information systems.[7] Dr. Haun has spent several years supporting evidence-based innovative research to support priorities for continuous learning health systems. Emphasis on research translation and implementation science. Dr. Haun’s entire program of research is implementation and translation focused, with several enterprise-wide projects supporting VA virtual healthcare resource implementation.[6, 8-10] More recently, Dr. Haun and Dr. Nakase-Richardson collaborated to converge Dr. Haun’s expertise in implementation science with Dr. Nakase-Richardson’s content expertise in TBI. Drs. Haun and Nakase-Richardson currently co-lead a VA QUERI implementation grant to characterize and study innovations in treating V/SMs in mild TBI residential programs. Dr. Haun’s funded program of research and quality improvement clearly substantiate her expertise with the proposed methods for each of the I-HEAL projects. For example, as it pertains to Project 1, Dr. Haun has practical experience in validating and implementing a clinical decision support tool (i.e., electronic health record nudge/flag) in the VA’s clinical health record, furthermore, she subsequently conducted a longitudinal cost study which was published as a seminal publication in the field of health literacy.[11-13] Relevant to Projects 2 and 3, Dr. Haun has considerable experience and expertise in content development, specifically educational and training materials, tool-kits, and playbooks.[14] Relevant to Project 4, Drs. Haun and Cotner have worked on and published on several qualitative HCD projects, with emphasis on virtual health resource utilization, including policy evaluation, and have the content and methodological expertise to successfully conduct and translate the Project 4 aims.[6, 15-17] Additionally, as the MPI of the newly funded StrAtegic PoLicy EvIdence-Based Evaluation CeNTer by the VA Health Services Research, & Development QUERI, she has the expertise and resources to support the successful transition of project findings to policy recommendations and to support appropriate access and use of virtual health resources by V/SM with TBI. Dr. Haun’s considerable experience with content development, including the use of multi-media to create training and education, tool-kits and playbooks, web-based and virtual health resource redesign, give her the experience and established approaches necessary to support launching I-HEAL into a position to produce project outputs and deliverables, as well as develop the I-HEAL toolshed to create a central resource hub for advancing implementation of evidence based practices to support access to care for persons with TBI.

Megan Moore, PhD, MSW, MPI, (Healthcare Disparities; 20% FTE). Dr. Moore is Sidney Miller Endowed Associate Professor in Direct Practice in the School of Social Work at the University of Washington. She is Core Faculty, Research Core Co-Director, and Director of the Injury-related Health Equity Across the Lifespan program and affiliate faculty at the Firearm Injury Prevention Research Center at the University of Washington Harborview Injury Prevention and Research Center, one of only eight Injury Control Research Centers in the country. Successful leadership in large center grants and multi-center projects in injury care: As a leading national expert in healthcare disparities in injury and TBI, Dr. Moore has published 50 peer-reviewed papers and has served as co-investigator (Co-I) or PI on 16 grants, including from NIH, PCORI, and Centers for Disease Control and Prevention (CDC). These grants, all related to healthcare disparities and most focused on injury-related disparities specifically, have involved mentored students, post-doctoral fellows, or junior faculty. She has experience serving in large center grant leadership as co-investigator and core co-director for the CDC-funded Harborview Injury Prevention and Research Center. In these roles, she directs the qualitative research methods, provides research mentorship for dozens of students, fellows, and faculty each year, and guides equity-driven scholarship and community engagement strategies. She is also co- investigator on a large, multi-center PCORI-funded study to improve access to care in the community and quality of life for persons with TBI and their caregivers; in this role she facilitates engagement with the patient and family partners and CBPR methods [18,19]. She has collaborated with many of the investigators on the current proposal on these and other projects. Her expertise in TBI disparities was sought by the NASEM to inform the national research agenda in TBI prevention and care. Given her productivity, track record of collaboration and leadership, and expertise in community engagement strategies and addressing TBI disparities, she is well prepared to serve as MPI on the current proposal. Dr. Moore’s clinical background is in social work with expertise in emergency department social work, critical care, and outpatient mental health services for persons with TBI, victims of violence, and other trauma. She brings this experience to her interdisciplinary research agenda. Her research is focused on health equity and improving health and mental health outcomes for persons who experience injury and violence. She grounds her work in the principles and philosophy of community engagement to achieve the best outcomes.[18,19] She engages with community at all stages of her work and has partnered closely with persons with TBI and their caregivers and professional partners on center-related activities and vision as well as on large-scale research projects. These collaborations have resulted in improved clinical practices and clinical care recommendations for persons with TBI as well as policy advocacy for injury prevention. Her focus on community engagement to address disparities in TBI for V/SM will be critical to the success of the current proposal. This proposal has been developed collaboratively with community partners who will continue to engage in all aspects of the research, aligned with the principles of community based participatory research. Dr. Moore will work closely with partners and investigators across the center to facilitate communication, engagement, and successful outcomes. Her track record of engaged and impactful research to address disparities, improve care and systems, and influence policy development has prepared her to serve as MPI on this proposal. Dr. Moore has mentored students at all levels of training, including undergraduate, graduate, post-doctoral, and junior faculty. In her roles at the University of Washington she has taught hundreds of students in the classroom and individually mentored dozens of masters and doctoral level students. In addition, she provides research consultation for an average of 20 fellows and junior faculty members each year at the University of Washington Harborview Injury Prevention and Research Center. She utilizes liberation models to equitably engage with students in a co-learning process. She utilizes these models in her research projects as well and will bring that practice to the current proposal. As an MPI, Dr. Moore will contribute to the overall success of the center as well as to the individual projects to address disparities in access to care for V/SM with TBI and ultimately improve outcomes for persons with TBI.

Additional Core Leadership

John D. Corrigan, PhD, CEC Chair is a board-certified rehabilitation psychologist and Professor in the Department of Physical Medicine and Rehabilitation at The Ohio State University. He is the director of the Ohio Valley Center for Brain Injury Prevention and Rehabilitation. Dr. Corrigan has extensive experience in grants administration and research, having been responsible for more than $30 million in grant funding for brain injury research. He has authored 195 peer-reviewed journal articles as well as 12 book chapters on TBI. He is the editor-in-chief of the Journal of Head Trauma Rehabilitation. Dr. Corrigan is a member of the Board of Directors of the Brain Injury Association of America. He has received local and national awards for his service and research in the field, including the William Fields Caveness Award from BIAA, the Robert L. Moody Prize and the American Congress of Rehabilitation Medicine’s Gold Key Award. Dr. Corrigan is currently leading stakeholder engagement efforts within the TBIMS Research Network with extensive collaboration with project investigators.

Chad Radwan, PhD, CEC Co-Chair is a health scientist at James A. Haley Veterans’ Hospital in Tampa, Florida. He is a trained applied anthropologist and has contributed his expertise as a qualitative methodologist to a variety of research studies and topics. For four years, he has led the Tampa Veterans Engagement Group, a dedicated group of Veteran and Veteran caregivers, in providing feedback on research studies. Many of the lived experience partners have worked with Dr. Radwan in this capacity. This dynamic group provides insights and key perspectives on a variety of research topics and at any phase of the study timeline (e.g., proposal submission, recruitment, dissemination, etc.). Recently, Dr. Radwan became PI of a study focused on understanding patients’ experiences with being moved in the hospital setting. He has also conducted extensive research on suicide prevention that includes posthumous interviews with Veteran family members to gain insight into possible motivating factors. Recently, he worked with colleagues to understand the effects of a positive airway pressure therapy intervention on Veterans with TBI. Dr. Radwan has been published in a variety of journals that include *Psychiatric Rehabilitation*, *Journal of Medical Internal Research*, *The Journal of Rehabilitation*, and *Economic Anthropology*, among others.

David C. Mellick, PhD, Director, I-HEAL WDMC, has a Doctorate degree in Clinical Sciences and is the Director of Research Operations at Craig Hospital. He is the PI of the TBIMS National Data and Statistical Center (NDSC) and a Co-PI of the TBIMS site at Craig Hospital responsible for the design, creation, and maintenance of the NDSC website, web database functions, and database management system that includes over thirty years of data on over 19,000 participants across a 21 center national network. Dr. Mellick has overseen operations of this system for the past 16 years. Dr. Mellick’s expertise has led to funding to advance this framework across multiple (>10) funded grants/contracts including the VA National TBI Registry including all 171 VA medical centers and separately the VA TBIMS program of research at the five VA PRCs. He has helped other investigators leverage the NIDILRR and VA TBIMS with data capture infrastructure to successfully complete multi center grants from PCORI, DOD, NIDILRR, and NIH. This includes successful collaborations with several proposed study investigators across these funded studies. His experience and expertise include innovative strategies for 1) customized web-based data collection solutions across public-private partnerships in research (VA, NIDILRR, academia), 2) diversifying participant modalities for engagement in research including customized email-based self-serve solutions and corresponding data quality assurance plans, and 3) novel solutions for data integration, harmonization across systems, and reporting via new data science tools. Dr. Mellick will be responsible for programming online surveys, developing an online dashboard documenting the overall and individual projects progress, and developing the beta version of the online I-HEAL Toolshed housing project deliverables.

Project Management

Role of the multi-center Project Manager (PM) in overall program and individual study conduct: Ms. Leah Phillips, MPH, Senior Project Manager, in several of Dr. Nakase-Richardson’s research studies, will serve as the lead PM for this study. She has served as the lead PM in four multi-center studies including three studies in the TBIMS network under Dr. Nakase-Richardson’s leadership. In this role, she has worked with over 95% of the proposed study team, centers, and population highlighting familiarity with administrative and scientific processes needed for the conduct of this award. Ms. Phillips will work closely with the MPIs and oversee execution of subcontracts, preparation and submission of the IRB application, co-development of the Manual of Operations (many elements of which already exist from the TBIMS infrastructure), implementation and organization of the communication strategies to facilitate standing up of individual cores, engagement communities, and individual studies.

Project 1

Three SMEs will co-lead this project. PI: Kristen Dams O’Connor, PhD, Clinical Neuropsychologist and Neurotraumatologist, SME: civilian and V/SM TBI, prognostic modeling and chronic TBI outcomes; comorbid disease management in chronic TBI; cognitive assessment and neurorehabilitation interventions; research translation; Co-PI: Marc A. Silva, PhD, Rehabilitation Neuropsychologist, SME: V/SM TBI, chronic rehabilitation needs; health and behavioral interventions that accommodate TBI impairments; cognitive and neurobehavioral assessment; pilot study and feasibility designs; Co-PI: Jill Coulter, SME: lived experience as a healthcare proxy for her spouse, a retired Navy Veteran who sustained a severe TBI in 2010; navigating military and civilian health systems, chronic care management, peer support in caregiving, disability advocacy. This team will be supported by two scientists: Co-I: Risa Nakase-Richardson, PhD, Neuropsychologist, SME: military TBI and health services research; implementation science in TBI care settings; Co-I: Bridget Cotner, PhD, Anthropologist, SME: qualitative methodology, mixed-method design; treatment barriers; access to care. Individual engagement partners include administrative partners (Tampa VA Chief of Staff, Medicine, PM&R, Psychology) and the Executive Director of the National Academy of Neuropsychology. See detailed descriptions in the Personnel Attachment.

Project 2

Jeanne M. Hoffman, PhD, ABPP(Rp), Professor, University of Washington, PI. Dr. Hoffman is a clinical psychologist and the Project Director of the University of Washington TBIMS and has extensive expertise in studying and working with individuals with TBI, including Veterans, and has led adaptation of behavioral interventions to address cognitive limitations associated with TBI with a focus on chronic pain and depression. She has led a large study of Veterans from the VA Puget Sound Health Care System using an adapted cognitive behavioral therapy intervention for chronic pain treatment and is currently involved in research on Veterans with mild TBI including treatments to manage cognitive limitations which interfere with access to healthcare. Aaron M. Martin, PhD, Co-I. Dr. Martin is an early-career clinical research psychologist at James A. Haley Veterans’ Hospital with a history of adapting behavioral EBTs for primary and specialty care clinical settings to address comorbid conditions. He has expertise in addressing sleep disorders in the context of chronic pain within outpatient polytrauma settings. His research focuses on understanding the nature of sleep and pain comorbidities in medically complex populations (e.g., TBI) with several publications to date.[20,21] Daniel Goldschmidt, Lived Experience Co-I. Mr. Goldschmidt is an individual with TBI resulting from a hiking accident in 2011. Since his severe TBI, he was unable to return to his profession due to difficulties with multi-tasking and attention but has returned to volunteer work with appropriate accommodations. He is committed to research and is especially interested and committed to helping others with TBI find providers who can modify their approach to work with an individual with cognitive difficulties. He has served as a lived experience stakeholder to several research teams. His role will be to provide overall lived experience guidance on this project to maximize utility and adoption of products. IEP are summarized below with detailed biographies in the Personnel Attachment.

Project 3

The project investigators have a substantive history of collaboration on multiple projects and publications. The project will be co-led by Jennifer Bogner, PhD, ABPP-Rp, FACRM and Tracy Kretzmer, PhD, ABPP-CN. Dr. Bogner’s relevant areas of experience include extensive research on measurement and treatment of agitation and other maladaptive behaviors following TBI, family involvement in rehabilitation, and the development and dissemination of implementation tools to improve inpatient rehabilitation evidence-based practice. Dr. Kretzmer has primarily worked as an inpatient neuropsychologist, with clinical expertise in treating individuals with brain injury. She has participated in research activities focused on the evaluation and development of evidence-based interventions for individuals with TBI and has co-led the preliminary study for this project with Dr. Nakase-Richardson. Risa Nakase-Richardson, PhD, FACRM, FNAN*,* Co-I, has published extensively on the neurobehavioral consequences of TBI resulting in two position statements defining clinical phenotypes at risk for maladaptive behaviors and competency standards for inpatient rehabilitation teams. She developed team-based learning programs for managing maladaptive behaviors after brain injury and has mentored early career professionals in collaborative projects examining patient and healthcare system impacts of maladaptive behaviors including access to care. The IEP were recruited to provide the multi-disciplinary perspective of the rehabilitation team, including rehabilitation team leaders/administrators (including physiatry); nurses; speech, physical and occupational therapy; and behavioral health providers.

Project 4

This project will be jointly led by Drs. Jolie Haun and Bridget Cotner, whom have an established history of working together on implementation focused studies (PI Haun, Co-I Cotner) using qualitative methods to support uptake and appropriate use of virtual health resources. Dr. Haun is a nationally recognized VA implementation scientist with expertise in virtual healthcare resource implementation. Her work has made several scientific contributions to the field relevant to virtual health research and policy development with at least 16 relevant publications,[6,8-11,15-17,22-29] and one book chapter,[7] with several Office of Connected Care-partnered projects which complement the proposed work. Dr. Haun’s stakeholder engaged work has supported: 1) implementation of a health literacy screening tool within the VA’s electronic health record to support clinical decision making (Unfunded dissertation);[11-13] 2) changes in policy, development of best practices, and human centered re-design of Secure Messaging (RRP 11-397);[8,26,28,29] 3) the human centered re-design of the VA (My HealtheVet) electronic health portal(RRP 12-495);[9,27] 4) operational planning for the integration, synchronization, and standardization of VA’s enterprise-wide virtual health resources (RRP 12-495);[9,10,27] 5) the first environmental scan, and collection of education and training available for VA’s virtual healthcare resources and examination of proactive integrated virtual health resource use among primary care teams within the VA pre- post COVID-19 (IIR 15-443);[6,15-17] 6) language development, field testing, and the human centered approach to the design of the VA’s emerging Veteran Delegation Tool to allow informal caregivers to coordinate care with the VA healthcare teams (OMAT 15019, manuscript in review); 7) field testing, protocol development and subsequent implementation of an organization-based national contract to facilitate electronic data collection of patient reported outcomes, outside of the VA firewall (PEC 16-354).[24]

Dr. Haun’s current work is focused on: 1) a stakeholder engaged project aimed to identify best practices in the proactive integrated use of VA’s enterprise-wide suite of virtual health resources (OCC-21-07; manuscript in development); 2) the enterprise-wide implementation of a virtually delivered pain management program (SP8E-PMTIA160; manuscript in review); 3) a national randomized controlled trial designed to test the effects of a partnered mobile and web based self-directed complementary and integrative health program for Veterans to manage pain and PTSD (IIR D2775-R); and 4) a VA Physical Medicine and Rehabilitation operations based partnered evaluation initiative aimed to support the implementation of a TBI Intensive Evaluation and Treatment Programs (PEC 21-129). Her work in this area of research has also been recognized and published by the former United States Secretary of VA.[7] Collectively Dr. Haun’s professional experiences using CBPR approaches stakeholder engaged approaches to conduct policy change and human-centered design work to promote the uptake and sustained use of virtual healthcare resources is uniquely aligned with the proposed project aims. Dr. Cotner is an anthropologist and early career investigator in TBI and healthcare access and inequities. She has served as a Co-I with the proposed PIs, Drs. Haun, Nakase-Richardson, and Co-I Hoffman, and has published with them.[6,9,15-17,34] As Co-I, she led the qualitative research data collection and analysis as part of the NIDILRR TBI and chronic pain study that collected the interview data to be analyzed in this study. Her knowledge of the qualitative data will greatly facilitate the proposed secondary data analysis to address Aim 4.1 and inform Aims 4.2 and 4.3. Dr. Cotner also has a history of engaging partners into the research process to inform research activities and knowledge translation through her two Health Services, Research, & Development small project awards (PI Cotner, Co-Is Nakase-Richardson and Hoffman) and as Co-I on DOD-funded research.[30-33] This experience will facilitate the engagement partners throughout this study. Additionally, Dr. Cotner has worked and co-authored on Dr. Haun’s previous implementation focused virtual health resource projects.

Drs. Haun and Cotner have worked on multiple mixed-methods projects leveraging CBPR approaches for the purposes of guiding stakeholder engagement and HCD methods to inform policy change and implementation of virtual health resources. Their combined expertise and use of an established published protocol[6,9] inform the project aims, methods, and plan for stakeholder engagement to maximize practical and stakeholder driven deliverables and dissemination.

Additional Personnel include two anthropologists who are trained in qualitative methods from the ISC. They will work directly with Dr. Cotner to conduct the secondary data analyses (Aims 4.1); and led by Dr. Haun will inform product development and implementation (Aims 4.2 and 4.3) as part of the ISC team.

References

1. Giacino JT, Katz DI, Schiff ND, et al. Practice guideline update recommendation summary: Disorders of consciousness: Report of the guideline development, dissemination, and implementation subcommittee of the American Academy of Neurology; the American Congress of Rehabilitation Medicine; and the National Institute on Disability, Independent Living, and Rehabilitation Research. *Neurology*. 91(10):450-460. doi: 10.1212/WNL.0000000000005926.
2. Giacino JT, Whyte J, Nakase-Richardson R, et al. Minimum competency recommendations for programs that provide rehabilitation services for persons with disorders of consciousness: A position statement of the American Congress of Rehabilitation Medicine; and the National Institute on Disability, Independent Living, and Rehabilitation Research Traumatic Brain Injury Model Systems. *Arch Phys Med Rehabil*. 2020;101(6):1072-1089. doi: 10.1016/j.apmr.2020.01.013.
3. Sherer M, Katz DI, Bodien YG, et al. Post-traumatic confusional state: A case definition and diagnostic criteria. *Arch Phys Med Rehabil*. 2020;101(11):2041-2050. doi:10.1016/j.apmr.2020.06.021
4. U.S. Department of Veterans Affairs. Dimensions for VA. Updated November 11, 2022. Accessed November 21, 2022. <https://www.hsrd.research.va.gov/for_researchers/dimensions.cfm>
5. Military Health System and Defense Health Agency. TBICoE Research. Accessed November 21, 2022. <https://www.health.mil/Military-Health-Topics/Centers-of-Excellence/Traumatic-Brain-Injury-Center-of-Excellence/Research>.
6. Haun J, Chavez M, Hathaway W, Antinori N, Melilo C, Cotner BA, McMahon-Grenz J, Zilka B, Patel-Teague S, Messina W, Nazi K. Virtual medical modality implementation strategies for patient-aligned care teams to promote Veteran-centered care: Protocol for a mixed-methods study. *JMIR Res Protoc*. 2018;7(8):e11262. doi: 10.2196/11262.
7. Haun, JN, Chavez M, Hathaway W, Antinori, N, Vetter, B, Miller, B, Martin, T, Ruggerie, T Kendziora, L. Nazi, K. Promoting proactive use of secure messaging using promising practices. In: Shulkin D, Elnahal S, Maddock E, Shaheen M, eds. *Best Care Everywhere* US Department of Affairs. 2017:6-7.
8. Haun JN, Hathaway W, Chavez M, et al. Clinical practice informs secure messaging benefits and best practices. *Appl Clin Inform*. 2017;8(4):1003-1011. doi:10.4338/ACI-2017-05-RA-0088
9. Haun JN, Chavez M, Nazi K, et al. Veterans' preferences for exchanging information using Veterans Affairs health information technologies: Focus group results and modeling simulations. *J Med Internet Res*. 2017;19(10):e359. doi:10.2196/jmir.8614
10. Haun JN, Chavez M, Nazi KM, Antinori N. Developing a health information technology systems matrix: A qualitative participatory approach. *J Med Internet Res*. 2016;18(10):e266. doi: 10.2196/jmir.6499.
11. Haun JN, Patel NR, French DD, Campbell RR, Bradham DD, Lapcevic WA. Association between health literacy and medical care costs in an integrated healthcare system: A regional population based study. *BMC Health Serv Res*. 2015;15:249. doi: 10.1186/s12913-015-0887-z
12. Haun J, Luther S, Dodd V, Donaldson P. Measurement variation across health literacy assessments: Implications for assessment selection in research and practice. *J Health Commun*. 2012;17 Suppl 3:141-159. doi:10.1080/10810730.2012.712615
13. Haun J, Noland-Dodd V, Varnes J, Graham-Pole J, Rienzo B, Donaldson P. Testing the BRIEF health literacy screening tool. *Fed Pract*. 2009;26:12:24-31. https://cdn.mdedge.com/files/s3fs-public/Document/September-2017/026120024.pdf
14. Haun J, Melillo C, Benzinger R. On Your Mark, Get Set, Go--with a Playbook! Instructional Course presented at: American Congress of Rehabilitation Medicine; November 9, 2022; Chicago, IL.
15. Haun JN, Panaite V, Cotner BA, et al. Primary care virtual resource use prior and post COVID-19 pandemic onset. *BMC Health Serv Res*. 2022;22(1):1370. doi: 10.1186/s12913-022-08790-w
16. Haun JN, Panaite V, Cotner BA, et al. Provider reported value and use of virtual resources in extended primary care prior to and during COVID-19. *BMC Health Serv Res*. 2022;22:1353. DOI: [10.1186/s12913-022-08752-2](https://doi.org/10.1186/s12913-022-08752-2)
17. Haun JN, Cotner BA, Melillo C, et al. Informing Proactive integrated virtual healthcare resource use in primary care. *BMC Health Serv Res.* 2021;21(1):802. doi: 10.1186/s12913-021-06783-9.
18. Schmittdiel JA, Grumbach K, Selby JV. System-based participatory research in health care: An approach for sustainable translational research and quality improvement. *Ann Fam Med*. 2010;8(3):256-259. doi:10.1370/afm.1117
19. Israel BA, Eng E, Schulz AJ, Parker EA, eds. *Methods for Community-Based Participatory Research for Health*. 2nd ed. Jossey-Bass; 2012:736.
20. Martin AM, Pinto SM, Tang X, et al. Associations between early sleep-disordered breathing following moderate-to severe (TBI) and long-term chronic pain status: A TBI Model Systems study. *J Clin Sleep Med*. 2023; 19(1):135-143. doi:10.5664/jcsm.10278.
21. Martin AM, Almeida EJ, Starosta AJ, et al. The impact of opioid medications on sleep architecture and nocturnal respiration during acute recovery from moderate to severe traumatic brain injury. *J Head Trauma Rehabil*. 2021;36(5):374-387. doi: 10.1097/HTR.0000000000000727
22. Haun JN, Alman AC, Jean-Baptiste E, Melillo C, McMahon-Grenz J, Paykel JM. Delivery of complementary and integrative health using virtual health resources: A scoping review. *J Integr Complement Med*. 2022;28(11):851-861. doi:10.1089/jicm.2021.0458
23. Haun JN, Paykel J, Melillo C. Transforming health and resiliency through integration of values-based experiences: Implementation of an electronic evidence-based Whole Health Clinical Program. *JMIR Form Res*. 2021;5(6):e26030. doi: 10.2196/26030
24. Haun JN, Alman AC, Melillo C, et al. Using electronic data collection platforms to assess complementary and integrative health patient-reported outcomes: Feasibility project. *JMIR Med Inform*. 2020;8(6):e15609. doi:10.2196/15609
25. Bradley SE, Haun JN, Powell-Cope G, Haire S, Belanger HG. Qualitative assessment of the use of a smart phone application to manage post-concussion symptoms in Veterans with traumatic brain injury. *Brain Inj*. 2020;34:8:1031-1038. DOI: [10.1080/02699052.2020.1771770](https://doi.org/10.1080/02699052.2020.1771770)
26. Haun JN, Patel NR, Lind JD, Antinori N. Large-scale survey findings inform patients' experiences in using secure messaging to engage in patient-provider communication and self-care management: A quantitative assessment. *J Med Internet Res*. 2015;17(12):e282. doi:10.2196/jmir.5152
27. Haun JN, Nazi KM, Chavez M, et al. A participatory approach to designing and enhancing integrated health information technology systems for Veterans: Protocol. *JMIR Res Protoc*. 2015;4(1):e28. doi: 10.2196/resprot.3815.
28. Haun JN, Lind JD, Shimada SL, et al. Evaluating user experiences of the secure messaging tool on the Veterans Affairs' patient portal system. *J Med Internet Res*. 2014;16(3):e75. doi:10.2196/jmir.2976
29. Haun JN, Lind JD, Shimada SL, Simon SR. Evaluating secure messaging from the Veteran perspective: Informing the Adoption and sustained use of a patient-driven communication platform. *Ann Anthropol Pract*. 2013;37(2):57-74. [**https://doi.org/10.1111/napa.12029**](https://doi.org/10.1111/napa.12029)
30. Fyffe DC, Cotner BA, Tobin P, et al. Multi-phase Veteran engagement to develop a spinal cord injury employment survey.*JGIM.*2023;17(1):e3-e4. doi: 10.1353/cpr.2023.0001
31. Chavez M, Cotner BA, Hathaway W. Building rapport during applied research recruitment. *Anthropology News*. 2017;58(3):e271-e275. https://doi.org/10.1111/AN.427
32. Wallace T, Cotner, BA, Klyce D, Gore R, Hodge A. Patient goal directed care in brain injury rehabilitation. Symposium presented at: 99th annual meeting of the American Congress for Rehabilitation Medicine, November 8-11, 2022, Chicago, IL.
33. Cotner BA, Ottomanelli L, Fyffe D, O’Neill J. Stakeholder informed employment resources for Veterans living with spinal cord injury. Symposium abstract accepted at: 99th annual meeting of the American Congress for Rehabilitation Medicine, November 8-11, 2022, Chicago, IL.
34. Cotner BA, Nakase-Richardson R, O’Connor DR, et al. Barriers and facilitators to accessing rehabilitation health care: A Veterans Affairs Traumatic Brain Injury Model Systems qualitative study. *Arch Phys Med Rehabil*. 2022;S0003-9993(22)01659-8. doi: 10.1016/j.apmr.2022.09.020
