# Supplementary material for: Using Implementation Science to Improve Health Care Access and Quality for People With Traumatic Brain Injury–Related Morbidity (I-HEAL): Protocol for a Translational Multiproject Program Award
Source: JMIR Res Protoc. 2026 Mar 6;15:e79738. doi: 10.2196/79738 (PMC12995600; doi:10.2196/79738)
Supplement: Multimedia Appendix 8 [file resprot-v15-e79738-s008.pdf]

Project 1 Cognitive Nudge Clinical Decision Support Tool Toolkit Development			
Meeting Series	Purpose/Task	Participants	Timeframe
1-2	Review triangulation of findings from Aim 1 and identify needs and products for toolkit development	Project 1 MPIs, Co-Is, and CEC	Mos 25-27
3	Outline product suggestions and prioritize content	Project 1 IEPs	Mos 28-30
4-6	Adapt existing products from other nudge interventions and develop new products necessary for toolkit and implementation plan	Project 1 MPIs and Co-Is	Mos 26-34
7-9	Conduct formative evaluation of toolkit content and implementation plan	ISC, CEC, and IEPs	Mos 35-37
Mos=Study Month, MPI=Multi-Principal Investigators, Co-I=Co-Investigators, IEP=Individual Engagement Partners, CEC=Community Engagement Council comprised of two groups including lived experience partners and professional partners.			
