# Supplementary material for: Using Implementation Science to Improve Health Care Access and Quality for People With Traumatic Brain Injury–Related Morbidity (I-HEAL): Protocol for a Translational Multiproject Program Award
Source: JMIR Res Protoc. 2026 Mar 6;15:e79738. doi: 10.2196/79738 (PMC12995600; doi:10.2196/79738)
Supplement: Multimedia Appendix 9 [file resprot-v15-e79738-s009.pdf]

Project 2 Individual Engagement Partner Areas of Subject Matter Expertise	
**Areas of Expertise**	**IEP or Study Investigator**
**Chronic Pain and Headache**	Curtis Takagishi, Ph.D., Headache Treatment Clinic, James A. Haley VAMC
	Katie Pagulayan, Ph.D., U. Washington
**Mental Health (PTSD, Depression)**	Shannon Miles, Ph.D., PTSD Treatment Program, James A. Haley VAMC
	Luzimar Vega, Ph.D., Polytrauma Network Site Outpatient Program for TBI, James A. Haley VAMC
**Behavioral Health Interventions for Sleep Disorders**	Erica Healey, Psy.D., SCI Rehabilitation, James A. Haley VAMC
	Lara Wittine, M.D., Sleep Medicine, James A. Haley VAMC
**Additional participants with subject matter expertise involved in the FPA (Nakase-Richardson, Corrigan, Bogner) will provide supplemental engagement as rehabilitation and neuropsychology subject matter experts.**	
