# Supplementary material for: Using Implementation Science to Improve Health Care Access and Quality for People With Traumatic Brain Injury–Related Morbidity (I-HEAL): Protocol for a Translational Multiproject Program Award
Source: JMIR Res Protoc. 2026 Mar 6;15:e79738. doi: 10.2196/79738 (PMC12995600; doi:10.2196/79738)
Supplement: Peer Review Report 1 [file resprot-v15-e79738-s010.pdf]

Department of Defense
U.S. Army Medical Research and Development Command
Congressionally Directed Medical Research Programs
Fiscal Year 2022 Traumatic Brain Injury and Psychological Health Research Program
Focused Program Award - Human Subjects
Peer Review Summary Statement

CDMRP Log Number: TP220152
Grants.gov ID Number: GRANT13765226
Review Panel: Focused Program - Healthcare Access
Discussion Period: 02/09/2023-02/09/2023

Project Duration: 48 months
Budget Requested: $6,357,910
Direct Costs: $4,999,809
Indirect Costs: $1,358,101

Title: Improving Health Care Access and Engagement for Veterans and Service Members with TBI Morbidity
Principal Investigator: Risa Richardson
Performing Organization: VA Medical Center, James A. Haley, Tampa, FL
Contracting Organization: Tampa VA Research and Education Foundation, Inc

	Average Score	Standard Deviation
**Overall Evaluation**<br>*Rating Scale: 1.0 (highest merit) to 5.0 (lowest merit)*	1.7<br>**(Excellent)**	0.3

Procurement Sensitive Document
Do not copy or distribute without CDMRP written permission.
Prepared by SRA International, Inc., A GDIT Company, a support contractor to CDMRP

	Application	Overall Program	Project 1	Project 2	Project 3	Project 4
**Overall Evaluation**<br>Rating Scale: 1.0 (highest merit) to 5.0 (lowest merit)	1.7 (0.3)	1.5 (0.1)	1.7 (0.3)	1.8 (0.1)	1.9 (0.3)	1.5 (0.1)
**Evaluation Criteria**<br>Rating Scale: 10.0 (highest merit) to 1.0 (lowest merit)						
**Overall Program**		8.8				
**Leadership**		9.5				
**Implementation Plan**		9.0				
**Overall Impact and Relevance to Military Health**		9.0				
**Research Strategy and Feasibility**			8.0	8.3	8.8	9.3
**Impact and Relevance to Military Health**			8.8	7.6	7.6	8.6
**Regulatory Strategy and Transition Plan**			8.0	7.2	8.2	8.2
**Ethical Considerations**			9.0	N/A	8.4	N/A
**Statistical Plan**			7.6	7.2	6.6	8.4
**Personnel**			9.5	8.0	9.8	9.8
**Community-Based Participatory Research**			9.8	N/A	9.5	N/A
**Data and Research Resources Sharing Plan**			8.8	9.0	9.0	8.8

Procurement Sensitive Document
Do not copy or distribute without CDMRP written permission.

Improving Health Care Access and Engagement for Veterans and Service Members With TBI Morbidity

OVERVIEW

The Principal Investigator (PI) of this application proposes to address key knowledge gaps to improve access and engagement in high-quality, evidence-based health care services for veterans and service members with traumatic brain injury (TBI) morbidity. Four research projects are included: (1) Health Care Disparity Risk Reduction via Innovative Medical Records Flag for Post-TBI Cognitive Impairment: A Cognitive Nudge Decision Support Tool; (2) Provider Toolkit for Accommodating Cognitively Impaired Persons in Evidence Based Treatments; (3) Empowering Teams to Implement Evidence-Based Behavioral Interventions (TeamBI); and (4) Data-Driven Policy Recommendations for Virtual Health Care for Persons With TBI Morbidity.

	**Average Score**	**Standard Deviation**
**Overall Evaluation**<br>*Rating Scale: 1.0 (highest merit) to 5.0 (lowest merit)*	1.5<br>**(Outstanding)**	0.1
**Criteria**<br>*Rating Scale: 10 (highest merit) to 1 (lowest merit)*	**Average Score**	
**Overall Program**	8.8	
**Leadership**	9.5	
**Implementation Plan**	9.0	
**Overall Impact and Relevance to Military Health**	9.0	

SCORED CRITERIA

Overall Program
Average Score: 8.8

Scientist Reviewer A

Strengths: The overarching goal of the Improving Health Care Engagement and Access to Optimize Long-Term Outcomes (I-HEAL) program is to improve access to and accessibility of health care to individuals with TBI in general and to those with cognitive and behavioral impairments specifically. The research projects are not dependent on each other but complement each other well.

Weaknesses: No weaknesses were noted.

Scientist Reviewer B

Strengths: The application outlines a set of projects that will address critical challenges in treatment of people with TBI at multiple levels (eg, system-level, provider-level, policy-level). The proposed projects will act on the health care experiences of people with TBI. The project addresses subarea 3a: treat. The projects are not interdependent, but are interrelated and synergistic and leverage an impressive multidisciplinary research program devised by an extensive team.

Weaknesses: The projects are each impactful on their own, but as a whole there are some potential gaps in coverage of the question of improving access to care for veterans and service members with TBI. For example, Project 1 creates nudges to engage proxies in care, but may be missing other methods for improving health care experiences that are focused on (nonpsychological) providers. Project 4 focuses only on developing policy recommendations for virtual health care and may be missing out on impactful recommendations for in-person care.

Leadership
Average Score: 9.5

Scientist Reviewer A

The PI, Risa Richardson, received a PhD in clinical psychology from West Virginia University in 2000 and is professor, clinician investigator, and director of sleep medicine research in the Morsani College of Medicine, University of South Florida and senior research director, Defense Health Agency TBI Center of Excellence at James A. Haley Veterans Hospital.

Strengths: Dr Richardson has led many multicenter projects. She is PI of a TBI Model System and has a strong history of collaboration with the program team and has a long history of mentoring junior investigators. Her past experience will likely contribute to her success in leading this project. The PI will devote 25% effort to the project. Via letters of support the PI clearly has institutional and community support for the project. The qualifications of the team are exceptional and will likely result in a robust synergistic collaboration.

Weaknesses: No weaknesses were noted.

Scientist Reviewer B

Strengths: The team that has been assembled is composed of both leaders in all relevant domains and promising early career scientists, as well as key stakeholders. This is an impressive and experienced team led by a PI with experience leading large, focused projects that have been completed early or on time. The PI will be at 25% level of effort on the proposed program.

Weaknesses: No weaknesses were noted.

Implementation Plan
Average Score: 9.0

Scientist Reviewer A

Strengths: Detailed implementation plans and timelines are provided. Knowledge and resources are clearly described, and technological innovations are planned. The various cores are well described and leveraged throughout the entire program. Community-based participatory research (CBPR) and stakeholder involvement are integral to the project. There are clear plans to evaluate progress throughout each project. The overall management plan is well described and has checks and balances throughout the proposed program. There is a data management core based on the TBI Model Systems, and their plan for sharing information across the project sites is clear. There is a plan to utilize and build upon proven TBI Model Systems standard operating procedures.

Weaknesses: No weaknesses were noted.

Scientist Reviewer B

Strengths: Implementation plans for each project are well-detailed, identify critical milestones, and propose feasible plans to achieve these milestones. Several of the projects are focused on preparing products to be implemented in a future study after the completion of this proposed award. Stakeholders are engaged at each level of the proposed program and will contribute meaningfully to the final products.

Weaknesses: The overall management plan is described only briefly and appears to rely on the PI's (admittedly considerable) prior experience in managing large teams.

Overall Impact and Relevance to Military Health
Average Score: 9.0

Scientist Reviewer A

Strengths: All outcomes and outputs are directly related to veterans and service members with TBI. Collectively, these studies have the ability to not only impact individuals with TBI but their caregivers and providers as well. The proposed projects have the capacity to improve patient/provider communication, treatments, and policies. Each project proposes potential pitfalls and provides suggestions for ameliorating them.

Weaknesses: No weaknesses were noted.

Scientist Reviewer B

Strengths: If successful, the overall program will have meaningful impacts on the health care experiences of veterans and service members with TBI by addressing critical issues at multiple levels (eg, provider, policy). If the knowledge products created by this study are successfully implemented and disseminated, they will have a substantial impact. The community engagement proposed by the project makes it possible for the relevant stakeholders to contribute to the direction of the project so that it successfully responds to the health care needs of veterans and service members.

Weaknesses: No weaknesses were noted.

Consumer Reviewer

Strengths: The proposed overall program is addressing a gap in research translation identified by the National Academies of Science, Engineering, and Medicine. The project is focused on the known disparity in health outcomes for service members and veterans with TBI. With the structure of the project, there is an emphasis on developing knowledge and approaches as well as on the practicalities of implementing within and across complex health care systems. The project is incorporating input from service members, veterans, and family members throughout implementation.

Weaknesses: The section on potential challenges and barriers is limited to 1 identified challenge/question per project and does not outline the range of potential challenges the overall program might encounter.

Procurement Sensitive Document
Do not copy or distribute without CDMRP written permission.

UNSCORED CRITERIA

Budget

Scientist Reviewer A

Total direct costs do not exceed $5 million. The budget is appropriate for the proposed research.

Scientist Reviewer B

The budget appears to be within the allowable range and appropriate for the proposed research.

Application Presentation

Scientist Reviewer A

The writing was clear and concise and directly influenced the review.

Scientist Reviewer B

The writing and presentation are fairly clear, although some grammatical/typing errors and missing information (eg, “fill in here” type statements) do make the application slightly difficult to follow at points.

Project 1: Health Care Disparity Risk Reduction via Innovative Medical Records Flag for Post-TBI Cognitive Impairment: A Cognitive Nudge Decision Support Tool

OVERVIEW

The leaders of this project propose to develop an electronic medical record (EMR) flag (ie, "Cognitive Nudge") to cue providers to include health care proxies during health encounters when recommended by TBI specialists. The specific aims are to (1) discover and engage health care providers, persons with TBI, and their families to identify content, processes, and procedures to develop a cognitive nudge clinical support tool; (2) define and develop and codesign a cognitive nudge clinical decision support tool with health care providers, persons with TBI, and their families; and (3) validate, implement a pilot, and evaluate outcome of the cognitive nudge at a major VA medical and polytrauma rehabilitation center.

	**Average Score**	**Standard Deviation**
**Overall Evaluation**<br>*Rating Scale: 1.0 (highest merit) to 5.0 (lowest merit)*	1.7<br>**(Excellent)**	0.3
**Criteria**<br>*Rating Scale: 10 (highest merit) to 1 (lowest merit)*	**Average Score**	
**Research Strategy and Feasibility**	8.0	
**Impact and Relevance to Military Health**	8.8	
**Regulatory Strategy and Transition Plan**	8.0	
**Ethical Considerations**	9.0	
**Statistical Plan**	7.6	
**Personnel**	9.5	
**Community-Based Participatory Research**	9.8	
**Data and Research Resources Sharing Plan**	8.8	

SCORED CRITERIA

Research Strategy and Feasibility
Average Score: 8.0

Scientist Reviewer A

The plan is to create a stakeholder driven nudge in the EMR to let providers know when an individual with a TBI may need caregiver assistance during a medical appointment.

Strengths: The scientific rationale and literature review provide the basis for the study aims and hypotheses. The proposed project is directly relevant to the FPA mechanism. The purpose and objectives of the study and the specific aims are well described and aligned with the tasks in the Statement of Work. The design of the study will achieve the specific aims. Based on the letters of support and past experience, the study team seems to have access to the needed samples/populations. The proposal identifies potential pitfalls and solutions. The proposed project appears to be feasible and achievable within the specified time frame. Various and appropriate stakeholders are included.

Procurement Sensitive Document
Do not copy or distribute without CDMRP written permission.

Weaknesses: There is minimal mention of inclusion/exclusion of women or minorities or whether sex/gender will be addressed in any particular way in this project.

Scientist Reviewer B

Strengths: The applicants have done a very good job in presenting a scientific rationale that is based on a review of the literature including their own published studies to support the development of the proposed project. The objectives, aims, and hypotheses formulated in the proposed project are based on a thorough and critical review and understanding of the scientific literature that forms the basis for the proposed project. The objectives, aims, and hypotheses are well described and aligned with the tasks in the Statement of Work.

The proposed research and findings are applicable to focus 3, focus area 3c and secondary focus area 3a. The experimental design, methods, and analyses are very relevant to the completion of the different aims of the project. The applicants plan to use the Consolidated Framework Interview Guide to identify potential problems and present approaches to mitigate any risks that are identified.

Weaknesses: The timeline and the recruitment process for the proposed project are somewhat overambitious.

Impact and Relevance to Military Health
Average Score: 8.8

Scientist Reviewer A

Strengths: The proposed project is directly relevant to military health, particularly those with TBI. The long range vision has the potential capacity to impact military and civilians. The addition of an EMR flag could potentially make providing care and caregiving easier. The study clearly describes potential pitfalls and proposed solutions.

Weaknesses: No weaknesses were noted.

Scientist Reviewer B

Strengths: The anticipated outcomes and/or knowledge products from the project will have a significant scientific impact and improve the treatment of psychological health conditions such as depression and PTSD and/or TBI.

Weaknesses: The proposed research is much more responsive to the health care needs of veterans compared to those of active duty service members.

Consumer Reviewer

Strengths: The project includes a significant focus on community-based input from service members, veterans, and family members. The project addresses improving the known negative impact of TBI on health care participation by engaging a supportive person. The project seeks to improve access to care across a health system (not just by individual providers).

Weaknesses: While there is a focus on concerns about patients who may not want the nudge on the record, there is not as much discussion about how providers (especially those without extensive

experience or knowledge of TBI and cognitive issues) will be trained to respond to the nudge in a way that impacts health care delivery and outcomes.

Regulatory Strategy and Transition Plan
Average Score: 8.0

Scientist Reviewer A

Strengths: There is a clear plan to bring the nudge to market at the VA and to pilot it.

Weaknesses: No weaknesses were noted.

Scientist Reviewer B

Strengths: The outcomes expected upon completion of the proposed research are very relevant, are measurable, and include some of the benefits to the intended end-user.

Weaknesses: Knowledge products are achievable, but the plan is very ambitious.

Technology Transfer Specialist Reviewer

Strengths: The applicant outlines the intellectual property rights and the government's retention of intellectual property as associated with the project. The schedule of milestones outlined gives a 4-year overview of activities. These activities are broken down for each of the aims of the project. These milestones are reasonable and achievable. All aims intend to share their results through publication and presentations as well as social media. The end user for all aims will be reached though these methods. Medical records flags are an important tool in the current health care model. Bringing more awareness to TBI for providers is valuable.

Weaknesses: Regulatory and personal hurdles associated with flagging EMR records in multiple systems are not adequately addressed. Risk analysis for cost outlines the cost associated with the TBI diagnosis and associated hospitalizations costs. The risks associated with the proposed funding strategy are not outlined as required.

Ethical Considerations
Average Score: 9.0

Scientist Reviewer A

Strengths: The study should not negatively impact anyone participating. Patients, providers, and caregivers are likely to benefit from this study. This study is minimal risk.

Weaknesses: Confidentiality and informed consent are not adequately addressed.

Scientist Reviewer B

Strengths: The population selected is highly relevant and will benefit from the results of the study.

Weaknesses: No weaknesses were noted.

Procurement Sensitive Document
Do not copy or distribute without CDMRP written permission.

Bioethicist Reviewer

Strengths: Overall, Project 1 has a comprehensive plan for ethically recruiting human subjects and maintaining safety. The study population is clearly articulated, along with the inclusion and exclusion criteria, supplemental documentation to describe the consent process (although not comprehensive), and sufficient and necessary measures for the protection of confidentiality and data management. A significant component to the description includes a data sharing plan, data capture, verification, and disposition.

There are several components to this project, including a survey and focus groups with professionals, and then focus groups with individuals with moderate to severe TBI and their TBI health care proxy partners. There is little difference in the human subjects protection approaches between working with professionals and individuals/families with TBI experiences, which will present some challenges.

In general, the proposed project is minimal risk where the benefits outweigh the potential risks (including those that have not been identified in this proposal). The study, in general, will not have a direct negative impact in the daily lives of human participants so long as proper informed consent processes are followed, participants are monitored during engagement in focus groups, and reasonable screening for capacity to consent is done with those participants who may be cognitively compromised due to TBI or have comorbid issues that could impact decision-making abilities.

Weaknesses: There is a plan to "over enroll underrepresented groups"; however, there is no description as to how this will be achieved in the recruitment process. The informed consent process for focus groups needs to involve a "process" where consent via phone and completion of a form is insufficient; a copy of the consent form as well as a review of the study procedures, risks, benefits, etc needs to occur at the start of the focus groups. A plan for voluntary refusal (not answering some focus group questions) or withdrawal from the focus group for whatever reason should be clear to participants, and this is not described in the consent form. The informed consent forms are very generic, and not all risks are described in the appropriate sections, including discomfort and the potential for focus group questions and disclosure of experiences to be emotionally triggering for some individuals. The duration of the actual component of the study the participant is voluntarily participating in should be described and not just the duration of the entire study. Study procedures are not comprehensive such as duration, and the language of the consent forms for nonprofessionals needs to be adjusted for comprehension.

Appropriate screening measures need to take place in the recruitment process of TBI patients and their families; not all moderate-to-severe TBI patients may have the capacity to give informed consent or may have comorbid conditions that impair decision-making abilities. Appropriate monitoring during focus groups ought to be done with a clear plan in place if participants experience triggering emotions (and focus group researchers should be qualified to detect discomfort among participants, be sensitive to triggering questions and responses, and act responsibly if the participant describes self-harm, which is not identified in the project description but in some consent forms). Health professionals who may have colleagues or know others in focus groups can equally feel discomfort in disclosing perspectives; efforts should be made to organize focus groups with this issue in mind and encourage privacy. Additionally, while the focus groups will be deidentified, there is the possibility that other focus group participants can identify others and due to the lack of anonymity, risks of confidentiality should be clarified with these methods, and instructions ought to be provided at the start of each focus group to encourage others to maintain privacy in the event they know each other.

Statistical Plan
Average Score: 7.6

Procurement Sensitive Document
Do not copy or distribute without CDMRP written permission.

Scientist Reviewer A

Strengths: This is not a randomized clinical trial. There is no reason to justify not randomizing.

Weaknesses: No weaknesses were noted.

Scientist Reviewer B

Strengths: Data analysis plans are suitable for the proposed project.

Weaknesses: The validation process does not have a specific statistical hypothesis.

Biostatistician Reviewer

Strengths: The proposed analytic procedures supporting the design and development of a cognitive nudge intervention are adequate. The analyses for the survey, focus group, and individual interview data are acceptable. Rapid matrix analysis with thematic analysis is a method for analyzing qualitative data in a time-sensitive manner to identify key themes and is suited to focused questions or problems in need of actionable knowledge, as per the present study (eg, rather than theory development). The research team and stakeholders will review the codes and themes being developed, which will improve the rigor and validity of the Aim 1 analysis.

Weaknesses: The study design and analysis of the validation are weak (Aim 3), involving a 1-group pilot trial of the intervention. Data collection involves semistructured interviews and/or focus groups with stakeholders (health providers, persons with TBI who received health care during the period, and their health care proxies, eg, family member) to (1) identify facilitators and barriers to implementation, (2) identify whether the intervention enhanced health care proxy engagement during the pilot, and (3) inform revisions of the intervention. No objective outcomes are involved, no specific qualitative analysis methodology is identified, and there are no quantitative data. A more suitable design would be a parallel-group randomized controlled trial (RCT)/cluster-randomized trial (CRT) comparing intervention to no intervention, which uses objective outcome measures (eg, health proxy attendance, satisfaction, treatment attendance and engagement measures, treatment outcome), and statistically tests the hypothesis that the intervention would significantly improve outcomes beyond no intervention; or a smaller feasibility clinical trial to inform the design of a future larger clinical trial. The validation analysis is subject to bias and ought not to be used as a basis for dissemination as planned.

Personnel
Average Score: 9.5

Scientist Reviewer A

The project leader, Kristen Dams O'Connor, received a PhD in counseling psychology from the University at Albany in 2008 and is Jack Nash Professor of Rehabilitation Medicine, vice chair of research, professor of neurology, and director of Brain Injury Research Center at Icahn School of Medicine at Mount Sinai.

Coleader Marc Silva received a PhD in counseling psychology from Marquette University in 2011 and is neuropsychologist and assistant professor of internal medicine at the University of South Florida.

Coleader Risa Richardson received a PhD in clinical psychology from West Virginia University in 2000 and is professor, clinician investigator, and director of sleep medicine research in the Morsani College of

Medicine, University of South Florida, and senior research director, Defense Health Agency TBI Center of Excellence at James A. Haley Veterans Hospital.

Strengths: The research team is highly regarded in the field and has the experience necessary to carry out the proposed project. Effort proposed is adequate to achieve proposed goals.

Weaknesses: No weaknesses were noted.

Scientist Reviewer B

Strengths: The research team including project leaders is highly qualified.

Weaknesses: No weaknesses were noted.

Community-Based Participatory Research
Average Score: 9.8

Scientist Reviewer A

Strengths: CBPR is incorporated throughout the planning and execution of the entire project. Dissemination is planned to include all stakeholders—patients, families, and providers.

Weaknesses: No weaknesses were noted.

Scientist Reviewer B

Strengths: The CBPR is appropriate and includes representatives.

Weaknesses: No weaknesses were noted.

Data and Research Resources Sharing Plan
Average Score: 8.8

Scientist Reviewer A

Strengths: The overall application indicates data will be shared as appropriate. Common data elements (CDE) are not applicable here.

Weaknesses: No weaknesses were noted.

Scientist Reviewer B

Strengths: There is a sensible plan for data and resources generated during the performance of the project in terms of how to share this information with the research community, including the sharing of deidentified data with data repositories.

Weaknesses: No weaknesses were noted.

Procurement Sensitive Document
Do not copy or distribute without CDMRP written permission.

Project 2: Provider Toolkit for Accommodating Cognitively Impaired Persons in Evidence-Based Treatments

OVERVIEW

The leaders of this project propose to develop a toolkit of specific accommodation strategies, reviews of the literature, and specific guidance for managing cognitive limitations in delivering evidence-based behavioral health care containing strategies consolidated from practice-based surveys, evidence-synthesis, and validation by clinicians and persons with lived experience of TBI. The specific aims are to (1) (discover) conduct an environmental scan of existing recommendations and evidence-based behavioral treatment (EBT) adaptations for persons with cognitive impairments; (2) (define and develop) engage the Community Engagement Council in prototyping the Provider Toolkit for Accommodating Cognitively Impaired Persons by defining its scope, elements, and function; and (3) (validate) activate the Community Engagement Council to conduct a formal review of the Provider Toolkit for Accommodating Cognitively Impaired Persons prototype to validate its design and identify implementation strategies.

	Average Score	Standard Deviation
**Overall Evaluation**<br>*Rating Scale: 1.0 (highest merit) to 5.0 (lowest merit)*	1.8<br>**(Excellent)**	0.1
**Criteria**<br>*Rating Scale: 10 (highest merit) to 1 (lowest merit)*	**Average Score**	
**Research Strategy and Feasibility**	8.3	
**Impact and Relevance to Military Health**	7.6	
**Regulatory Strategy and Transition Plan**	7.2	
**Statistical Plan**	7.2	
**Personnel**	8.0	
**Data and Research Resources Sharing Plan**	9.0	

SCORED CRITERIA

Research Strategy and Feasibility
Average Score: 8.3

Scientist Reviewer A

The team will employ a 3-step process to carry out the proposed aims. First, they will conduct an environmental scan to determine existing provider resources and compile identified resources into a product grid. Second, they will have a team of subject matter experts and a team of lived experience partners evaluate the products to be included in the toolkit. Finally, they will form a community engagement council to review the prototype of the toolkit. Analyses include triangulation and an iterative data analysis approach to examine and synthesize qualitative data into feedback for each next step.

Strengths: The goal of creating a provider toolkit for adapting evidence-based therapies for conditions commonly comorbid with TBI is quite feasible, and the timeline seems appropriate. The investigators have effectively described a thorough and logical plan for achieving their objectives. The proposed

approach is iterative and community-engaged at each step, and it is well-aligned with the goals of both the specific project and the overarching program.

Weaknesses: All identified weaknesses are fairly minor. First, it is not entirely clear from the project description whether the toolkit will be intended to be used for all people with cognitive impairments, or just those with TBI, which will need to be clarified before the project is undertaken. Next, the investigators have not fully described how they will ensure that all potential existing tools will be identified; it seems that their plan of surveying their professional contacts may not be sufficient. Further, it is unclear who will complete the anonymous Qualtrics survey that queries providers about resources, how the team will ensure a broad reach of providers across numerous areas in behavioral health, or how providers will be incentivized/compensated for completing the Qualtrics survey. Next, the metrics by which items will be evaluated for inclusion in the toolkit are not thoroughly described. For example, it is unclear whether selection of toolkit items by the subject matter experts (SMEs) will be based on the evidence base for a specific adaptation, their own clinical/research experiences with similar tools, face validity/feasibility of an adaptation, or some other criteria. Similarly, the process by which content for any missing/needed products will be conducted is not thoroughly described. Depending on the findings, this could be a lengthy undertaking, but less than a single year is allotted in the timeline. Finally, it is unclear how (in Aim 3) the provider SME review panel will validate the toolkit. It seems that validation of the toolkit items could be a monumental task if it were to be approached thoroughly.

Scientist Reviewer B

Strengths: The literature review provides the basis for the study aims and hypotheses. The proposed project is directly relevant to the FPA. The purpose and objectives of the study and the specific aims are well described and aligned with the tasks in the scope of work. The design of the study will achieve the specific aims. Based on the letters of support and past experience, the study team seems to have access to the needed samples/populations. The proposal identifies potential pitfalls and solutions. The proposed project appears to be feasible and achievable within the timeline proposed.

Weaknesses: The approach to review is not systematic.

Impact and Relevance to Military Health
Average Score: 7.6

Scientist Reviewer A

Strengths: TBI is a major problem for military, veteran, and civilian populations, and if the goals of this project are achieved, they could have a substantial positive impact across each of these sectors. The success of this project could effectively increase the pool of providers who are able to competently treat co-occurring mental health problems in the context of TBI by amassing all existing resources and compiling them in a provider-friendly format. This toolkit would be especially useful for providers who have limited experience working with TBI populations and may not be aware of how to identify them or adapt treatment for them.

Weaknesses: The development of a provider toolkit does seem to be an important initial step, but it may not be going far enough. Giving providers the tools may not be enough to ensure uptake and correct usage of these tools. It seems it would be important to include aspects of training for use of the tools by providers unfamiliar with the TBI population.

Procurement Sensitive Document
Do not copy or distribute without CDMRP written permission.

Scientist Reviewer B

Strengths: The project focuses on veterans with TBI. The long-range vision will benefit providers and individuals with TBI. If the scan turns up evidence based treatments, providing adaptations for individuals with cognitive disabilities will improve upon what is currently available. Pitfalls and solutions are discussed.

Weaknesses: There may not be enough provider input.

Consumer Reviewer

Strengths: The project is focused on the challenges some service members and veterans with TBI experience when accessing evidence-based behavioral treatments with a goal of improving effectiveness of this care for this population. The project builds upon existing knowledge of evidence-based treatments and adaptations.

Weaknesses: The proposal includes a significant emphasis on input from patients and subject matter experts. It does not reference provider input specifically related to uptake and implementation of knowledge based on a toolkit. The topics discussed for the toolkit may require extensive training for providers to effectively use them in practice, which could potentially limit the long-term impact of the project if there are eventual challenges with uptake. Input like this from end-user providers could inform important things like the length and format of the toolkit content.

Regulatory Strategy and Transition Plan
Average Score: 7.2

Scientist Reviewer A

Strengths: The investigators outline a thorough transition plan, and the team has demonstrated the ability to provide deliverables. The timeline for providing deliverables and moving to the next phase of development seems realistic. No regulatory strategy is provided as it is not applicable.

Weaknesses: It is unclear who will have access to the toolkit housed on the Toolshed (ie, whether this will be publicly available or require VA/DOD or other credentials for access). It is not clear how funding will transition after the grant period has ended. There is no plan detailed for the sustainability of the product—presumably updates in the research space will necessitate periodic updates to the toolkit, but there is limited detail on how development will continue. Additionally, cost analyses are not included in the current proposed project (only suggested to be included in future efforts), so it is difficult to evaluate the potential cost impacts.

Scientist Reviewer B

Strengths: FDA regulation is not applicable to this project. The outputs are relevant and include the end user. The plan is to deliver the toolkit to market through the toolbox open to the general public. The toolkit will include provider training.

Weaknesses: No clear plan for sustainability and measuring uptake is provided.

Technology Transfer Specialist Reviewer

Strengths: The applicant outlines the intellectual property rights and the government's retention of intellectual property as associated with the grant. The schedule of milestones outlined gives a 4-year overview of activities. These activities are broken-down for each of the aims of the project. These milestones are reasonable and achievable. They intend to share their results through publication and presentations as well as social media. The end user for all aims will be reached though these methods. Project 2 addresses the need for developing products that address disabilities. The development of this knowledge tool will make an impact on the treatment for many patients.

Weaknesses: Risk analysis for cost outlines the cost associated with the TBI diagnosis and associated hospitalizations costs. The risks associated with the proposed funding strategy are not outlined as required.

Statistical Plan
Average Score: 7.2

Scientist Reviewer A

Strengths: The investigators describe that they will use triangulation and an iterative data analysis to generate insight from qualitative data, which seems appropriate for the objectives of this project.

Weaknesses: The method for qualitative data analysis is minimally described which makes it difficult to evaluate the quality of the statistical plan. However, this is a relatively small part of the project which has limited impact on the ability to achieve the project goals.

Scientist Reviewer B

Strengths: The statistical model and data analysis plan are suitable for the planned project. Subjects will not be randomized, and no one will be blinded which is appropriate for the research proposed. The plan is to reach saturation which is appropriate for this type of study. The proposed study is not a phase 3 clinical trial; however, findings will be discussed in terms of sex, gender, race/ethnicity, job title, clinic type, and whether it is a civilian or VA facility.

Weaknesses: It is not clear how the toolkit will be validated.

Biostatistician Reviewer

Strengths: The environmental scan methodology will enlist a literature research and survey to identify resources. Reviews of the resources provided by policy and professional organization, health care provider, and lived experience persons will be triangulated and synthesized using "Rapid assessment process" methodology, a rapid, economical, analysis of team-based mixed-methods data, with a track record of application to health issues. Trustworthiness (validity) and dependability (reliability) of the findings can be negatively impacted by sources of bias (ie, observer bias, selection of participants). Iteration and triangulation are 2 procedures mentioned by the applicants that will enhance these properties.

Weaknesses: Mention is made of "rating materials," but the rating domains are narrow and may not cover potential harms. How the evidence base pertaining to a resource will be taken into consideration is unclear. Inclusion and exclusion criteria would help to organize the environmental scan literature search. The literature search is not a systematic search, which is likely to result in accidentally missing relevant

sources of information. Basic descriptive statistics could be used to categorize and summarize the identified resources (ie, type of psychological health problem; type of service setting; health professional type targeted by the resource; intervention approach) and to identify gaps (eg, PMID: 35627456). The number of participants in the expert group lists (policy and professional organization, health care provider, and lived experience) is rather small, so saturation of themes may not be achieved. The participants may not have sufficient expertise in particular psychological health phenotypes or evidence-based medicine methodologies. This could pose problems when recommending resources (eg, if a treatment manual has an adaptation, the adaptation may seem appropriate, but the treatment approach may lack efficacy for the phenotype or may not have been validated; an assessment with good face validity may have poor psychometric properties). The analysis approach will result in resources being "vetted" but does not establish or evaluate the impact of any resource on TBI-related psychological health outcomes.

Personnel
Average Score: 8.0

Scientist Reviewer A

The project leader, Jeanne Hoffman, received a PhD in clinical psychology from Arizona State University in 2000 and is professor and head of the Division of Rehabilitation Psychology and Neuropsychology for the Department of Rehabilitation Medicine at the University of Washington.

Coleader Aaron Martin received a PhD in clinical psychology from Virginia Commonwealth University in 2013 and is clinical psychologist, Mental Health and Behavioral Science Service, Pain Psychology Section, James A. Haley VA Hospital, and assistant professor of psychiatry and behavioral neurosciences, University of South Florida.

Strengths: The assembled team is well rounded in both TBI and adaptations of behavioral interventions for conditions commonly comorbid to TBI. Dr Hoffman (the lead of this project) is a well-known and highly respected expert in this domain.

Weaknesses: The personnel include expertise in sleep, but it is not clear if there is specific expertise in insomnia and nightmares, which are two of the most common sleep disorders seen in a TBI population (particularly in service members and veterans).

Scientist Reviewer B

Strengths: The project leader is a well-experienced PI. The levels of effort are appropriate. The team has direct experience with veterans.

Weaknesses: The team may lack nightmare and insomnia experts.

Data and Research Resources Sharing Plan
Average Score: 9.0

Scientist Reviewer A

Strengths: Deidentified data will be shared with the research community in relevant biorepositories.

Weaknesses: No weaknesses were noted.

Procurement Sensitive Document
Do not copy or distribute without CDMRP written permission.

Scientist Reviewer B

Strengths: The goal of the project is to share the toolkit widely. CDE are not relevant to this project.

Weaknesses: No weaknesses were noted.

Project 3: Empowering Teams to Implement Evidence-Based Behavioral Interventions (TEAM-BI)

OVERVIEW

The leaders of this project propose to develop a playbook for implementing an interdisciplinary program in TBI inpatient rehabilitation settings to reduce challenging behavior of patients with TBI. The specific aims are to (1) (discover, define, and develop) codesign a Team-Based Behavioral Interventions Playbook through engagement of rehabilitation team members, persons with brain injury, and family members/care partners to identify areas of the Staff Training in Assisted Living Residences (STAR-VA) manual that need adaptation, as well as gap areas in need of further development, and (2) (validate) validate, refine, and disseminate the playbook.

	Average Score	Standard Deviation
**Overall Evaluation**<br>*Rating Scale: 1.0 (highest merit) to 5.0 (lowest merit)*	1.9<br>**(Excellent)**	0.3
**Criteria**<br>*Rating Scale: 10 (highest merit) to 1 (lowest merit)*	**Average Score**	
**Research Strategy and Feasibility**	8.8	
**Impact and Relevance to Military Health**	7.6	
**Regulatory Strategy and Transition Plan**	8.2	
**Ethical Considerations**	8.4	
**Statistical Plan**	6.6	
**Personnel**	9.8	
**Community-Based Participatory Research**	9.5	
**Data and Research Resources Sharing Plan**	9.0	

SCORED CRITERIA

Research Strategy and Feasibility
Average Score: 8.8

Scientist Reviewer A

The study will build on the STAR-VA handbook which is a guide for handling behavior issues in individuals with dementia. The team will, with the help of veterans design a playbook for rehabilitation professionals working with individuals with TBI. The team will then validate the playbook.

Strengths: The scientific rationale and literature review provide the basis for the study aims and hypotheses. The proposed project is directly relevant to the FPA. The purpose and objectives of the study and the specific aims are well described and aligned with the tasks in the scope of work. The design of the study will achieve the specific aims. Based on the letters of support and past experience, the study team seems to have access to the needed samples/populations. The proposal identifies potential pitfalls and solutions. The proposed project appears to be feasible and achievable within the specified time frame. Veterans will be consulted on the design of the playbook.

Weaknesses: No weaknesses were noted.

Scientist Reviewer B

Strengths: The scientific rationale for the TeamBI playbook is based on a review of the literature in this area, the presentation of preliminary data from a national survey of 300 providers, 28 Polytrauma Rehabilitation Center staff, and a similar program that addresses similar issues for patients with dementia. The objectives, aims, and hypotheses formulated in the proposed project are well described and are aligned with the tasks in the statement of work. Hypotheses are well formulated and supported by data and evidence from the literature. There is a systematic approach in the sequencing of the 3 aims. The experimental design, data collection measures, sample size consideration, and tools as well as study procedures are very relevant to the completion of the different aims of the project. For instance, the inclusion criteria for the focus are well described and enhance the rigor of the study. The timing for the implementation of different aims is reasonable. Researchers have access to necessary facilities and resources. The project will include an interdisciplinary team.

Weaknesses: Some level of extrapolation will be needed given that the TeamBI playbook will be based on an intervention implemented in a population that faces greater challenges of maladaptive behavior compared to those faced by patients with TBI.

Impact and Relevance to Military Health
Average Score: 7.6

Scientist Reviewer A

Strengths: This project will have an extensive impact and will make significant scientific advances. The project represents an improvement to clinical practice guidance. Pitfalls and possible solutions are discussed. This is directly related to veterans and will include veterans in the design process.

Weaknesses: No weaknesses were noted.

Scientist Reviewer B

Strengths: If successful, findings from this project will have significant practical implications and high translational value for this segment of patients with TBI.

Weaknesses: Although the impact of maladaptive disorders on the patient population is devastating, the population itself is not that large.

Consumer Reviewer

Strengths: The project is focused on maladaptive behaviors in the rehabilitation setting, which can be a barrier to care. It also addresses workforce development and sustainment, which affects the quality of care provided. The project builds upon an existing evidence-based program for the dementia population.

Weaknesses: Since the project focuses on TBI rehabilitation programs specifically (vs nonspecialized health care settings) it is unclear how wide the gap in knowledge and operations is between the current state and the end state goal with the training. Since these maladaptive behaviors are known to occur with patients with TBI, it may be that these specialized programs are already equipped to address the behaviors (in a way that general rehabilitation programs might not be).

Regulatory Strategy and Transition Plan
Average Score: 8.2

Scientist Reviewer A

Strengths: FDA regulation is not applicable. The proposed project will provide training materials for rehabilitation professionals. The schedule and milestones are realistic and feasible as proposed. The end user is part of the process throughout. The funding strategy to bring outcomes to market (eg, training materials) is reasonable and achievable. The plan for development of the practice guidelines, publications, etc, is achievable. Cost, schedule, creation of the training materials, and sustainability are realistic and reasonable.

Weaknesses: No weaknesses were noted.

Scientist Reviewer B

Strengths: The project describes very well and provides a comprehensive intellectual and material property plan, and upon completion the outcomes from the playbook are highly measurable.

Weaknesses: The risk analysis for transition is not adequately described.

Technology Transfer Specialist Reviewer

Strengths: The applicant outlines the intellectual property rights and the government's retention of intellectual property as associated with the grant. The schedule of milestones outlined gives a 4-year overview of activities. These activities are broken-down for this project. These milestones are reasonable and achievable. The project leaders intend to share their results through publication and presentations as well as social media. The end user for the project will be reached though these methods. Project 3 has included time for regulatory issues in the timeline.

Weaknesses: Risk analysis for cost outlines the cost associated with the TBI diagnosis and associated hospitalizations costs. The risks associated with the proposed funding strategy are not outlined.

Ethical Considerations
Average Score: 8.4

Scientist Reviewer A

Strengths: This is not a clinical trial. The population selected will benefit from the study. This is minimal risk.

Weaknesses: Confidentiality and informed consent are not sufficiently discussed.

Scientist Reviewer B

Strengths: The concerned population will greatly benefit from the successful implementation of the project.

Weaknesses: The target patient population is particularly vulnerable and fragile. It is not clear that the knowledge product from this project will be low risk.

Procurement Sensitive Document
Do not copy or distribute without CDMRP written permission.

Bioethicist Reviewer

Strengths: In general, the project has considered several protections to minimize potential risks or harms to participants. This is a minimal risk study if these and additional recommended protections are in place. The human subject plan emphasizes the importance of confidentiality, including when conducting focus groups. Data capture, storage, and management are sound, and appropriate measures are taken in sharing data. There are descriptions about the focus groups, including duration, supporting guides, and proper consent procedures in place. A trained facilitator in conducting the focus groups will be present; additional screening measures will be needed (see below) to ensure proper safeguards are in place for vulnerable populations. There are comprehensive descriptions of questionnaires and other research collection instruments that add to the clarity of the project.

Weaknesses: The informed consent process indicates that a screening consent form will be used and that no other consent needs to be revisited for a one-time survey with working clinicians. This looks like a cut and paste from previous sections and does not apply to this section on focus groups with veterans. There are several areas in the human subject recruitment and safety procedures that do not align with the methods being described.

A copy of the consent form as well as a review of the study procedures, risks, benefits, etc needs to occur at the start of the focus groups (this is not clear due to the consent description in the prior page that relates to phone consent). A plan for voluntary refusal (not answering some focus group questions) or withdrawal from the focus group for whatever reason should be clear to participants, and this is not described in the consent form. The informed consent forms are very generic, and not all risks are described in the appropriate sections, including discomfort and the potential for focus group questions and disclosure of experiences to be emotionally triggering for some individuals. The description of the focus groups is very comprehensive in Project 3, aside from the lack of risks described, and such descriptions should be in the actual consent forms; the consent forms are not as robust.

Appropriate screening measures need to take place in the recruitment process of TBI patients and their families; not all moderate-to-severe TBI patients may have the capacity to give informed consent or may have comorbid conditions that impair decision-making abilities. Appropriate monitoring during focus groups ought to be done with a clear plan in place if participants experience triggering emotions (and focus group researchers should be qualified to detect discomfort among participants, be sensitive to triggering questions and responses, etc). A guidebook is provided, and it appears as though focus group personnel are qualified in delivering and recording focus groups; guidance for recognizing risks associated with discomfort and other negative experiences, along with follow-up support, eg, reiterating contact information, encouraging reports of any adverse events, etc, is needed.

Recognition of the vulnerability of the population of veterans with TBI is needed. There are risks to this study which need to be acknowledged, including the discomfort of reporting potentially unpleasant experiences. Physical and mental health comorbidities such as PTSD can exacerbate negative experiences of TBI and TBI rehabilitation. Persons with moderate to severe TBI have higher risk of anxiety, depression, and PTSD. Risks associated with privacy and confidentiality are also present.

Statistical Plan
Average Score: 6.6

Scientist Reviewer A

Strengths: The sample size was calculated to reach saturation and is appropriate. Randomization is not appropriate to this study. Blinding is also not appropriate.

Weaknesses: Sex/gender and race/ethnicity are not adequately addressed.

Scientist Reviewer B

Strengths: All statistical models and data analysis plans are well described and are highly appropriate for the project.

Weaknesses: It is not clear that the validation plan can be achieved using the proposed metrics.

Biostatistician Reviewer

Strengths: The sample size for the focus groups (Aim 3.1a) is reasonably justified. The rapid assessment method will be used to identify themes from focus groups and is an accepted method. The target sample size for the rehabilitation health care provider sample (Aim 3.2a) was informed by a power calculation. A repeated measures *t* test is suitable for testing whether health care provider perceptions of capability, opportunity, and motivation to implement the behavioral treatment change before and after the online training.

Weaknesses: The aims are somewhat ambiguous to interpret. If the aims are more about adapting the intervention for future pilot testing in a TBI population (not implied), or adapting the intervention, skipping validation, and disseminating, because experts believe the evidence base in another population is robust and translatable, then the research strategy and analyses are excellent. However, the aims do discuss validating the intervention, and in this regard the analyses and design are not adequate.

Several statistical and methodological concerns with the proposal are apparent: (1) the effect size used for the sample size calculation is somewhat arbitrary; (2) testing for statistically significant differences in health care provider outcomes across settings or facility types, as described, is not likely to be possible at the planned sample size; (3) the adaptation of the COM-B questionnaire (page 120 of the proposal) does not appear to measure the intended outcome (eg, rehabilitation professionals are asked, "I am psychologically able to change my behavior to improve my health" [capability scale], etc); (4) the extent to which health care providers complete the intervention (ie, read the playbook, watch the video) is unmeasured; similar study designs usually offer in-person training in an intervention to ensure exposure; (5) for health providers that begin the study with higher outcomes, ceiling effects will limit the ability to detect group changes over time; and (6) the study is described as a validation study that will support dissemination but does not address whether the intervention improves care and outcomes among TBI patients. Jumping from no pilot or validation data—despite stating this goal—to planned dissemination, is not appropriate.

Personnel
Average Score: 9.8

Scientist Reviewer A

The project leader, Jennifer Bogner, received a PhD from The Ohio State University in psychology in 1989 and is professor in physical medicine and rehabilitation at The Ohio State University. The coleader, Tracy Kretzmer, received a PhD in clinical psychology from the University of Alabama at Birmingham in 2000 and is clinical neuropsychologist in polytrauma/physical medicine and rehabilitation at James A. Haley Veterans Hospital.

Strengths: The team includes top researchers in the field. They have plenty of experience in TBI and veterans. Effort levels are appropriate for the scope of work.

Procurement Sensitive Document
Do not copy or distribute without CDMRP written permission.

Weaknesses: No weaknesses were noted.

Scientist Reviewer B

Strengths: This is a highly qualified research team, and they have a long history of collaboration and successful implementation of similar programs/interventions.

Weaknesses: No weaknesses were noted.

Community-Based Participatory Research
Average Score: 9.5

Scientist Reviewer A

Strengths: CBPR is clear throughout the planning and execution. The overarching dissemination plan is to reach all stakeholders.

Weaknesses: No weaknesses were noted.

Scientist Reviewer B

Strengths: CBPR participation is evident in the project.

Weaknesses: No weaknesses were noted.

Data and Research Resources Sharing Plan
Average Score: 9.0

Scientist Reviewer A

Strengths: The plan is to share the playbook through the toolbox accessible to the general population. CDE are not applicable to this project.

Weaknesses: No weaknesses were noted.

Scientist Reviewer B

Strengths: There is a sensible plan for data and resources generated during the performance of the project. They will be shared with the research community, including the sharing of deidentified data with data repositories.

Weaknesses: No weaknesses were noted.

Procurement Sensitive Document
Do not copy or distribute without CDMRP written permission.

Project 4: Data-Driven Policy Recommendations for Virtual Health Care for Persons With TBI Morbidity

OVERVIEW

The leaders of this project propose a human-centered design (HCD) and CBPR approach with sequential mixed methods to inform the appropriate use of virtual health care resources with persons with TBI in this 2.5-year implementation study. This proposed project will leverage existing data archived from a multicenter-funded study of TBI health care needs. The specific aims are to (1) (discover/define—secondary analyses) characterize data (facilitators and barriers) and develop a taxonomy to inform virtual health care delivery for persons with TBI morbidity; (2) (develop—stakeholder-driven products and dissemination plan development) engage stakeholders to identify necessary accommodations and recommendations to inform development of clinical and policy decision support for virtual health resource use with persons with TBI morbidity; and (3) (validate—products and dissemination plan) re-engage community engagement council policy and professional partners (CEC PPP), ie, key policymakers in DOD, VA, and civilian health care systems, to inform the HCD approach to creating stakeholder-driven products and a dissemination plan to support data-driven virtual health resource for use with persons with TBI morbidity.

	**Average Score**	**Standard Deviation**
**Overall Evaluation**<br>*Rating Scale: 1.0 (highest merit) to 5.0 (lowest merit)*	1.5<br>**(Outstanding)**	0.1
**Criteria**<br>*Rating Scale: 10 (highest merit) to 1 (lowest merit)*	**Average Score**	
**Research Strategy and Feasibility**	9.3	
**Impact and Relevance to Military Health**	8.6	
**Regulatory Strategy and Transition Plan**	8.2	
**Statistical Plan**	8.4	
**Personnel**	9.8	
**Data and Research Resources Sharing Plan**	8.8	

SCORED CRITERIA

Research Strategy and Feasibility
Average Score: 9.3

Scientist Reviewer A

The team will engage in 3 stages of discover/define, develop, and validate to carry out the proposed aims. First, they will use an existing data set to characterize facilitators and barriers, develop a codebook with stakeholder input, and analyze data, and then create summaries of the data and develop a taxonomy of results. Second, they will engage stakeholders to develop products and a dissemination plan by conducting a gap analysis, engaging a stakeholder panel to gain feedback on an initial draft of the products and dissemination plan, and based on that feedback develop the products and dissemination plan. Finally, they will engage policymakers from VA, DOD, and civilian sectors to compile and provide

feedback on the products that are developed, and finalize these products. They will use qualitative data analysis methods to iteratively code and refine coding of interview transcripts.

Strengths: The investigators have proposed a detailed and thorough plan for developing data-based products and a dissemination plan to inform policy recommendations for virtual health care delivery to individuals with TBI. The investigators already have access to the data set that will form the foundation of this project, which will speed initiation of the project. This is a feasible research plan, and the timeline seems reasonable to accomplish the proposed aims. The proposed approach is iterative and community-engaged at each step, and it is well-aligned with the goals of both the specific project and the overarching program.

Weaknesses: It is unclear what will be done if no consistent or helpful themes/findings emerge from the initial secondary data analysis (Aim 1). It seems this is unlikely, but given that the original data were collected for another purpose, it is a possibility. It also seems that 90 minutes may not be enough time for the stakeholder meetings as such a large team of stakeholders may not quickly come to consensus.

Scientist Reviewer B

Strengths: Because of a lack of research focusing on the virtual health care resource needs for patients diagnosed with TBI the current project aims to leverage existing qualitative data to develop a taxonomy of domains and themes relevant to access to virtual health care for persons with TBI morbidity. The scientific rationale to develop a taxonomy of domains and themes relevant to access to virtual health care for persons with TBI morbidity is well presented and relies on several tested programs and interventions in the area.

There are well described objectives, aims, and hypotheses, which are aligned with the tasks in the statement of work. Using HCD and CBPR approach, using sequential mixed methods, the experimental design, data collection measures, sample size consideration, and tools as well as study procedures are very relevant to the completion of the different aims of the project. There is reasonable timing for the implementation of the project's aims.

Weaknesses: The sample size for focus group participants might be too small.

Impact and Relevance to Military Health
Average Score: 8.6

Scientist Reviewer A

Strengths: If successful, this project could have a substantial impact on policies surrounding virtual health care for individuals with TBI across VA, DOD, and civilian sectors. This project is in direct response to a perceived need for specific accommodations and recommendations for the TBI population and will be responsive to stakeholders' needs at multiple levels.

Weaknesses: The project could potentially increase its impact by broadening scope to include not only virtual health care-related policy recommendations but also in-person recommendations. Although it appears other projects in this proposal do address in-person modalities to some extent, it does not appear that these projects approach the policy-level challenges that this project does. However, that change would change the methodology needed, as it appears the existing data only covers virtual health care challenges.

Scientist Reviewer B

Strengths: The project will likely have significant impact in terms of improved access to care in the DOD/VA, if successful.

Weaknesses: No weaknesses were noted.

Consumer Reviewer

Strengths: The project focuses on the use of virtual health care for patients with TBI. Virtual care is an increasingly important component of access to care for many patients, and the project will increase knowledge and understanding of how and when virtual care can be used effectively with these patients. The project includes a focus on developing policy to support implementation of findings.

Weaknesses: No weaknesses were noted.

Regulatory Strategy and Transition Plan
Average Score: 8.2

Scientist Reviewer A

Strengths: The investigators outline a thorough transition plan, and the team has demonstrated the ability to provide deliverables. The timeline for providing deliverables and moving to the next phase of development seems realistic. No regulatory strategy is provided as it is not applicable.

Weaknesses: No weaknesses were noted.

Scientist Reviewer B

Strengths: The transition plan as outlined is mostly appropriate.

Weaknesses: No weaknesses were noted.

Technology Transfer Specialist Reviewer

Strengths: The applicant outlines the intellectual property rights and the government's retention of intellectual property as associated with the grant. The schedule of milestones outlined gives an overview of project activities. These activities are broken-down for each of the projects. These milestones are reasonable and achievable. All 4 projects intend to share their results through publication and presentations as well as social media. The end user for all aims will be reached though these methods.

Virtual medicine will only expand and reach more patients. Project 4 can address these needs for the TBI population.

Weaknesses: Risk analysis outlines the cost associated with the TBI diagnosis and associated hospitalizations costs. The risks associated with the proposed funding strategy are not outlined.

Statistical Plan
Average Score: 8.4

Scientist Reviewer A

Strengths: The investigators describe a thorough qualitative data analysis process that considers stakeholder feedback at multiple points. The team is well-qualified to carry out these analyses, and it seems that it will be relatively straightforward given that the data are already collected.

Weaknesses: No weaknesses were noted.

Scientist Reviewer B

Strengths: All methods appear to be appropriate.

Weaknesses: No weaknesses were noted.

Biostatistician Reviewer

Strengths: For Aim 1, the qualitative data analysis methodology is suitable. Achieving 80% interrater reliability of the coding scheme, and developing codes in consultation with policy, professional organization, health care providers, and lived experience stakeholders, are quality assurance procedures to improve accuracy and consistency. The applicants provide some reasoning from a published paper that the fixed sample size will be sufficient to allow for saturation of themes. Iteratively circling back to the stakeholders to discuss the summarized findings and their views on the key implications (ie, messaging, policy implications) is an impressive step.

Weaknesses: Aim 1 is a qualitative investigation of the barriers and facilitators to virtual health care delivery among TBI patients with comorbid pain. It does not involve data collection; it uses a secondary data set which raises concerns, as participants' responses may not align with the research questions.

Aims 2 and 3, with focus on interpreting and conveying the findings from Aim 1, are truly research goals in and of themselves. Aim 2 describes planning a "product grid" (eg, executive summary, presentation slide deck, white paper) and taking "a rapid systematic approach to finalizing the product grid, products, and dissemination plan." These aims are basically a dissemination plan. Aim 3 is presented as the "validation" component and describes seeking feedback on the proposed products and dissemination activities from stakeholders through semistructured interviews, with a view to finalizing recommendations for adoption and policy change. While some elements of these procedures, such as the use of semistructured interviews and rapid content analysis, increase replicability, there are no overarching research goals or analyses to the aims.

Personnel
Average Score: 9.8

Scientist Reviewer A

The project leader, Jolie Haun, received a PhD in health education and behavior from University of Florida in 2007. Dr Haun is currently an adjunct associate professor within the Division of Epidemiology in the Department of Internal Medicine at the University of Utah. The coleader, Bridget Cotner, received a PhD in measurement, research, and evaluation from the University of South Florida in 2014 and is currently research health science specialist at the James A. Haley Hospital and Clinics.

Strengths: The assembled team is experienced in conducting similar projects and completing dissemination of said products within health care systems. Dr Haun is an experienced and respected

Procurement Sensitive Document
Do not copy or distribute without CDMRP written permission.

expert in implementation science, and Dr Cotner is an impressive early career scientist with relevant expertise.

Weaknesses: No weaknesses were noted.

Scientist Reviewer B

Strengths: The project has a highly qualified team with a strong history of collaboration.

Weaknesses: No weaknesses were noted.

Data and Research Resources Sharing Plan
Average Score: 8.8

Scientist Reviewer A

Strengths: Deidentified data will be shared with the research community in relevant biorepositories.

Weaknesses: No weaknesses were noted.

Scientist Reviewer B

Strengths: There is a sensible plan for data and resources generated during the performance of the project to be shared with the research community, including the sharing of deidentified data with data repositories.

Weaknesses: It is not clear that CDE will be utilized.

Procurement Sensitive Document
Do not copy or distribute without CDMRP written permission.
